# Novel Lomefloxacin–Triazole Hybrids: Design, Synthesis and In Vitro Antiproliferative Activity in Breast, Melanoma and Colon Cancer Cell Lines

**DOI:** 10.3390/biom16091323

**Published:** 2026-09-11

**Authors:** Justyna Nowakowska, Zuzanna Rzepka, Andrzej Bak, Dorota Wrześniok, Krzysztof Marciniec

**Affiliations:** 1 Doctoral School, Medical University of Silesia, Poniatowskiego 15, 40-055 Katowice, Poland; 2 Student Scientific Group, Department of Organic Chemistry, Faculty of Pharmaceutical Sciences in Sosnowiec, Medical University of Silesia, Jagiellońska 4, 41-200 Sosnowiec, Poland; 3 Department of Pharmaceutical Chemistry, Faculty of Pharmaceutical Sciences in Sosnowiec, Medical University of Silesia, Jagiellońska 4, 41-200 Sosnowiec, Poland; zrzepka@sum.edu.pl (Z.R.); dwrzesniok@sum.edu.pl (D.W.); 4 Institute of Chemistry, University of Silesia, Szkolna 9, 40-007 Katowice, Poland; andrzej.bak@us.edu.pl; 5 Department of Organic Chemistry, Faculty of Pharmaceutical Sciences in Sosnowiec, Medical University of Silesia, Jagiellońska 4, 41-200 Sosnowiec, Poland

**Keywords:** lomefloxacin, molecular docking, molecular dynamics simulations, triazole hybrids, CuAAC, WST-1 assay

## Abstract

In recent years, extensive research has focused on fluoroquinolone–triazole hybrids, with particular emphasis on structural modifications of ciprofloxacin. In the present study, novel derivatives of lomefloxacin with a 1,4-disubstituted-1,2,3-triazole moiety were designed, synthesised, and biologically evaluated at the N4 position of the piperazine ring. Molecular docking studies suggested potential interactions of the designed compounds with topoisomerase I, with docking scores comparable to that of the reference compound camptothecin. Molecular dynamics simulation suggested short-term conformational stability of the ligand-based (holo) topoisomerase IIβ modulator. The synthesis was accomplished using a copper(I)-catalysed azide–alkyne cycloaddition, leading to the formation of nine new compounds. A preliminary evaluation of antiproliferative activity was performed on selected human cancer cell lines using the WST-1 assay, while selectivity against normal human dermal fibroblast was assessed. The results obtained revealed promising antiproliferative activity of the tested derivatives, particularly against colorectal cancer cell lines.

## 1. Introduction

Aromatic heterocyclic compounds containing heteroatoms constitute one of the fundamental pillars of modern medicinal chemistry because of their broad application in the design of anticancer agents. Particular attention has been paid to quinoline and its derivatives, as well as 1,2,3-triazoles, which frequently serve as key structural motifs in newly synthesised compounds exhibiting antiproliferative activity [1,2,3,4,5,6]. Both systems are characterised by favourable physicochemical properties and the ability to interact with diverse molecular targets in cancer cells. Quinoline derivatives, as well as compounds bearing the 1,2,3-triazole ring, exhibit an optimal balance between lipophilicity (logP), polarity, and polar surface area (PSA), which contributes to adequate membrane permeability and favourable bioavailability. The presence of nitrogen heteroatoms allows the formation of hydrogen bonds, π–π stacking interactions, and electrostatic contacts with biological macromolecules, thus enhancing the binding affinity to molecular targets involved in tumour progression [7,8,9,10].

In recent years, hybrid structures that combine fluoroquinolone fragments with the 1,2,3-triazole moiety have attracted considerable attention in medicinal chemistry and drug discovery. Integration of these pharmacophores within a single molecule often results in increased anticancer activity and improved pharmacokinetic profiles compared to the parent compounds [11,12]. Fluoroquinolones were originally recognised as antibacterial agents, exerting their effects through inhibition of bacterial topoisomerases, namely DNA gyrase and topoisomerase IV. Their molecular structure also allows interactions with eukaryotic topoisomerases, which have been proposed as one of the possible molecular mechanisms underlying their antiproliferative effects, before and after appropriate structural modifications [11,13]. For example, ciprofloxacin-1,2,3-triazole hybrids exhibited significant antiproliferative activity against lung cancer (A549), glioma (U-87MG) and breast cancer (MCF-7) cell lines, often with lower IC50 values than standard chemotherapeutics and a more favourable therapeutic index towards normal cells [14,15].

The mechanisms of action of fluoroquinolone/1,2,3-triazole hybrids are multifaceted and affect various stages of cancer cell proliferation and survival. Reported studies have suggested that interactions with topoisomerases I and II may contribute to their biological activity, potentially leading to DNA damage and suppression of replication processes in cancer cells [12,16]. In addition to these proposed interactions, these compounds also inhibit tubulin polymerisation, indicating their ability to interfere with the cytoskeletal structure [16]. Fluoroquinolone analogues of these hybrids induce cell cycle arrest in the G2/M phase and significantly upregulate DNA damage markers such as γH2AX, which is correlated with activation of DNA repair pathways and subsequent cell death [14,16]. Furthermore, many hybrids promote apoptosis, as evidenced by higher levels of pro-apoptotic proteins, including Bax, caspase-9, and caspase-3, along with a reduction in the anti-apoptotic protein Bcl-2 after treatment [12,15,16]. Furthermore, both review and experimental studies indicate that these heterocycles can modulate kinase signalling pathways and may act on additional molecular targets involved in cancer cell proliferation and survival [17,18].

Ciprofloxacin remains the main compound in studies on fluoroquinolone–triazole hybrids, despite the reported antiproliferative properties of other fluoroquinolones such as moxifloxacin and lomefloxacin [19,20,21]. Although ciprofloxacin is the most widely investigated fluoroquinolone scaffold in the anticancer hybrid design, structural diversification of other fluoroquinolone derivatives may provide additional opportunities for modulation of biological properties. Lomefloxacin differs from ciprofloxacin in the presence of an additional fluorine substituent at the C8 position and a modified piperazine moiety, which may influence electronic characteristics, lipophilicity, molecular interactions, and cellular distribution. Therefore, lomefloxacin represents an alternative scaffold for exploring the relationship between fluoroquinolone structure and antiproliferative activity. Previous studies have indicated that lomefloxacin and its derivatives can exhibit cytotoxic effects against cancer cells, suggesting that this scaffold could serve as a valuable starting point for the development of novel anticancer agents [22,23]. Considering the ability of fluoroquinolone derivatives to interfere with molecular processes involved in cancer cell proliferation, particularly through their reported interactions with DNA topoisomerases and induction of DNA damage, the incorporation of additional pharmacophoric fragments represents a promising strategy for enhancing their biological properties [24,25,26]. Among such structural modifications, the introduction of a 1,2,3-triazole moiety has attracted considerable attention due to the favourable physicochemical characteristics of this heterocycle and its ability to participate in hydrogen bonding and π–π interactions, which may influence molecular recognition and biological activity [15,27].

The C7 position of the fluoroquinolone scaffold, which commonly contains a piperazine substituent, is one of the most frequently modified regions because structural alterations at this site generally preserve the quinolone pharmacophore while allowing modulation of the physicochemical properties and biological activity. Functionalization of the N4 atom of the piperazine ring enables the introduction of additional pharmacophoric fragments without disrupting the structural features associated with the biological activity of the quinolone scaffold. Consequently, this position has become a preferred site for the design of fluoroquinolone hybrids with potentially enhanced anticancer potential [11]. The substituents attached to the triazole ring were selected to provide structural diversity and modulate steric and electronic characteristics of the designed molecules. Aromatic substituents containing electron-withdrawing groups, including halogen atoms and nitro functionality, were introduced to influence lipophilicity, molecular recognition, and potential interactions with biological targets. Such modifications enabled a preliminary evaluation of structure–activity relationships within the synthesised lomefloxacin–triazole series [28,29]. Previous studies have shown that variations in aromatic substituents of 1,2,3-triazole hybrids can affect their physicochemical properties and antiproliferative activity, highlighting the importance of substituent optimisation during the design of novel anticancer candidates. Therefore, we hypothesised that the combination of the lomefloxacin scaffold with a 1,2,3-triazole fragment could provide novel hybrid molecules with improved antiproliferative potential by integrating the biological properties of both structural motifs.

Based on these observations, the purpose of the present study was to expand the library of hybrid fluoroquinolone derivatives containing a 1,2,3-triazole moiety with potential antiproliferative activity. To this end, a series of triazole hybrids was designed and synthesised, and their anticancer potential was evaluated in vitro. We evaluated the effects of newly synthesised compounds on the proliferation of selected cancer cell lines and provided preliminary insights into the biological activities of these novel structures.

## 2. Materials and Methods

### 2.1. Materials

#### Cell Cultures

The study was carried out using human cell lines: amelanotic melanoma SK-MEL 28 and C32, triple negative breast cancer MDA-MB-231, hormone-dependent breast cancer MCF-7, as well as colorectal adenocarcinoma HT 29 and DLD-1, all obtained from the American Type Culture Collection (ATCC, Manassas, VA, USA). Cells were maintained under standard in vitro culture conditions, using DMEM (Sigma-Aldrich, St. Louis, MO, USA) as the base medium, while HT 29 cells were cultured in McCoy medium (Sigma-Aldrich, St. Louis, MO, USA), following supplier recommendations. All media were supplemented with 10% Foetal Bovine Serum (FBS, Sigma-Aldrich, St. Louis, MO, USA) and a mixture of antibiotics (penicillin/streptomycin) to minimise the risk of microbial contamination. Human dermal fibroblast (HDF) was obtained from Cell Applications, Inc. (San Diego, CA, USA) and cultured in dedicated fibroblast growth medium (Cell Applications, Inc. San Diego, CA, USA). Cultures were incubated in Binder CB 160 (BINDER GmbH, Steinheim, Germany) at 37 °C in a 5% CO_2_ atmosphere with controlled humidity. Cells remained in the logarithmic growth phase and passages were performed when cultures reached approximately 70–80% confluence, using standard procedures for detaching adherent cells.

### 2.2. Methods

#### 2.2.1. Molecular Docking

The appropriate three-dimensional structures of the docking ligands were generated using Gaussian 16 (Revision C-01). The lowest energy conformations are found using Density Functional Theory (DFT) with the B3LYP functional and the 6-311+G(d,p) basis set.

Complexes of topoisomerases I and II with camptothecin and etoposide as ligands were obtained from the Protein Data Bank (1T8I, 6ZY7, 7YQ8). The Hermes visualizer ver. 2026.1.1 in the GOLD Suite was used to further prepare receptors. All hydrogen atoms, including those necessary to define the correct ionisation and tautomeric states of residues such as Asp, Glu, and His, were added, and all water molecules and ligands were deleted for docking.

Genetic Optimisation for Ligand Docking (GOLD ver. 2026.1.1) was used for the docking studies. GOLD is an automated ligand docking programme that uses a genetic algorithm to explore the full range of ligand conformational flexibility with partial flexibility of the protein (flexibility of receptor hydrogens). The region of interest used for the GOLD docking was defined as all the protein residues within the 6 Å of the reference ligand. Default values of all other parameters were used and the complexes were submitted to the population size (100), the number of operations (100,000), the selection pressure (1.1), the number of islands (5); niche size (2); and the operator weights for migration (10), mutate (95), and crossover (95). Genetic Algorithm settings were used for all calculations and a set of 10 solutions was saved for each ligand. GOLD was used by a GoldScore fitness function. All results obtained were ranked according to their score value and presented in GOLD arbitrary units (a.u.). After calculations, only the ten highest-scoring poses were returned as the docking result for the ligand–cavity configuration.

#### 2.2.2. Molecular Dynamics Settings

Ligand (molecule **4**) and protein–DNA topologies were prepared separately using Topolbuild 1.3 software with GAFF2 force field parameters and the Gromacs [30] pdb2gmx programme using the AMBER99SB-ILDN force field parameters, respectively. Then, the entire system was integrated, placed in a cubic box, solvated using the 3-site TIP3P model and neutralised with Na^+^ and Cl^−^ ions. To ensure that the system had no steric clashes or inappropriate geometry (or solvent orientation), the whole structure was relaxed during the energy minimization (EM) stage to obtain a reasonable starting structure for molecular dynamics simulation (MDs). The final preprocessing step, called equilibration, was performed in two phases. In the first (referred to as isothermal-isochoric or canonical, NVT), the temperature of the system should reach a plateau at the desired value (typically 298 K) within the time of 100 ps. In the second one, the stabilisation of pressure was carried out under an NPT (or isothermal–isobaric) ensemble for 500 ps. In fact, the whole system equilibrated and plateaued fairly rapidly, remaining nearly flat for the rest of the run. Then, the well-equilibrated protein–DNA–ligand complex with the released restraints underwent 10 ns MDs calculations run on 12 CPU cores (since Gromacs supports the use of GPU accelerators) with Debian GNU/Linux 12 operating system.

#### 2.2.3. Synthesis of Lomefloxacin–Triazole Hybrids

The title lomefloxacin derivatives were obtained in a two-step process adapted from the procedure developed for ciprofloxacin [31].

In the first step, lomefloxacin hydrochloride (10 mM) and sodium bicarbonate (10 mM) were dissolved in DMF (5 mL), followed by the addition of an 80% solution of propargyl bromide in toluene (8.4 mM). The mixture was heated at 80 °C and stirred for 24 h. After completion of the reaction, the solution was basified with 1% NaOH and filtered, and then the filtrate was acidified with 10% HCl to pH 6.5. The resulting precipitate was filtered and the crude product was purified by column chromatography using an MeOH/DCM 1:10 mixture as eluent to obtain the pure propargyl derivative **1**.

The obtained product was used as a substrate in the next step, involving a Cu(I) ion-catalysed azide–alkyne cycloaddition. The propargyl derivative (0.5 mM) and the appropriate azide (1 mM) were dissolved in DMF (5 mL), followed by the addition of an extemporaneously prepared solution of sodium ascorbate (0.10 mM) and CuSO_4_·5H_2_O (0.05 mM). The reaction was carried out at 30 °C with constant stirring, and then the reaction mixture was poured onto water, the resulting precipitate was filtered and air-dried. Products **2**–**9** were purified by column chromatography, using MeOH/DCM mixtures in 0.5:10 proportions as eluent.

#### 2.2.4. Structural Analysis (NMR, HR MS, and HPLC) of the Derivatives

NMR spectra (^1^H, ^13^C, HSQC and HMBC) were recorded in a Bruker Ascend 600 DMSO-d6 solution (Bruker Corporation, Billerica, MA, USA) and calibrated for residual solvent signals. The values of the coupling constants are given in Hertz (Hz), and the resulting resonance peaks are described as follows: br s. (broad singlet), br d. (broad doublet), s (singlet), d (doublet), t (triplet), q (triplet of doublets), tt (triplet of triplets), and m (multiplet). The numbering system shown in Figure S1 in the Supplementary File (File S1) was used to describe the NMR spectra. All NMR experiments were conducted at room temperature and processed with TopSpin 3.7.0 software.

High-resolution mass spectrometric analyses (HR MS-ESI) were performed on a Bruker Impact II mass spectrometer equipped with an ESI source of ions.

The melting points were not corrected and measured with an IA 9300 melting point apparatus (Electrothermal).

Thin layer chromatography (TLC) was performed on 60 F254 silica gel plates (Merck, Darmstadt, Germany) using a 90/5 *v*/*v* methylene chloride methanol solution and the spots were visualised by UV light (254 nm). All new compounds were purified by column chromatography. Silica gel 60 (Merck, Darmstadt, Germany) was used as a solid phase and 90/5 *v*/*v* methylene chloride methanol solution as an eluent.

LC analysis was performed on a Dionex UltiMate 3000 kit (Thermo Fisher Scientific, Waltham, MA, USA). An Accucore RP-MS HPLC column (Thermo Fisher Scientific) was used with a length of 150 mm and a diameter of 2.1 mm. ACN/1% aqueous HCOOH 80/20 *v*/*v* solution was used as eluent. The determinations were made at a wavelength of 285 nm.

1-ethyl-6,8-difluoro-7-{3-methyl-[4-(1-(prop-2-yn-1-yl)piperazin-1-yl]}-4-oxo-1,4-dihydroquinoline-3-carboxylic acid (**1**)

Yield: 84%; mp 197–8 °C; HPLC purity 97.97%, *t*_R_ = 3.853 min; ^1^H NMR (600 MHz, DMSO-d_6_) δ [ppm]: 14.95 (bs, 1H, COOH), 8.96 (s, 1H, H-2), 7.85 (d, ^3^*J*_H-F_ = 11.7 Hz, 1H, H-5), 4.62–4.63 (m, 2H, H-12), 3.67–3.70 (m, 1H, H-15), 3.23 (s, 1H, -C≡CH), 3.03 (t, ^3^*J*_H-H_ = 10.4 Hz, 1H, H-18), 2.81 (d, ^3^*J*_H-H_ = 11.9 Hz, 1H, H-16), 2.72 (m, 1H, H-15) 2.56–2.66 (m, 2H, H-16 and H-19, 1.49 (t, ^3^*J*_H-H_ = 6.8 Hz, 3H, H-13), 1.06 (d, ^3^*J*_H-H_ = 6.2 Hz, 3H, H-20); ^13^C NMR (151 MHz, DMSO-d_6_) δ [ppm]: 175.9 (C-4), 166.0 (C-11), 154.0 (dd, ^1^*J*_C-F_ = 256.7 Hz, ^3^*J*_C-F_ = 6.3 Hz C-6), 151.6 (C-2), 146.2 (dd, ^1^*J*_C-F_ = 256.7 Hz, ^3^*J*_C-F_ = 6.3 Hz C-8), 134.0 (C-4a), 134.0 (d, ^2^*J*_C-F_, 15 Hz, C-7), 127.7 (C-8a), 120.7 (C-3), 107.4 (d, ^2^*J*_C-F_ = 12.3 Hz, C-5), 78.6 (-C≡CH), 76.6 (-C≡CH), 57.3 (C-18), 54.2 (C-19), 53.7 (C-12), 51.9 (C-15), 51.0 (C-16), 42.4 (-CH_2_-C≡CH), 16.4 (C-13); 16.3 (C-20); ^19^F NMR (565 MHz, DMSO-d_6_) δ [ppm]: −119.3 ppm (d, ^2^*J*_C-F_ = 10.7 Hz F-6), −129.8 ppm (d, ^4^*J*_F-F_ = 7.7 Hz, F-8); HRMS (ESI) m/z: C_20_H_22_F_2_N_3_O_3_ [M+H]^+^, Calcd. 390.1624; Found 390.1633.

1-ethyl-6,8-difluoro-7-{3-methyl-4-[(1-phenyl-1*H*-1,2,3-triazol-4-yl)methyl]piperazin-1-yl}-4-oxo-1,4-dihydroquinoline-3-carboxylic acid (**2**)

Yield: 78%; mp 211–15 °C; HPLC purity 97.26%, *t*_R_ = 3.387 min; ^1^H NMR (600 MHz, DMSO-d_6_) δ [ppm]: 14.90 (s, 1H, COOH), 8.89 (s, 1H, H-2), 8.77 (s, 1H, H-26), 7.92 (d, ^3^*J*_H-H_ = 7.7 Hz, 2H, H-28 and H-32), 7.81 (d, ^3^*J*_H-F_ = 11.7 Hz, 1H, H-5), 7.59 ppm (t, ^3^*J*_H-H_ = 7.7 Hz, 2H, H-29 and H-31), 7.48 (t, ^3^*J*_H-H_ = 7.4 Hz, 1H, H-30), 4.55 (s, 2H, H-12), 3.88–3.97 (m, 2H, H-15), 3.03 (t, ^3^*J*_H-H_ = 10.4 Hz, 1H, H-18), 2.87 (d, ^3^*J*_H-H_ = 11.9 Hz, 1H, H-16), 1.42 (t, ^3^*J*_H-H_ = 6.8 Hz, 3H, H-13), 1.21 (d, ^3^*J*_H-H_ = 6.1 Hz, 3H, H-20); ^13^C NMR (151 MHz, DMSO-d_6_) δ [ppm]: 176.0 (C-4), 166.0 (C-11), 154.9 (d, ^1^*J*_C-F_ = 247.5 Hz, C-6), 151.6 (C-2), 146.2 (d, ^1^*J*_C-F_ = 249.0 Hz, C-8), 144.0 (C-22), 137.2 (C-27), 134.1 (t, ^2^*J*_C-F_, 15 Hz, C-7), 130.3 (C-29 and C-31), 129.0 (C-30 and C-3), 127.7 (C-4a), 122.5 (C-26), 120.7 (C-8a), 120.3 (C-28 and C-32), 107.4 (t, ^2^*J*_C-F_, 15 Hz, C-5), 57.60 (C-12), 54.2 (C-21), 54.1 (C-18), 51.4 (C-19), 51.2 (C-16), 47.9 (C-15), 16.0 (C-20), 16.4 (C-13); ^19^F NMR (565 MHz, DMSO-d_6_) δ [ppm]: −119.4 ppm (s, F-6), −129.76 ppm (s, F-8); HRMS (ESI) m/z: C_26_H_27_F_2_N_6_O_3_ [M+H]^+^, Calcd. 509.2107; Found 509.2105.

1-ethyl-6,8-difluoro-7-{3-methyl-4-[(1-(4-chlorophenyl)-1*H*-1,2,3-triazol-4-yl)methyl]piperazin-1-yl}-4-oxo-1,4-dihydroquinoline-3-carboxylic acid (**3**)

Yield: 69%; mp 228–9 °C; HPLC purity 99.67%, *t*_R_ = 3.753 min; ^1^H NMR (600 MHz, DMSO-d_6_) δ 14.90 (bs, 1H, COOH), 8.89 (s, 1H, H-2), 8.80 (s, 1H, H-26), 7.98 (d, ^3^*J*_H-H_ = 8.3 Hz, 2H, H-29, H-31), 7.78 ppm (d, ^3^*J*_H-F_ = 11.8 Hz, 1H, H-5), 7.66 (d, ^3^*J*_H-H_ = 8.4 Hz, **2H**, H-28 and H-32), 4.55 (br q, ^3^*J*_H-H_ = 6.5 Hz, 2H, H-12), 3.87–3.96 (m, 2H, H-15), 3.01 (t, ^3^*J*_H-H_ = 10.2 Hz, 1H, H-18), 2.87 (d, ^3^*J*_H-H_ = 11.0 Hz, 1H, H-16), 1.40 (t, ^3^*J*_H-H_ = 6.5 Hz, 3H, H-13), 1.20 (d, ^3^*J*_H-H_ = 5.8 Hz, 3H, H-20); ^13^C NMR (151 MHz, DMSO-d_6_) δ: 175.9 (C-4), 166.0 (C-11), 154.9 (d, ^1^*J*_C-F_ = 249.2 Hz, C-6), 151.6 (C-2), 146.2 (d, ^1^*J*_C-F_ = 283.9 Hz, C-8), 144.2 (C-22), 135.9 (C-27), 134.1 (C-7), 133.1 (C-29 and C-31), 130.3 (C-30 and C-4a), 127.7 (C-8a), 122.6 (C-26), 120.7 (C-3), 120.3 (C-28 and C-32), 107.4 (d, ^2^*J*_C-F_, 10.0 Hz, C-5), 57.6 (C-18), 54.2 (C-19), 54.1 (C-12), 51.4 (C-15), 51.1 (C-21), 47.9 (C-16), 16.4 (C-13); 16.0 (C-20); ^19^F NMR (565 MHz, DMSO-d_6_) δ -119.3 (d, ^3^*J*_F-H_ = 9.6 Hz, F-6), -129.67 (d, ^4^*J*_F-F_ = 8.6 Hz F-8); HRMS (ESI) m/z: C_26_H_26_ClF_2_N_6_O_3_ [M+H]^+^, Calcd. 543.1718 (100%), 545.1689 (32%); Found 543.1714 (100%), 545.1682 (32%).

1-ethyl-6,8-difluoro-7-{3-methyl-4-[(1-(4-bromophenyl)-1*H*-1,2,3-triazol-4-yl)methyl]piperazin-1-yl}-4-oxo-1,4-dihydroquinoline-3-carboxylic acid (**4**)

Yield: 67%; mp 236–37 °C; HPLC purity 97.01%, *t*_R_ = 3.807 min; ^1^H NMR (600 MHz, DMSO-d_6_) δ 14.92 (bs, **1H**, COOH), 8.91 (s, **1H**, H-2), 8.81 (s, **1H**, H-26), 7.92 (d, ^3^*J*_H-H_ = 8.3 Hz, **2H**, H-29, H-31), 7.79–7.81 ppm (m, **3H**, H-5, H-28 and H-32), 4.55 (br q, ^3^*J*_H-H_ = 6.5 Hz, **2H**, H-12), 3.87–3.97 (m, **2H**, H-15), 3.02 (t, ^3^*J*_H-H_ = 10.2 Hz, **1H**, H-18), 2.87 (d, ^3^*J*_H-H_ = 11.0 Hz, **1H**, H-16), 1.41 (t, ^3^*J*_H-H_ = 6.5 Hz, **3H**, H-13), 1.20 (d, ^3^*J*_H-H_ = 5.8 Hz, **3H**, H-20); ^13^C NMR (151 MHz, DMSO-d_6_) δ: 175.9 (C-4), 166.0 (C-11), 154.9 (d, ^1^*J*_C-F_ = 249.2 Hz, C-6), 151.6 (C-2), 146.2 (d, ^1^*J*_C-F_ = 283.9 Hz, C-8), 144.2 (C-22), 135.9 (C-27), 134.1 (C-7), 133.1 (C-29 and C-31), 130.3 (C-30 and C-4a), 127.7 (C-8a), 122.6 (C-26), 120.7 (C-3), 120.3 (C-28 and C-32), 107.4 (d, ^2^*J*_C-F_, 10.0 Hz, C-5), 57.6 (C-18), 54.2 (C-19), 54.1 (C-12), 51.4 (C-15), 51.1 (C-21), 47.9 (C-16), 16.4 (C-13); 16.0 (C-20); ^19^F NMR (565 MHz, DMSO-d_6_) δ −119.3 (d, ^3^*J*_F-H_ = 9.6 Hz, F-6), −129.67 (d, ^4^*J*_F-F_ = 8.6 Hz F-8); HRMS (ESI) m/z: C_26_H_26_BrF_2_N_6_O_3_ [M+H]^+^, Calcd. 587.1213 (100%), 589.1192 (97.3%); Found 587.1214 (100%), 589.1197 (96.7%).

1-ethyl-6,8-difluoro-7-{3-methyl-4-[(1-(4-nitrophenyl)-1*H*-1,2,3-triazol-4-yl)methyl]piperazin-1-yl}-4-oxo-1,4-dihydroquinoline-3-carboxylic acid (**5**)

Yield: 66%; mp 231–34 °C; HPLC purity 95.36%, *t*_R_ = 3.540 min; ^1^H NMR (600 MHz, DMSO-d_6_) δ 14.92 (bs, **1H**, COOH), 9.01 (s, **1H**, H-2), 8.92 (s, **1H**, H-26), 8.47 (d, ^3^*J*_H-H_ = 9.1 Hz, **2H**, H-29, H-31), 8.27 (d, ^3^*J*_H-H_ = 9.1 Hz, **2H**, H-28, H-32), 7.83 (d, ^3^*J*_F-H_ = 11.8 Hz, **1H**, H-5), 4.56 (bs, **2H**, H-12), 3.90–4.00 (m, **2H**, H-15), 3.03 (t, ^3^*J*_H-H_ = 10.4 Hz, **1H**, H-18), 2.88 (d, ^3^*J*_H-H_ = 11.0 Hz, **1H**, H-16), 1.41 (t, ^3^*J*_H-H_ = 6.7 Hz, **3H**, H-13), 1.21 (d, ^3^*J*_H-H_ = 6.0 Hz, **3H**, H-20); ^13^C NMR (151 MHz, DMSO-d_6_) δ: 176.0 (C-4), 166.0 (C-11), 154.9 (d, ^1^*J*_C-F_ = 254.3 Hz, C-6), 151.7 (C-2), 147.0 (C-30), 146.2 (d, ^1^*J*_C-F_ = 238.1 Hz, C-8), 144.8 (C-22), 141.4 (C-27), 134.0 (C-7), 127.7 (C-8a), 126.0 (C-29 and C-31), 123.0 (C-26), 120.9 (C-30 and C-4a), 120.8 (C-28 and C-32), 120.7 (C-3), 107.4 (d, ^2^*J*_C-F_, 14.3 Hz, C-5), 57.6 (C-18), 54.2 (C-19), 54.1 (C-12), 51.4 (C-15), 51.1 (C-21), 47.9 (C-16), 16.4 (C-13); 16.0 (C-20); ^19^F NMR (565 MHz, DMSO-d_6_) δ −121.7 (d, ^3^*J*_F-H_ = 8.6 Hz, F-6), -130.4 (d, ^4^*J*_F-F_ = 8.0 Hz F-8); HRMS (ESI) m/z: C_26_H_26_F_2_N_7_O_3_ [M+H]^+^, Calcd. 554.1958; Found 554.1969.

1-ethyl-6,8-difluoro-7-{3-methyl-4-[(1-benzyl-1*H*-1,2,3-triazol-4-yl)methyl]piperazin-1-yl}-4-oxo-1,4-dihydroquinoline-3-carboxylic acid (**6**)

Yield: 73%; mp 206–207 °C; HPLC purity 98.41%, *t*_R_ = 3.357 min; ^1^H NMR (600 MHz, DMSO-d_6_) δ 14.92 (bs, **1H**, COOH), 8.91 (s, **1H**, H-2), 8.11 (s, **1H**, H-26), 7.82 (d, ^3^*J*_H-F_ = 11.9 Hz, **1H**, H-5), 7.28–7.40 (m, **5H**, H-28, H-29, H-30, H-31 and H-32), 5.59 (s, **2H**, H-27), 4.56 (br q, ^3^*J*_H-H_ = 6.5 Hz, **2H**, H-12), 3.80–3.87 (m, **2H**, H-15), 3.00–3.28 (m, **2H**, H-21), 2.98 (d, ^3^*J*_H-H_ = 9.2 Hz, **1H**, H-18), 2.80 (d, ^3^*J*_H-H_ = 10.1 Hz, **1H**, H-16), 2.4 (m, 1**H**, H-18). 1.41 (t, ^3^*J*_H-H_ = 6.5 Hz, **3H**, H-13), 1.15 (d, ^3^*J*_H-H_ = 5.8 Hz, **3H**, H-20); ^13^C NMR (151 MHz, DMSO-d_6_) δ: 176.0 (C-4), 166.1 (C-11), 154.9 (d, ^1^*J*_C-F_ = 244.2 Hz, C-6), 151.6 (C-2), 146.2 (d, ^1^*J*_C-F_ = 250.9 Hz, C-8), 143.0 (C-22), 136.7 (C-27), 134.0 (C-7), 134.0 (C-29), 133.9 (C-29), 128.5 (C-31), 127.7 (C-30 and C-4a), 127.7 (C-8a), 124.6 (C-26), 120.7 (C-3), 120.6 (C-28 and C-32), 107.4 (d, ^2^*J*_C-F_, 10.0 Hz, C-5), 57.6 (C-18), 54.2 (C-19), 53.1 (C-27), 51.4 (C-12), 51.1 (C-15), 51.1 (C-21), 47.9 (C-16), 16.4 (C-13); 16.0 (C-20); ^19^F NMR (565 MHz, DMSO-d_6_) δ −119.3 (d, ^3^*J*_F-H_ = 11.0 Hz, F-6), −129.7 (d, ^4^*J*_F-F_ = 10.2 Hz F-8). HRMS (ESI) m/z: C_27_H_29_F_2_N_6_O_3_ [M+H]^+^, Calcd. 523.2264; Found 523.2266.

1-ethyl-6,8-difluoro-7-{3-methyl-4-[[1-(4-chlorobenzyl)-1*H*-1,2,3-triazol-4-yl]methyl]piperazin-1-yl}-4-oxo-1,4-dihydroquinoline-3-carboxylic acid (**7**)

Yield: 72%; mp 214–215 °C; HPLC purity 98.76%, *t*_R_ = 3.553 min; ^1^H NMR (600 MHz, DMSO-d_6_) δ 14.92 (bs, **1H**, COOH), 8.91 (s, **1H**, H-2), 8.11 (s, **1H**, H-26), 7.82 (d, ^3^*J*_H-F_ = 11.6 Hz, **1H**, H-5), 7.44 (d, ^3^*J*_H-H_ = 7.0 Hz, **2H**, H-29, H-31), 7.32 (d, ^3^*J*_H-H_ = 7.0 Hz, **2H**, H-28, H-32), 5.60 (s, **2H**, H-27), 4.56 (br q, ^3^*J*_H-H_ = 6.5 Hz, **2H**, H-12), 3.79–3.86 (m, **2H**, H-15), 2.98 (d, ^3^*J*_H-H_ = 9.2 Hz, **1H**, H-18), 2.80 (d, ^3^*J*_H-H_ = 10.1 Hz, **1H**, H-16), 2.4 (bs, **2H**, H-16 and H-18). 1.40 (t, ^3^*J*_H-H_ = 6.5 Hz, **3H**, H-13), 1.15 (d, ^3^*J*_H-H_ = 5.8 Hz, **3H**, H-20); ^13^C NMR (151 MHz, DMSO-d_6_) δ: 176.0 (C-4), 166.0 (C-11), 154.9 (d, ^1^*J*_C-F_ = 244.2 Hz, C-6), 151.6 (C-2), 146.2 (d, ^1^*J*_C-F_ = 250.9 Hz, C-8), 143.0 (C-22), 135.7 (C-27), 134.0 (C-7), 130.2 (C-29 and C-31), 129.2 (C-30 and C-4a), 127.7 (C-8a), 124.6 (C-26), 120.7 (C-3), 120.6 (C-28 and C-32), 107.4 (d, ^2^*J*_C-F_, 10.0 Hz, C-5), 57.6 (C-18), 54.2 (C-19), 52.4 (C-27), 51.4 (C-12), 51.1 (C-15), 51.1 (C-21), 47.9 (C-16), 16.4 (C-13); 16.0 (C-20); ^19^F NMR (565 MHz, DMSO-d_6_) δ -119.3 (d, ^3^*J*_F-H_ = 8.9 Hz, F-6), -129.7 (d, ^4^*J*_F-F_ = 7.7 Hz F-8); HRMS (ESI) m/z: C_27_H_28_ClF_2_N_6_O_3_ [M+H]^+^, Calcd. 557.1874 (100%), 559.1845 (32%); Found 557.1872 (100%), 559.1845 (32.7%).

1-ethyl-6,8-difluoro-7-{3-methyl-4-[[1-(4-bromobenzyl)-1*H*-1,2,3-triazol-4-yl]methyl]piperazin-1-yl}-4-oxo-1,4-dihydroquinoline-3-carboxylic acid (**8**)

Yield: 60%; mp 220–221 °C; HPLC purity 96.52%, *t*_R_ = 3.640 min; ^1^H NMR (600 MHz, DMSO-d_6_) δ 14.93 (bs, **1H**, COOH), 8.92 (s, **1H**, H-2), 8.12 (s, **1H**, H-26), 7.83 (d, ^3^*J*_H-F_ = 11.7 Hz, **1H**, H-5), 7.57 (d, ^3^*J*_H-H_ = 8.2 Hz, **2H**, H-29, H-31), 7.25 (d, ^3^*J*_H-H_ = 8.2 Hz, **2H**, H-28, H-32), 5.58 (s, **2H**, H-27), 4.57 (s, **2H**, H-12), 3.79–3.87 (m, **2H**, H-15), 3.29 (bs, 2H, H-12), 3.00 (t, ^3^*J*_H-H_ = 10.0 Hz, **1H**, H-18), 2.80 (d, ^3^*J*_H-H_ = 9.8 Hz, **1H**, H-16), 2.41 (bs, **2H**, H-16 and H-18), 1.42 (t, ^3^*J*_H-H_ = 6.6 Hz, **3H**, H-13), 1.15 (d, ^3^*J*_H-H_ = 5.8 Hz, **3H**, H-20); ^13^C NMR (151 MHz, DMSO-d_6_) δ: 176.0 (C-4), 166.1 (C-11), 154.9 (d, ^1^*J*_C-F_ = 249.2 Hz, C-6), 151.6 (C-2), 146.2 (d, ^1^*J*_C-F_ = 239.5 Hz, C-8), 143.0 (C-22), 136.1 (C-27), 134.1 (C-30), 134.0 (C-29 and C-31), 130.5 (C-7 and C-4a), 127.7 (C-8a), 127.6 (C-26), 124.7 (C-3), 121.8 (C-28 and C-32), 107.4 (d, ^2^*J*_C-F_, 18.1 Hz, C-5), 57.6 (C-18), 54.18 (C-19), 52.4 (C-27), 51.4 (C-12), 51.1 (C-15), 51.1 (C-21), 47.9 (C-16), 16.4 (C-13); 16.0 (C-20); ^19^F NMR (565 MHz, DMSO-d_6_) δ −119.3 (d, ^3^*J*_F-H_ = 10.7 Hz, F-6), −129.7 (d, ^4^*J*_F-F_ = 10.4 Hz F-8). HRMS (ESI) m/z: C_27_H_28_BrF_2_N_6_O_3_ [M+H]^+^, Calcd. 601.1369 (100%), 603.1349 (97.3%); Found 601.1375 (100%), 603.1359 (96.3%).

1-ethyl-6,8-difluoro-7-{3-methyl-4-[[1-(4-nitrobenzyl)-1*H*-1,2,3-triazol-4-yl]methyl]piperazin-1-yl}-4-oxo-1,4-dihydroquinoline-3-carboxylic acid (**9**)

Yield: 70%; mp 240–241 °C; HPLC purity 95.81%, *t*_R_ = 3.283 min; ^1^H NMR (600 MHz, DMSO-d_6_) δ 14.86 (bs, **1H**, COOH), 8.92 (s, **1H**, H-2), 8.32 (s, **1H**, H-26), 8.23 (d, ^3^*J*_H-H_ = 8.5 Hz, **2H**, H-29, H-31), 7.83 (d, ^3^*J*_H-F_ = 12.7 Hz, **1H**, H-5), 7.53 (d, ^3^*J*_H-H_ = 8.4 Hz, **2H**, H-28, H-32), 5.83 (s, **2H**, H-27), 4.55–4.57 (m, **2H**, H-12), 3.93–3.98 (m, **2H**, H-15), 3.02 (d, ^3^*J*_H-H_ = 9.2 Hz, **1H**, H-18), 2.89 (t, ^3^*J*_H-H_ = 10.1 Hz, **1H**, H-16), 1.41 (t, ^3^*J*_H-H_ = 6.5 Hz, **3H**, H-13), 1.23 (d, ^3^*J*_H-H_ = 5.8 Hz, **3H**, H-20); ^13^C NMR (151 MHz, DMSO-d_6_) δ: 175.9 (C-4), 165.9 (C-11), 154.9 (d, ^1^*J*_C-F_ = 243.5 Hz, C-6), 151.7 (C-2), 146.2 (d, ^1^*J*_C-F_ = 247.5 Hz, C-8), 143.9 (C-22), 129.7 (C-27 and C-30), 129.4 (C-7), 129.3 (C-4a, C-29 and C-31), 127.7 (C-8a), 127.6 (C-26 and C-3), 124.4 (C-28 and C-32), 107.4 (d, ^2^*J*_C-F_, 18.1 Hz, C-5), 57.6 (C-18), 55.6 (C-19), 54.3 (C-27), 51.4 (C-12), 51.1 (C-15), 51.1 (C-21), 47.9 (C-16), 16.4 (C-13); 16.3 (C-20); ^19^F NMR (565 MHz, DMSO-d_6_) δ −119.3 (d, ^3^*J*_F-H_ = 9.0 Hz, F-6), −129.7 (d, ^4^*J*_F-F_ = 8.0 Hz F-8); HRMS (ESI) m/z: C_27_H_28_F_2_N_7_O_3_ [M+H]^+^, Calcd. 568.2114; Found 568.2107.

#### 2.2.5. Cell Viability Assay

Cell viability of cell lines in the presence of derivatives **1**–**9** was evaluated using the WST-1 reagent (Roche, Mannheim, Germany), which allows evaluation of cellular proliferation. The cells were seeded in 96-well plates at a density of 3 $\times 10^{3}$ cells per well and incubated for 24 h. Subsequently, the culture medium was replaced with solutions of compounds **1**–**9** or lomefloxacin at concentrations of 10, 25, 50, 75 and 100 µM, and the cells were incubated for an additional 72 h. The compounds were dissolved in DMSO, and its final concentration in the culture medium did not exceed 1% (*v*/*v*). Cells treated with 1% (*v*/*v*) DMSO without the tested compounds served as the vehicle control. Camptothecin was tested at concentrations of 0.05, 0.1, 0.5, 1 and 10 µM. The WST-1 reagent was added 2 h before the end of incubation and the absorbance was measured using the Infinite 200 Pro (TECAN, Mannedorf, Switzerland). The mean of the control wells was set at 100% and the dose–response curves along with the half-maximal inhibitory concentration (IC50) were determined using GraphPad Prism 10 software.

#### 2.2.6. Statistical Analysis

Statistical evaluation of the WST-1 results was performed using GraphPad Prism 10 (GraphPad Software, San Diego, CA, USA). In all experiments, the data are presented as mean ± standard deviation (SD) of at least three independent experiments (biological replicates, *n* = 3), each carried out in triplicate (technical replicates). The normality of the data distribution was assessed using the Shapiro–Wilk test. Differences between groups were analysed by one-way ANOVA, followed by Dunnett’s post hoc test. Each treatment group was compared with the corresponding untreated control group. IC50 values were determined by nonlinear regression analysis of concentration–response curves using a variable-slope model. Differences were deemed statistically significant when *p* < 0.05. The selectivity index (SI) was calculated as the ratio of the IC50 value obtained for normal human dermal fibroblasts (HDFs) to the corresponding IC50 value determined for each cancer cell line.

## 3. Results and Discussion

### 3.1. In Silico Evaluation of Potential Interactions of Lomefloxacin–Triazole Hybrids with Topo I, Topo IIα and Topo IIβ

**Figure 1:**
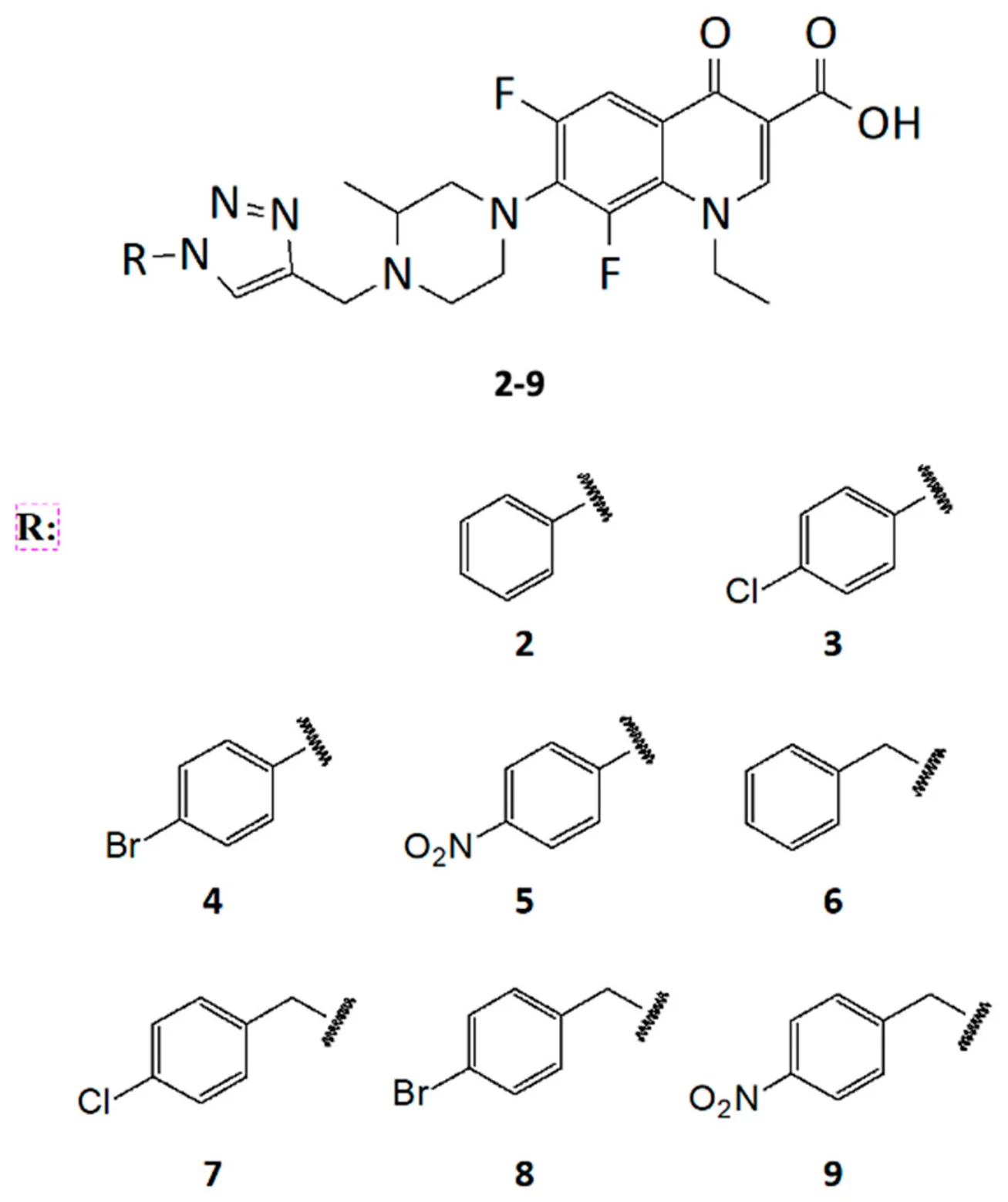
Molecular structures of the new lomefloxacin derivatives.

Structure–activity relationship studies have shown that structural modifications within the fluoroquinolone scaffold, particularly at the C7 position, may influence the physicochemical properties and biological activity of the resulting derivatives. Such modifications can affect interactions with eukaryotic molecular targets, including topoisomerases [11]. Therefore, molecular docking studies represent a valuable approach for preliminary evaluation of potential interactions between newly designed derivatives and selected biological targets. The aim of this study was to investigate the potential of novel lomefloxacin derivatives as potential binders of eukaryotic topoisomerases Topo I, Topo IIα and Topo IIβ among new lomefloxacin derivatives. Based on the designed lomefloxacin–triazole hybrid library, eight derivatives were selected for synthesis and further biological evaluation (Figure 1) using GOLD software ver. 2026.1.1. Compound **1** was used as the parent scaffold for the preparation of derivatives **2**–**9**.

The selected enzymes are targets for well-known anticancer agents used in cancer therapy. In this study, complexes of topoisomerase I with camptothecin and topoisomerase II with etoposide were used. These compounds, as well as lomefloxacin, possess structurally distinct but functionally analogous planar aromatic cores, allowing interactions with DNA and stabilisation of the DNA-topoisomerase cleavage complex.

**Figure 2:**
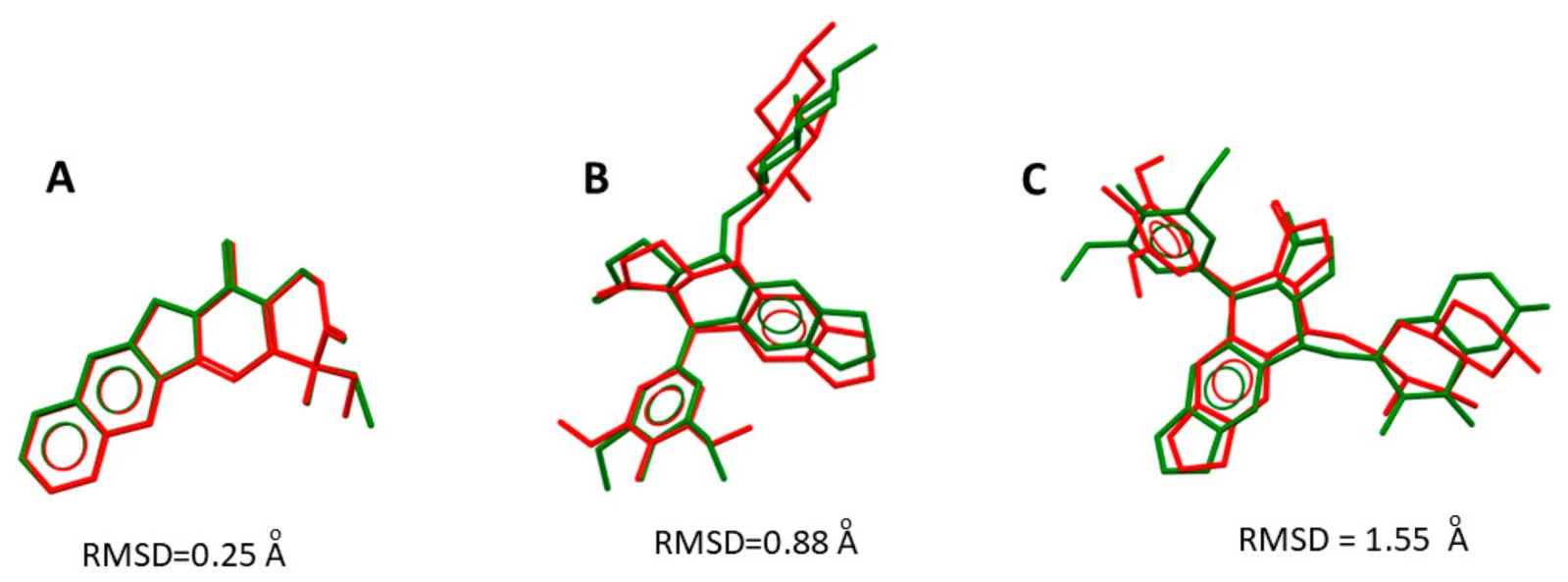
Value of the root mean square deviation between crystallised structure (green) and the docking pose (red) or camptothecin in complex with topoisomerase I (**A**), etoposide in complex with topoisomerase IIα (**B**), and etoposide in complex with topoisomerase IIβ (**C**).

In the first stage of the in silico studies, the accuracy of the GoldScore protocol in the GOLD software was verified. To this end, native inhibitors—camptothecin and etoposide—were redocked at the binding sites of their respective proteins, and the resulting postdocking conformations were compared with those found in native complexes. A root mean square deviation (RMSD) value of less than 2 Å for the ligand is generally considered to indicate an accurate prediction. The RMSD values between the crystal structures and the conformations obtained from the control binding were 0.25 Å, 0.88 Å, and 1.55 Å for topoisomerases I, IIα, and IIβ, respectively, demonstrating the high accuracy of the docking simulations performed using the GoldScore protocol (Figure 2). Consequently, the GoldScore protocol was deemed suitable for docking the compounds under study to the selected molecular targets.

The reference ligands and the lomefloxacin derivatives **2**–**9** were ranked using GOLD, as shown in Table 1. Higher scores correspond to stronger binding affinity and the most probable ligand–protein interactions. The results obtained are presented in the arbitrary units [a.u.] used by the GOLD programme. Subsequently, to compare and validate the docking results, they were converted into ΔG values [kcal/mol]. This conversion was based on data from publication [32], in which the authors compared experimentally determined binding energy values (ΔG [kJ/mol]) for a large library of complexes with the a.u. values obtained by redocking ligands to the tested molecular targets, resulting in the following correlation equation:

ΔG = (−0.4502 × GoldScore) − 9.4891 [kJ/mol]

Finally, the values obtained were converted into units of kilocalories per mole.

When comparing the docking scores obtained for all complexes of the new lomefloxacin derivatives with the selected molecular targets, it can be concluded that compounds **2**–**9** demonstrated favourable predicted interactions with the investigated topoisomerases, with docking scores comparable to those calculated for the reference ligands. The lowest ΔG values, and consequently the highest binding affinity to topoisomerase I were observed for compound **5**. Compared to the reference ligand etoposide, most derivatives **2**–**9** exhibited slightly less favourable predicted interactions with the investigated topoisomerase II isoforms. However, among the structures analysed, compound **7** for Topo IIα and derivatives **4** and **8** for Topo IIβ exhibit a binding affinity more similar to those observed for the reference ligand.

**Figure 3:**
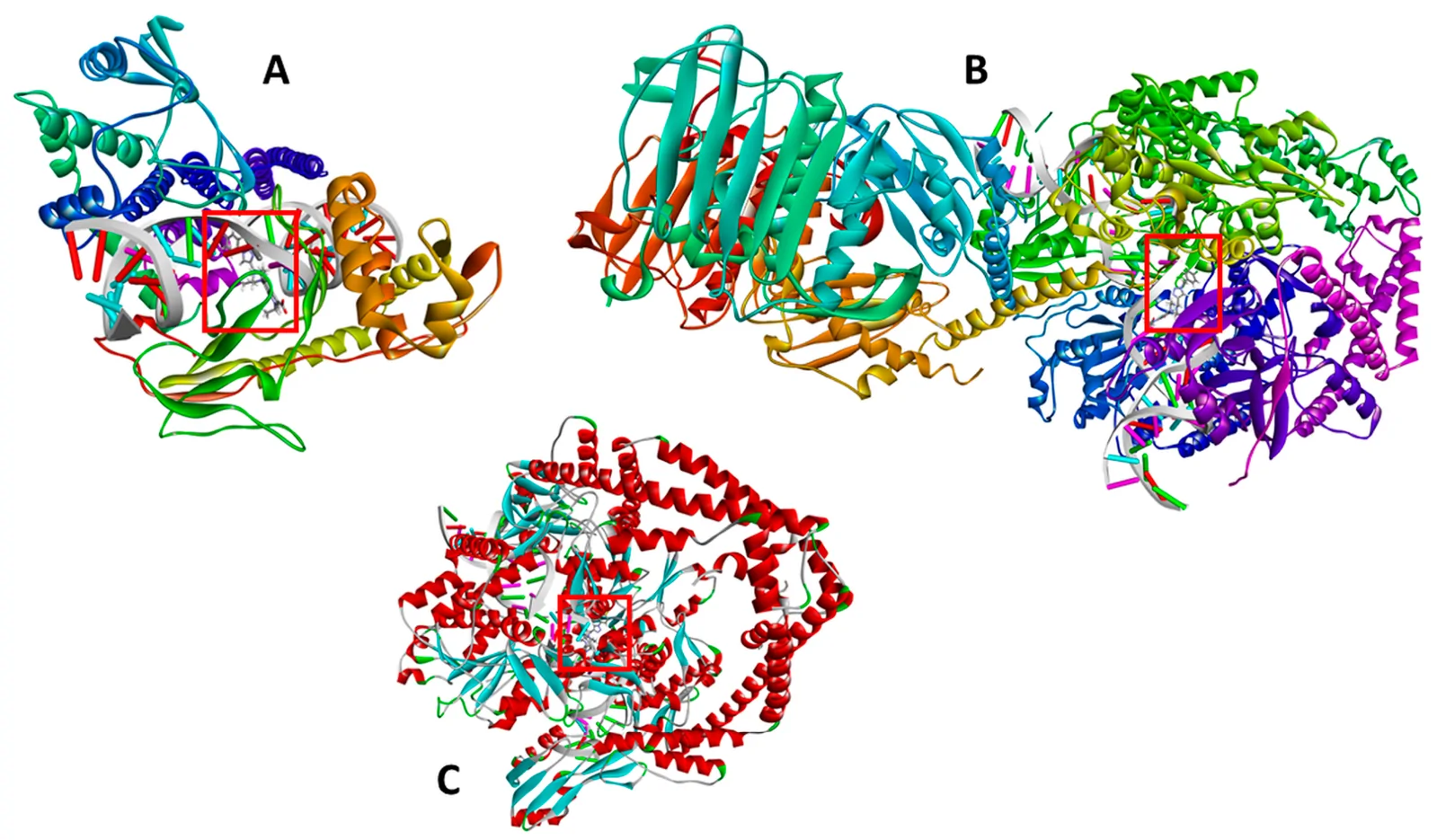
Binding mode of compound **5** evaluated to topoisomerase I (**A**), binding mode of compound **7** evaluated to topoisomerase IIα (**B**), and binding mode of compound **4** evaluated to topoisomerase IIβ (**C**) (ligand binding site is marked in red).

**Figure 4:**
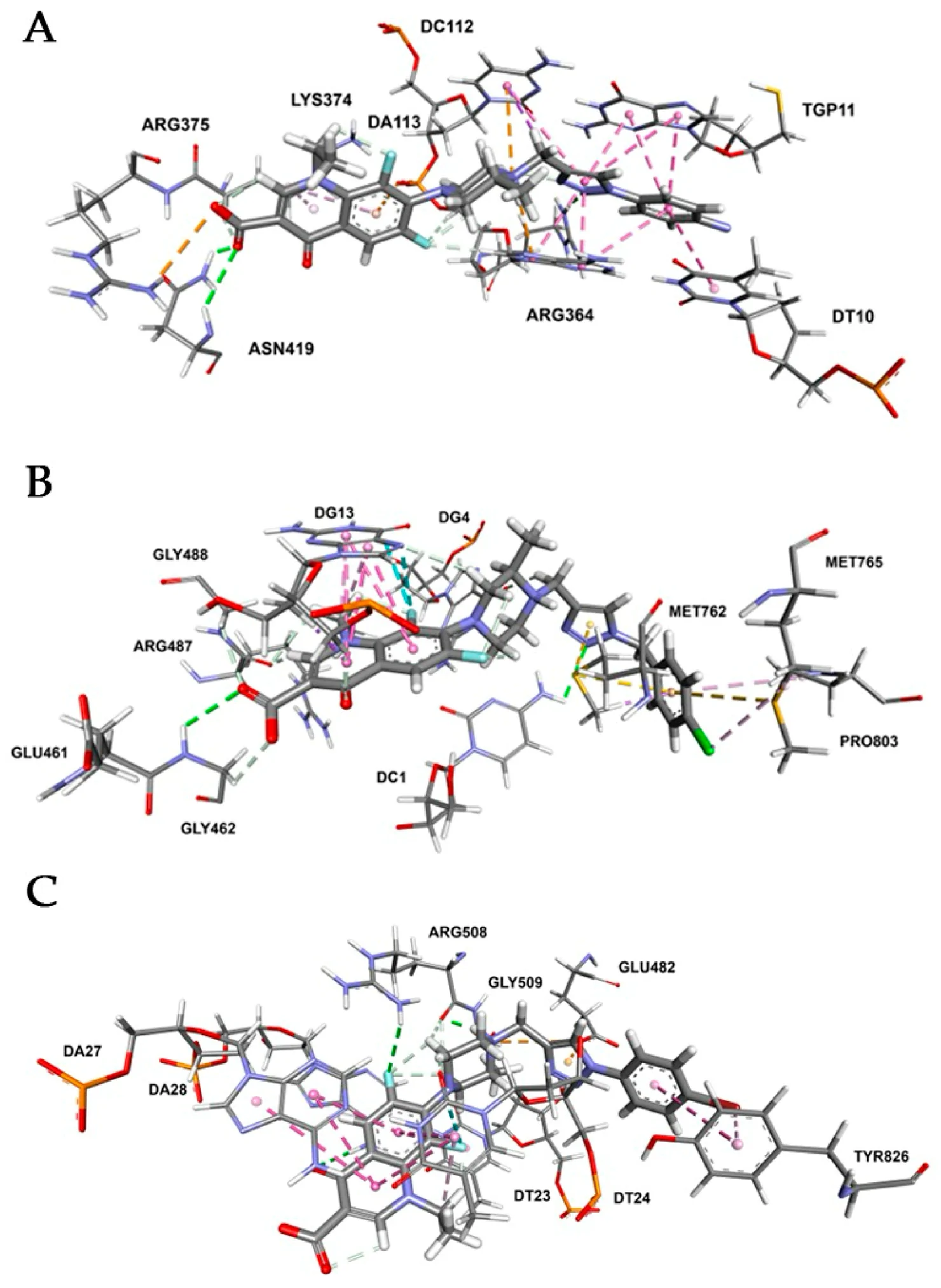
Three-dimensional interaction poses of compound **5** within active site of Topoisomerase I (**A**), 3D interaction poses of compound **7** within active site of Topoisomerase IIα (**B**), and 3D interaction poses of compound **4** within active site of Topoisomerase IIβ (**C**).

Detailed analysis of the interactions between ligand 5 and the amino acids of the topoisomerase I binding site indicates that two hydrogen bonds occur in this complex: between ARG364 and the nitrogen atom of the 1,4-disubstituted triazole system (2.19 Å), and between ASN419 and the oxygen atom of the carboxylate group (2.95 Å) of the lomefloxacin system (Figure 3 and Figure 4). ARG375 generates electrostatic interactions (attractive charge) with the negatively charged carboxylate group, while the nucleotide DC112 interacts through a π-cation with the ammonium cation of the piperazine system. The DC113 nucleotide interacts in a manner similar to that of the ammonium group. It is important to note that the complex is stabilised by numerous π–π stacking interactions that involve all nucleotides DT10, TGP11, DA112, and DA113.

Analysis of the interactions of ligand **7** with amino acids and nucleotides at the active site indicates that **7** interacts in a manner like compound **5**, forming hydrogen bonds via the oxygen atom of the carboxylate group (3.01 Å) and the nitrogen atom of the 1,4-disubstituted triazole system (2.86 Å). The carboxylate group interacts with GLY462, the triazole, with the DC1 nucleotide. MET765 forms significant π-sulphur interactions, while MET762 interacts with the *p*-chlorophenyl substituent through π-sigma interactions. DG13 significantly stabilises the complex through numerous interactions of various natures: carbon hydrogen bonding, π-sigma, π-alkyl, π–π stacking, and halogen. Derivative **4** forms four hydrogen bonds at the topoisomerase IIβ binding site. Two of these bonds are formed by ARG508. One acceptor is the fluorine atom at position 6 of the quinolone system (2.89 Å), while the other is formed by the ammonium group of the piperazine ring (1.97 Å). The nucleotide DA28 forms a hydrogen bond with the carbonyl oxygen atom of the quinolone system (2.78 Å), while DT23 forms a hydrogen bond with the nitrogen atom of the triazole ring (3.03 Å). The complex is stabilised by the electrostatic interactions of GLU428 (attractive charge and π-cation). Additionally, DA28 and DT24 form halogen bonds with F-6 and F-8 of the quinolone system, respectively. Furthermore, the complex of compound 4 with topoisomerase IIβ shows numerous π–π stacking interactions with the phenyl substituent and the quinolone ring, the formation of which involves TYR826, DT23, DA27 and DA28.

**Figure 5:**
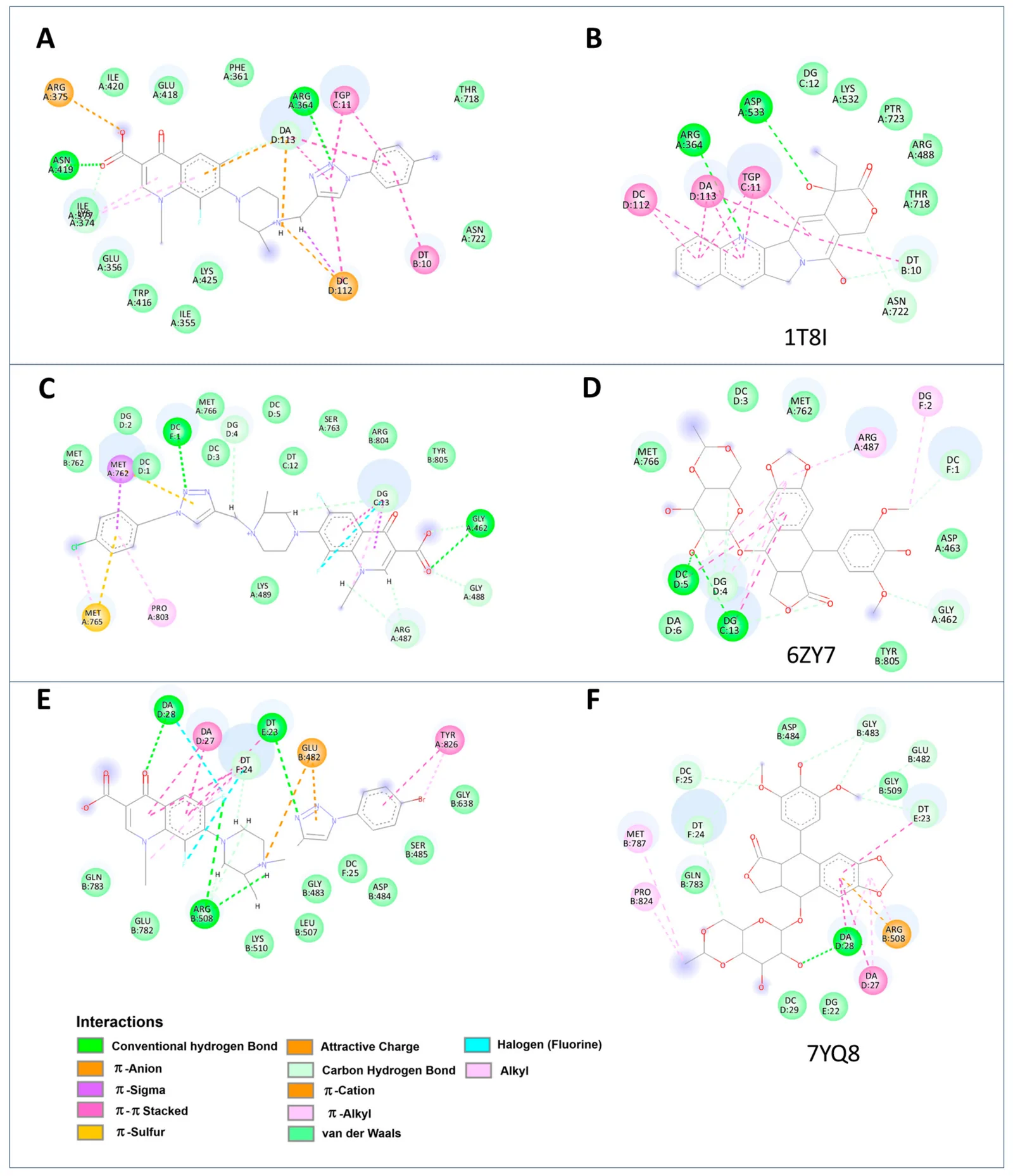
Two-dimensional view of the type of interaction of compound **5** with surrounding amino acids of Topoisomerase I (**A**), camptothecin with surrounding amino acids of Topoisomerase I (**B**), compound **7** with surrounding amino acids of Topoisomerase IIα (**C**), etoposide with surrounding amino acids of Topoisomerase IIα (**D**), compound **4** with surrounding amino acids of Topoisomerase IIβ (**E**) and etoposide with surrounding amino acids of Topoisomerase IIβ (**F**).

The in silico results obtained indicate that the 1,2,3-triazole system introduced into the lomefloxacin molecule, with an aromatic substituent at position 1, can positively influence the stability of the complex of the new derivatives with topoisomerase compared to camptothecin and etoposide (Figure 5). However, these findings provide only preliminary insights into potential molecular interactions and require further experimental validation.

**Figure 6:**
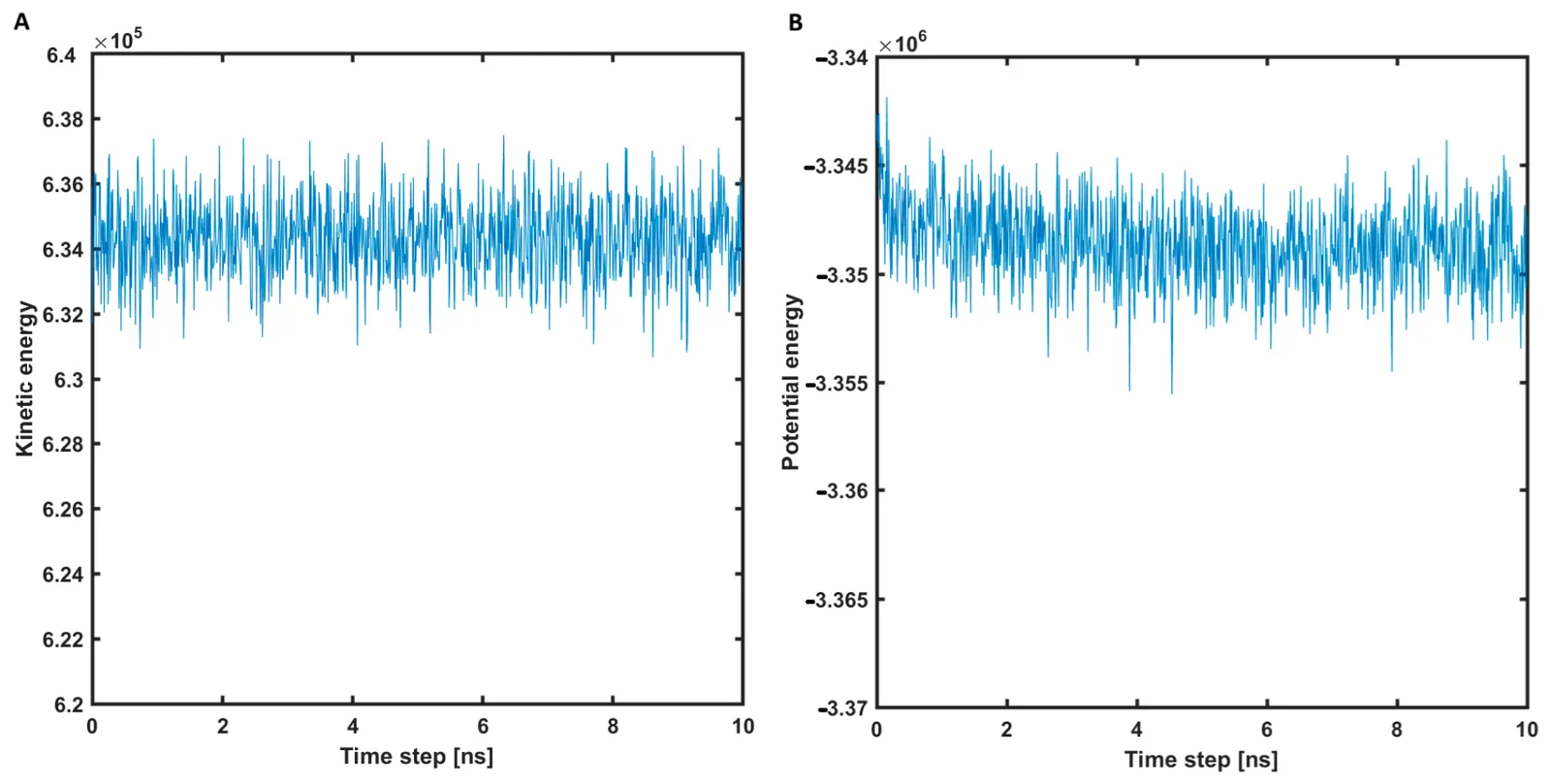
Kinetic (**A**) and potential (**B**) energy variations in the MD simulations of Topo IIβ and molecule **4**.

In the subsequent stage of the in silico studies, molecular dynamics (MD) calculations were performed for compound 4 and Topo IIβ. The interactions between the ligand and target depend on the pose (conformation and relative orientation) of both the host and guest molecules; therefore, it is advisable to perform the molecular docking study. In this context, the MD can offer valuable insights into the stability of the liganded (holo) protein system. Due to time and resource constraints, the structure-based MDs study of the liganded system was performed over a 10 ns simulation time. Both protein–DNA complex and compound **4** were corrected using the DockPrep module in Chimaera software [33]. The relative constant fluctuations in the kinetic/potential energy of the system during MDs are illustrated in Figure 6.

**Figure 7:**
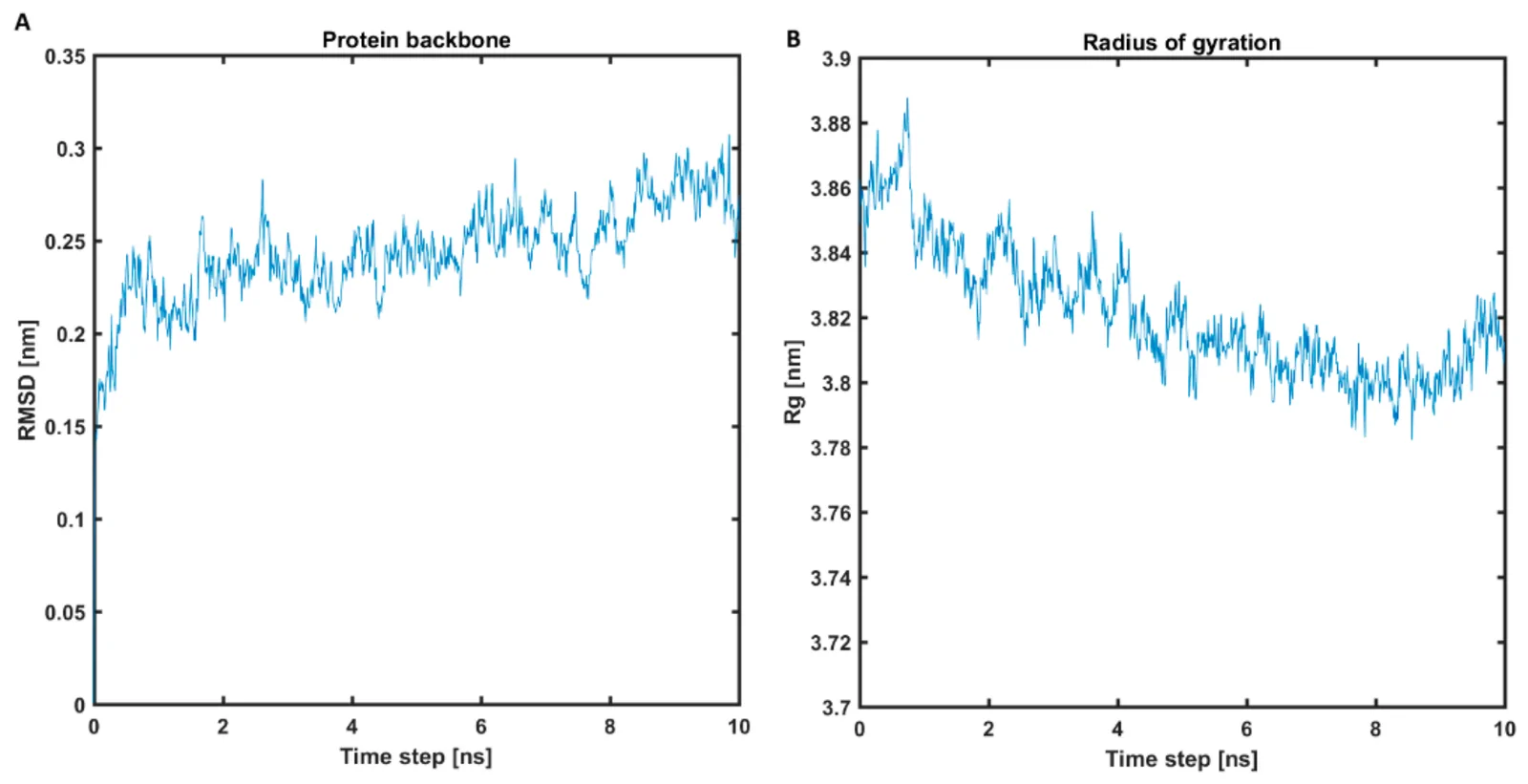
Fluctuations in the root mean square deviations for the protein backbone atoms (**A**) and the radius of the gyration variations (**B**) in the MD simulations of Topo IIβ and molecule **4**.

To evaluate the convergence of the simulation and/or determine the stability of the protein–DNA complex, the variations in the root mean square deviation (RMSD) metric (calculated for protein backbone atoms) are illustrated in Figure 7A. Furthermore, a rough measure of protein compactness over the 10 ns MDs called radius of gyration (R_g_) is shown in Figure 7B.

**Figure 8:**
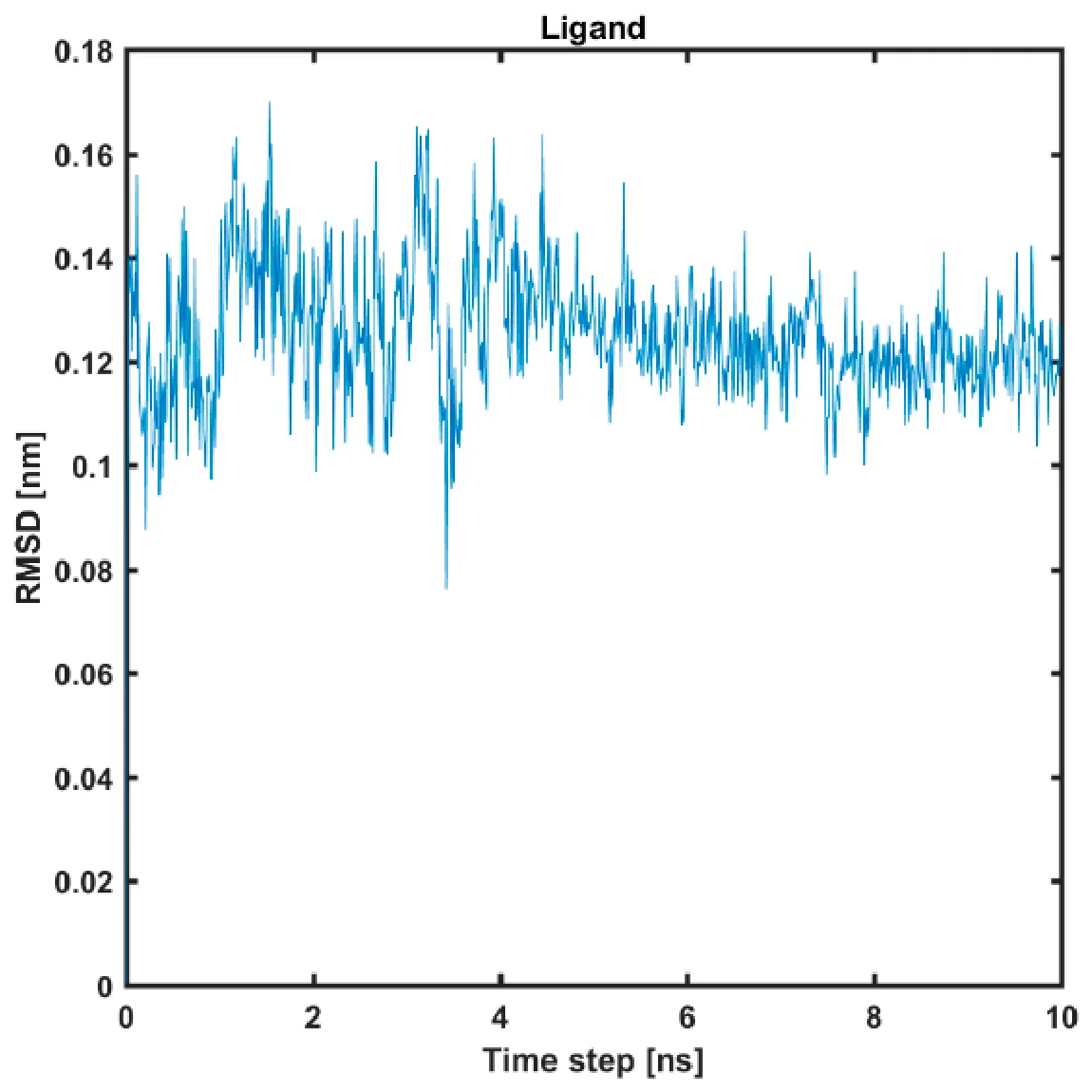
Root mean square deviations for molecule **4** in MDs run.

In fact, conformational stability and/or structural changes in the protein are confirmed by the relatively small variations in the RMSD values over the course of the MD simulations (≈1Å). It seems that the protein also remained stable in its compact (folded) form during 10 ns MD at 298 K producing relatively steady values of R_g_ with Δ_Rg_ ≈ 1Å. Similarly, compound **4** is characterised by small deviations in RMSD values (1), indicating only a minor change in the position of the ligand atom during MDs simulations (see Figure 8).

Based on the MDs findings, one can conclude that the coupling protocol produced a fairly stable Topo IIβ-Ligand complex.

It is important to note that the 10 ns MD simulation represents only a preliminary structural relaxation of the docked complex. Due to the lack of replicate runs and reference systems, these short-term stability trends should not be overinterpreted as direct evidence of biological enzyme inhibition or cleavage complex stabilisation.

### 3.2. Chemistry

The synthesis of selected derivatives of lomefloxacin containing a 1,4-disubstituted triazole scaffold was carried out using a two-step method, the key step involving copper(I)-catalysed azide–alkyne cycloaddition. In the first step, a propargyl group was introduced into the lomefloxacin structure, resulting in compound **1**. In the second step, azides from an internal library of compounds were used. Benzyl, *p*-Cl-benzyl, *p*-Br-benzyl, and *p*-NO_2_-benzyl azides were obtained by reaction of the corresponding bromide with sodium azide [34]. Phenyl azides were synthesised using a method involving diazonium salts. The appropriate aromatic amine was treated with sodium nitrite in an acidic medium at low temperature to form a diazonium salt, which was then reacted with sodium azide [35]. The resulting derivatives **2**–**9** were purified by column chromatography. The structures of the synthesised compounds were determined using spectroscopic methods, including NMR and HR MS (Supplementary Materials, File S1, Figures S2–S45). Assignment of resonance peaks in the ^13^C NMR spectra to specific carbon atoms within the structures of the title compounds was performed using two-dimensional HSQC and HMBC techniques. The 2D spectra also revealed that, in the ^1^H NMR spectrum, the signals from the protons of the methylene linker that connect the piperazine moiety to the 1,2,3-triazole system were obscured by the water signal. Furthermore, the signals from water and residual DMSO protons masked some of the resonance peaks associated with the 3-methylpiperazine ring.

**Figure 9:**
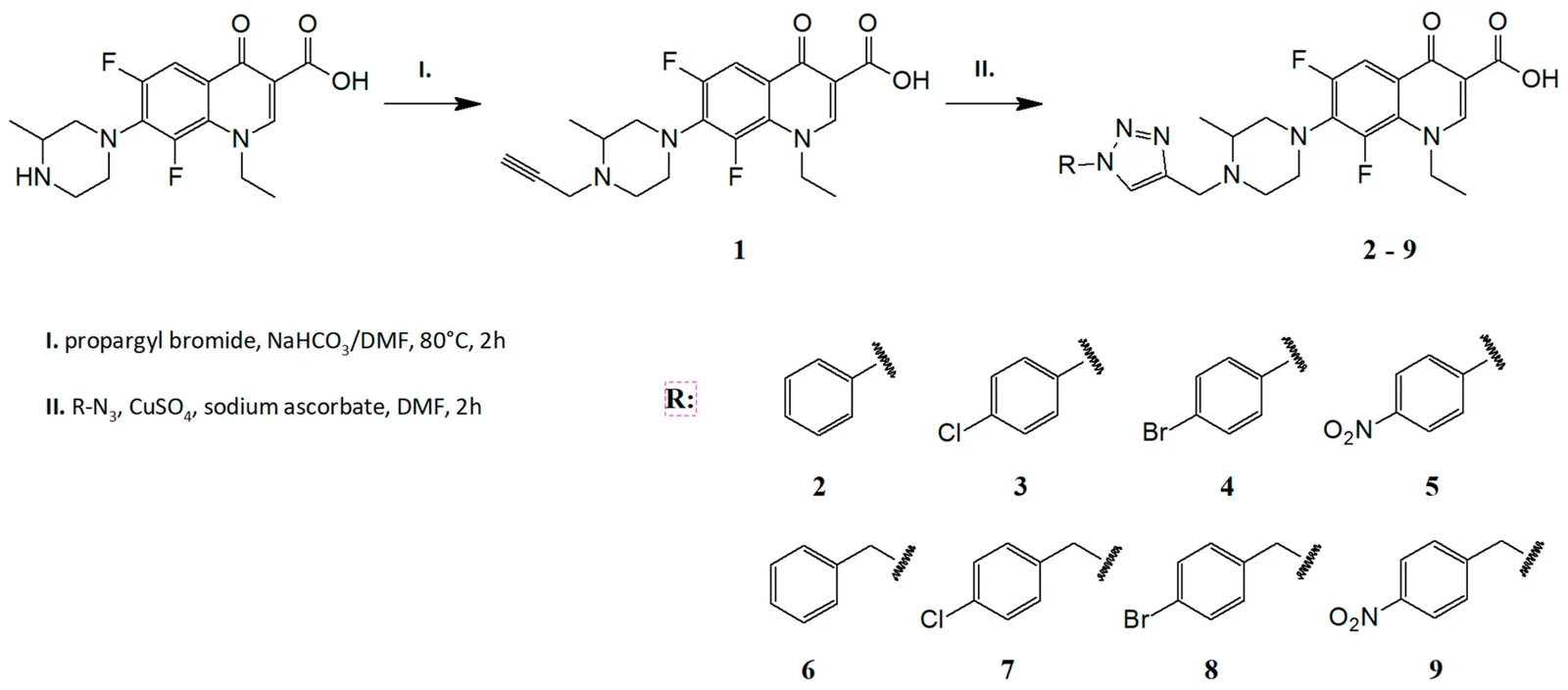
Synthesis of novel lomefloxacin derivatives.

The reagents and conditions used in the synthesis of the new lomefloxacin derivatives are shown in Figure 9.

### 3.3. Screening Analysis for Anticancer Activity

**Figure 10:**
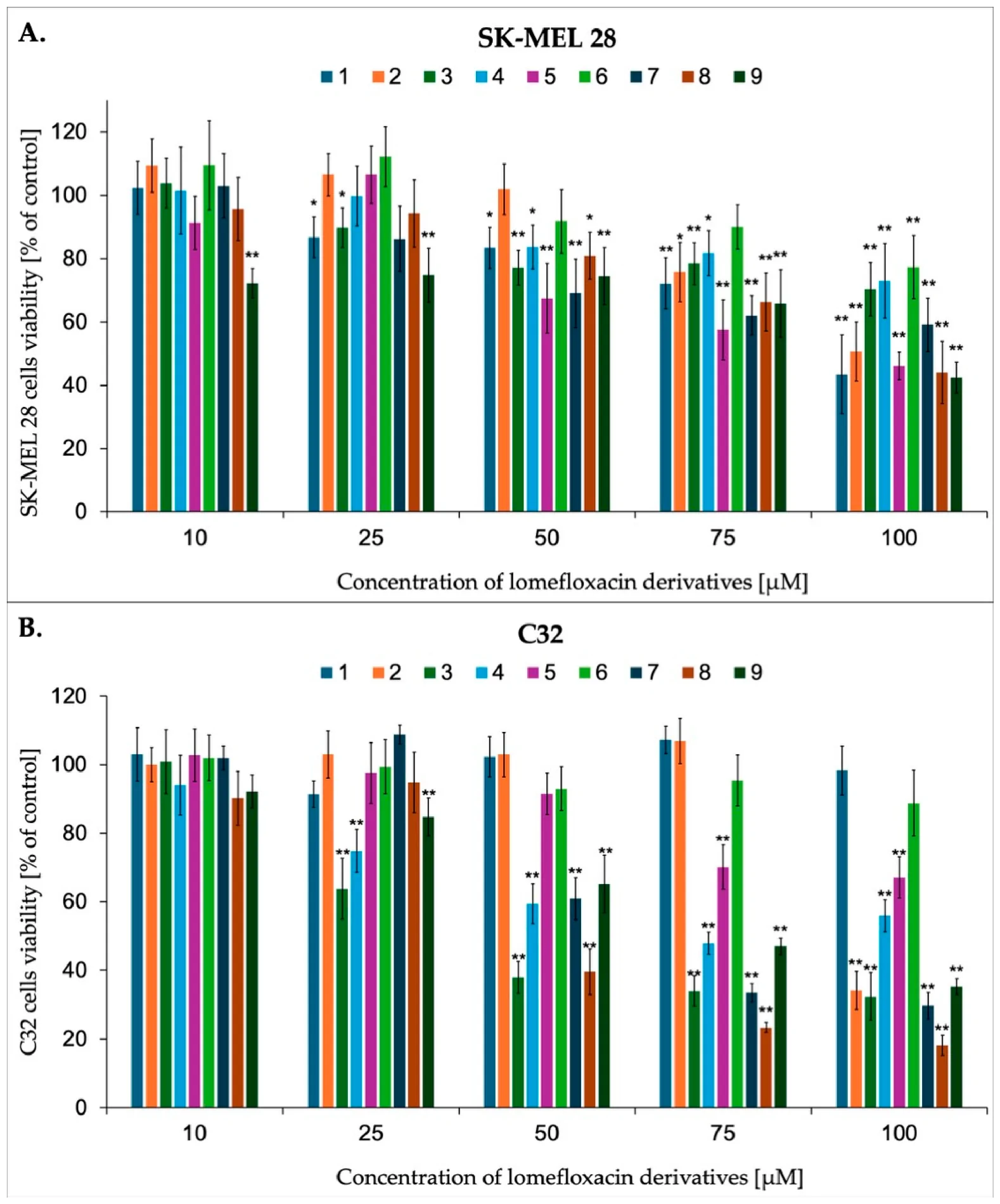
Effect of newly synthesised compounds **1**–**9** on the viability of SK-MEL 28 (**A**) and C32 (**B**) melanoma cells after 72 h of treatment. The results are expressed as a percentage of the control. The bars represent the mean ± SD (standard deviation) of three independent experiments; * *p* < 0.05 and ** *p* < 0.005.

**Figure 11:**
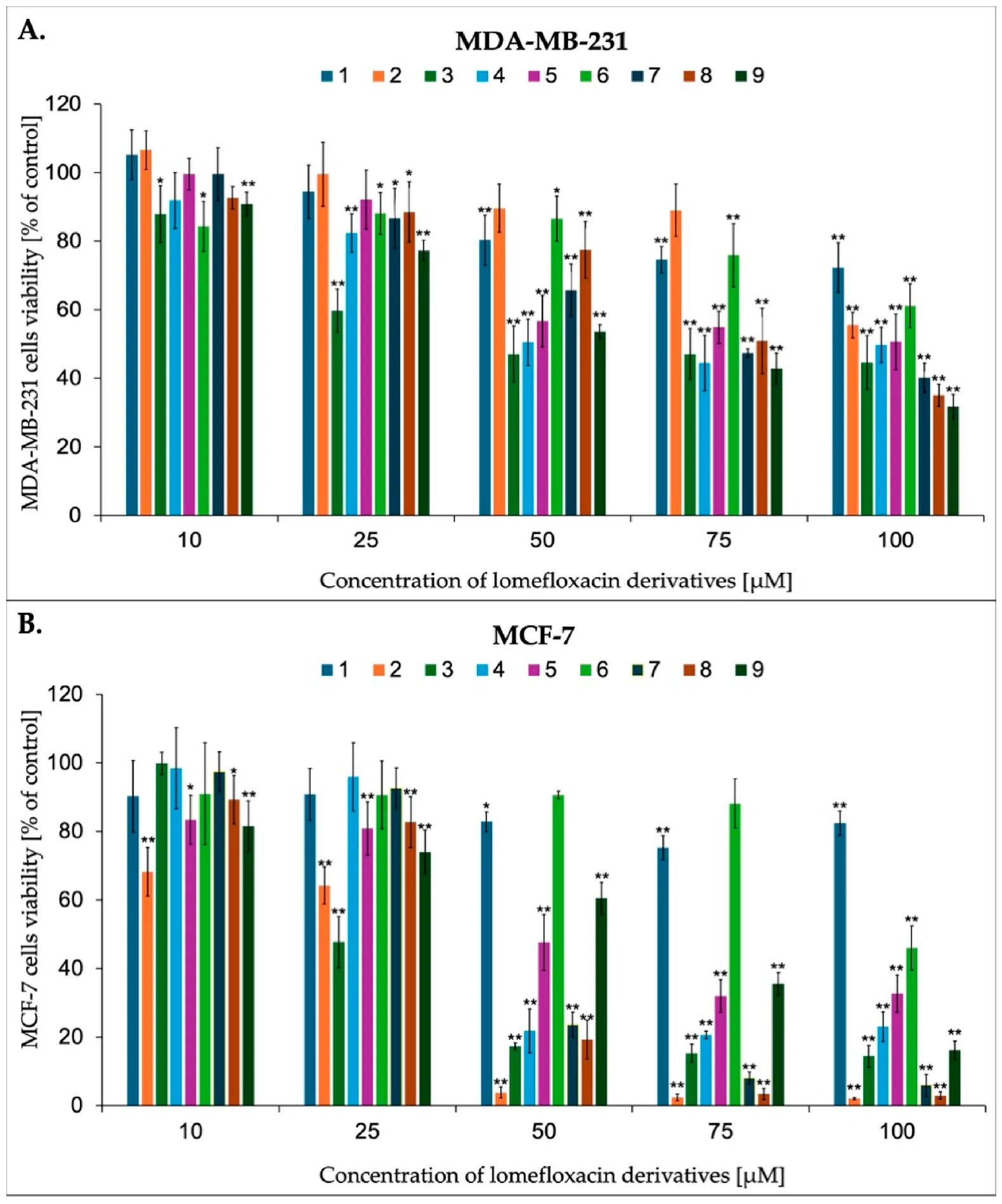
The effect of newly synthesised compounds **1**–**9** on the viability of MDA-MB-231 (**A**) and MCF-7 (**B**) breast cancer cells after 72 h of treatment. The results are expressed as a percentage of the control. The bars represent the mean ± SD (standard deviation) of three independent experiments; * *p* < 0.05 and ** *p* < 0.005.

**Figure 12:**
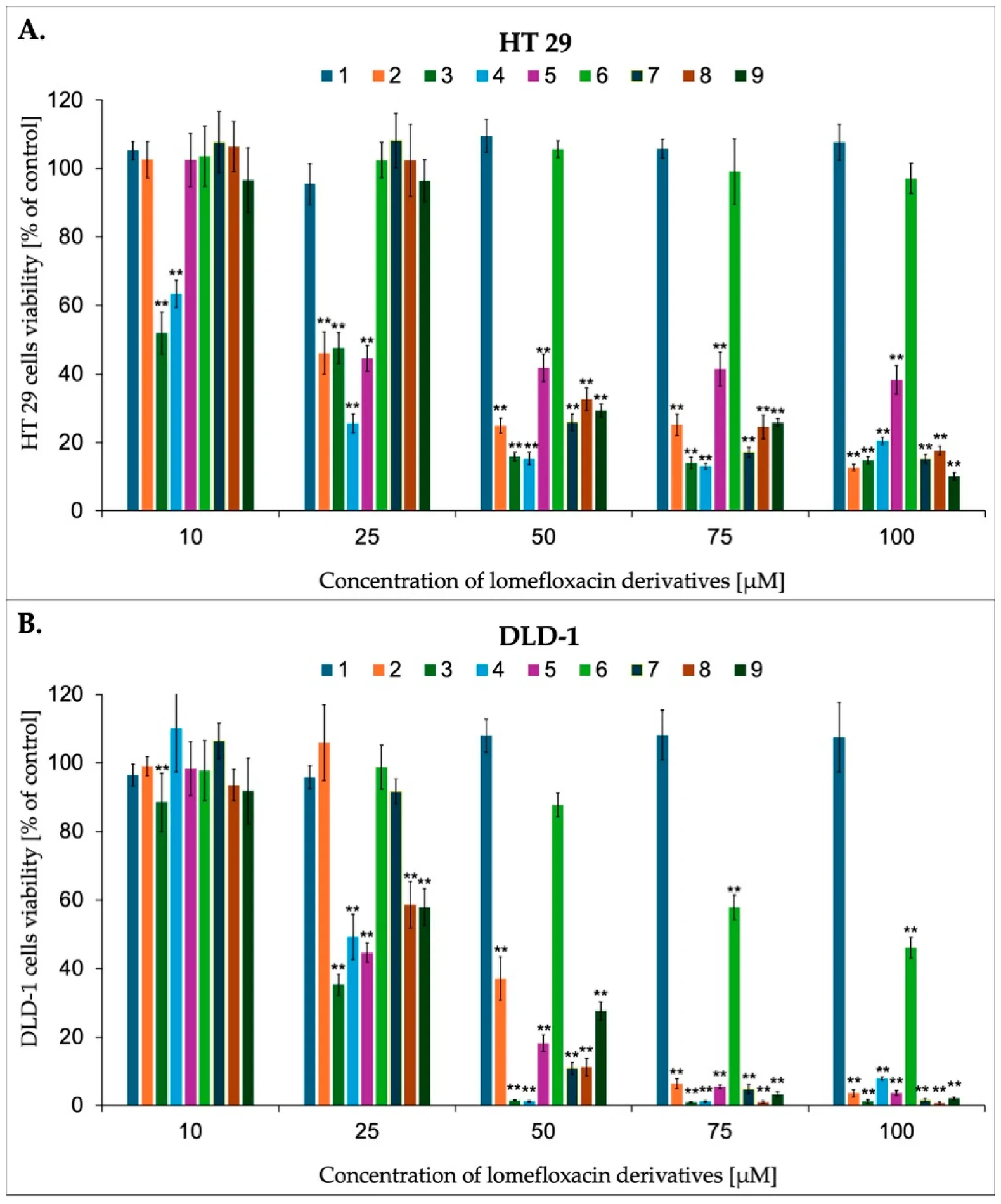
Effect of newly synthesised compounds **1**–**9** on the viability of HT 29 (**A**) and DLD-1 (**B**) colorectal adenocarcinoma cells after 72 h of treatment. The results are expressed as a percentage of the control. The bars represent the mean ± SD (standard deviation) of three independent experiments; ** *p* < 0.005.

To evaluate the anticancer potential of newly synthesised lomefloxacin derivatives, their cytotoxic activity was evaluated using a WST-1 assay in a panel of human cancer cell lines. The study included SK-MEL 28 and C32 (amelanotic melanoma), MDA-MB-231 (triple negative breast cancer), MCF-7 (hormone-dependent breast cancer), as well as HT 29 and DLD-1 (colorectal adenocarcinoma). The results of the antiproliferative evaluation are presented in Figure 10, Figure 11 and Figure 12. Table 2 summarises the calculated IC50 values for derivatives **1**–**9** and camptothecin for each cell line. The results of the WST-1 assay for camptothecin and lomefloxacin obtained for all analysed cell lines are presented in the Supplementary Materials (File S2, Figures S47–S53 and File S3, Figures S54–S60).

Analysis of the data revealed pronounced differences in the cytotoxic activity of the investigated derivatives across the tested cancer cell lines. The highest sensitivity to compounds was observed for the colorectal cancer cell lines HT 29 and DLD-1, which exhibited the lowest IC50 values among all cell models. In the HT 29 cell line, derivatives **3** and **4** showed particularly strong activity, with IC50 values of 13.4 and 13.6 μM, respectively. Compound **2** also demonstrated considerable cytotoxic potency, while the remaining active compounds exhibited IC50 values in the range of approximately 40–50 μM. A similar response pattern was observed for the DLD-1 cell line, where derivatives **3** and **4** again showed the highest efficacy, followed by derivatives **5**, **8** and **9**.

Among breast cancer cell lines, substantial differences in response to the investigated compounds were observed. The hormone-dependent MCF-7 cell line exhibited relatively high sensitivity, particularly to derivatives **2** and **3**, with IC50 values of 23.0 and 26.7 µM, respectively. Good to moderate activity was also recorded for derivatives **7** and **8**, whereas derivatives **1** and **6** were essentially inactive. On the contrary, the triple negative MDA-MB-231 cell line showed a markedly more resistant phenotype. Only derivatives **3** and **9** achieved IC50 values below 60 µM, while the remaining compounds showed limited activity or did not induce a significant cytotoxic effect. Differences in sensitivity between MCF-7 and MDA-MB-231 cells may be related to their distinct biological characteristics, including the status of hormone receptors and differences in signalling pathways involved in cell survival and proliferation.

Considerable differences in response were observed between melanoma cell lines. C32 cells exhibited moderate sensitivity to selected derivatives, with compound **3** and derivative **8** showing the highest activity. Most of the remaining compounds showed limited efficacy and for some derivatives, 50% inhibition of cell proliferation was not achieved within the tested concentration range. The SK-MEL 28 cell line was generally less sensitive to the compounds investigated, and most derivatives showing limited activity or IC50 values above the tested concentration range. In general, a comparison of all cell models demonstrated that the colorectal cancer cell lines HT 29 and DLD-1 were the most susceptible to the investigated derivatives, followed by the hormone-dependent breast cancer cell line MCF-7. On the contrary, MDA-MB-231 cells and both melanoma cell lines, particularly SK-MEL 28, exhibited considerably greater resistance. Among the entire series, derivatives **3** and **4** emerged as the most active compounds, showing the strongest effects against both colorectal cancer models and favourable activity against selected breast cancer cell lines.

Comparison of the results obtained with those of camptothecin, which is used as a reference compound, clearly demonstrated that the cytotoxic activity of all the investigated derivatives was substantially lower than that of the positive control. Camptothecin exhibited potent cytotoxic effects against all cell lines, with IC50 values ranging from 0.03 µM for HT 29 cells to 7.2 µM for the most resistant MDA-MB-231 cell line. Consequently, the IC50 values determined for the investigated derivatives were several hundred times higher than those recorded for camptothecin. This difference was particularly evident in HT 29 cells, where the most active derivatives, **3** and **4**, exhibited IC50 values of approximately 13 µM, whereas camptothecin showed activity at 0.03 µM. A similar trend was observed across the remaining cellular models, where even the most active derivatives did not reach the potency of the reference compound.

Structure–activity relationship analysis revealed that derivatives **3** and **4** exhibited the most favourable anticancer profiles, showing pronounced cytotoxic activity across multiple cancer cell lines. The compounds investigated could be divided into two structural subgroups that differed in the nature of the substituent at the 4 position of the 1,4-disubstituted triazole ring: derivatives **2**–**5** contained aryl substituents (phenyl, *p*-Cl-phenyl, *p*-Br-phenyl and *p*-NO_2_-phenyl), while derivatives **6**–**9** were characterised by the corresponding benzyl analogues. A direct comparison of the derivatives (**2**–**5**) with their corresponding benzyl analogues (**6**–**9**) revealed that the effect of introducing a methylene spacer was strongly dependent on the substituent. For derivatives containing a phenyl group, derivative **2** generally showed higher cytotoxic activity than its benzyl analogue **6**, particularly against MCF-7 and DLD-1 cells, although both compounds exhibited limited activity against melanoma cell lines. A similar trend was observed for the derivatives containing a chlorine atom, as compound **3** was more active than its benzyl analogue **7** against MDA-MB-231, MCF-7, HT 29 and DLD-1 cells. On the contrary, derivatives **4** and **8** containing a bromine atom showed comparable activity in several models, with compound **4** being notably more active against HT 29, while compound **8** showed higher activity against C32 and MCF-7 cells. For the derivatives containing a nitro group, derivative **5** generally displayed higher activity than its benzyl analogue **9** against HT 29 and DLD-1 cells, while compound **9** showed greater activity against melanoma cell lines, indicating that the influence of the methylene spacer on cytotoxic activity was dependent on substituents and cell lines. Compounds **3** and **4** demonstrated the most advantageous activity patterns, indicating that direct attachment of an aromatic ring to the triazole core may be more favourable to anticancer efficacy than introduction of an additional methylene spacer. The higher activity observed for the derivatives 3 and 4 compared to the *p*-nitro derivative 5 may result from a combination of steric, electronic and physicochemical effects. Although all three substituents are electron-withdrawing, the *p*-nitro group differs considerably from the halogen substituents in terms of polarity, hydrogen-bonding capacity and electronic characteristics, while the chlorine and bromine atoms provide greater polarizability and may favour different molecular interactions. Differences in lipophilicity may also contribute to the observed activity, as the introduction of these substituents can affect membrane permeability, cellular uptake, and intracellular distribution. However, this interpretation remains speculative, as the contribution of lipophilicity, steric, and electronic properties to the observed differences in activity would require further investigation using appropriate in silico calculations and/or experimental physicochemical and biological studies. However, the observed differences between the corresponding aryl and benzyl derivatives indicate that the effect of the methylene spacer depends on both the nature of the aromatic substituent and the cancer cell line. These observations identify the nature of the 4-position substituent in triazole as an important structural determinant of biological activity within this series of lomefloxacin–triazole hybrids. Importantly, the docking results did not show a consistent correlation with the antiproliferative effects observed in the cellular assays. The derivatives with the most favourable predicted binding were not necessarily those that showed the highest cytotoxic activity, suggesting that the predicted topoisomerase binding affinity alone is insufficient to explain the biological effects observed in vitro. Cellular activity may additionally be influenced by factors such as compound permeability, intracellular accumulation, metabolic stability, and interactions with other cellular targets. Accordingly, the proposed involvement of topoisomerases in the mechanism of action of the synthesised lomefloxacin–triazole hybrids remain hypothetical and requires experimental validation.

**Figure 13:**
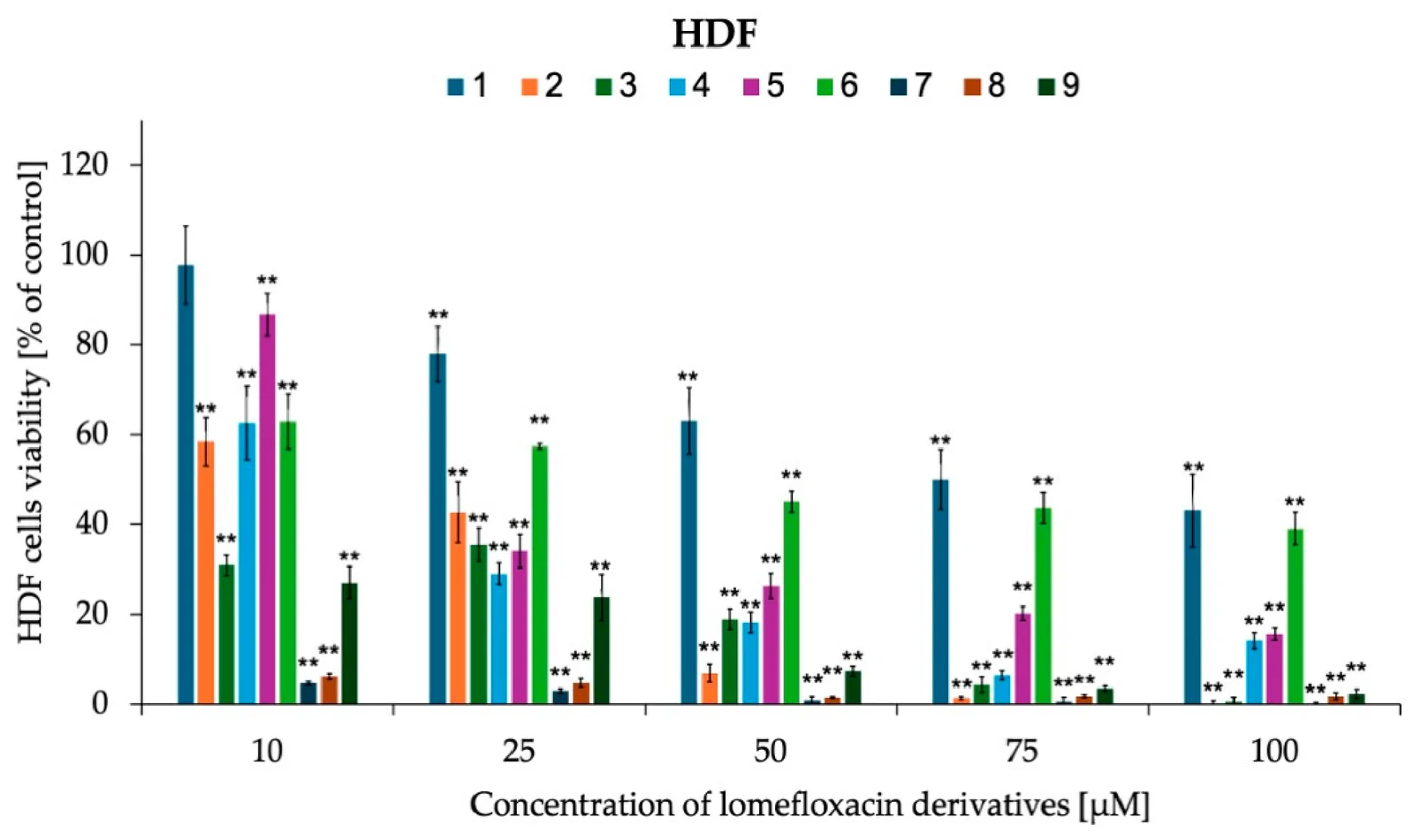
Effect of newly synthesised compounds **1**–**9** on HDF cell viability after 72 h of treatment. The results are expressed as a percentage of the control. The bars represent the mean ± SD (standard deviation) of three independent experiments.; ** *p* < 0.005.

To determine the selectivity of the cytotoxic effects of the compounds tested, their impact on normal human dermal fibroblasts (HDFs) was also evaluated (Figure 13).

Camptothecin, used as a reference compound, exhibited pronounced toxicity to fibroblasts (IC50 = 0.18 µM) while maintaining potent anticancer activity against all cancer cell lines. On the contrary, lomefloxacin showed substantially lower cytotoxic activity against both normal and cancer cells. No IC50 values below 100 µM were observed for HDF or any of the cancer cell lines, with the exception of MDA-MB-231 cells, for which an IC50 value of 27.3 µM was determined. These findings indicate that the parent lomefloxacin molecule exhibited limited cytotoxic activity under the experimental conditions used, particularly compared with camptothecin. Among the investigated derivatives, compounds **3** and **4** demonstrated the most favourable selectivity profiles. Despite its considerable activity against the HT 29 and DLD-1 cell lines, the compounds showed substantially lower effects on fibroblast viability, with IC50 values of 5.0 and 14.1 µM, respectively. Derivative **2** also exhibited a moderately favourable profile, showing activity against MCF-7 and HT 29 cells, while showing comparatively limited toxicity towards HDF cells (IC50 = 14.6 µM). Derivative 6 did not show pronounced selectivity towards any of the cancer cell lines, with IC50 values exceeding 100 µM for SK-MEL 28, C32 and MDA-MB-231 cells. Derivatives **1** and **5** exhibited the lowest toxicity in normal cells, with IC50 values of 76.7 and 22.9 µM, respectively; however, their anticancer activity was generally weak to moderate, limiting their therapeutic potential. A different pattern was observed for derivatives **7**, **8**, and **9**. In particular, derivative **8** showed pronounced toxicity to fibroblasts (IC50 = 0.1 µM), exceeding even camptothecin, while demonstrating substantially lower activity against all cancer cell lines. Similarly, derivative **7** (IC50 = 0.5 µM for HDF) showed poor selectivity, as its cytotoxic effect on normal fibroblasts was stronger than that observed in cancer cells. The derivative **9** exhibited moderate toxicity to HDF cells (IC50 = 3.8 µM); however, its IC50 values against cancer cell lines were consistently higher, indicating limited preferential activity toward malignant cells. In general, these findings suggest that derivatives **3** and **4** possess the most promising selectivity profiles among the investigated compounds, whereas derivatives **7** and **8** exhibit an unfavourable toxicological profile due to their pronounced effects on the viability of normal human fibroblasts.

Based on the IC50 values determined for HDF and the corresponding cancer cell lines, the selectivity index (SI) was calculated for each compound and the obtained values are presented in Table 3.

The calculated SI values indicate that most of the investigated lomefloxacin derivatives exhibited SI values below 1.0 in the tested cancer cell lines, reflecting a lack of preferential effect on cancer cell viability and a less favourable selectivity profile compared to the reference compound, camptothecin. Camptothecin showed higher SI values for several cancer cell lines, particularly HT 29 (SI = 6.0) and C32 (SI = 1.2), indicating a greater selectivity toward cancer cells. The parent lomefloxacin exhibited a more favourable selectivity profile than the investigated derivatives. However, due to the limitations imposed by IC50 values exceeding the highest concentration tested, selectivity indices could not be reliably determined for most cancer cell lines. Only for MDA-MB-231 cells could an SI > 3.663 be established, indicating a greater effect on cancer cell viability than on the viability of normal fibroblasts. Among the derivatives synthesised, compound **4** showed the most advantageous selectivity pattern, with an SI value close to 1.0 for HT 29 cells (SI = 1.037), suggesting comparable toxicity toward cancer and normal cells rather than pronounced selectivity. On the contrary, derivatives **7** and **8** exhibited consistently low SI values across the tested models, indicating substantial toxicity toward normal fibroblasts and an unfavourable selectivity profile. Overall, these findings indicate that several lomefloxacin derivatives exhibit limited selectivity between cancer and normal cells, suggesting that their antiproliferative effects may not be exclusively associated with tumour-specific mechanisms. The observed differences in cellular sensitivity may result from variations in proliferation rate, metabolic activity, and adaptive responses among the tested cell lines, which should be considered when interpreting the biological activity of the investigated compounds. Further structural optimisation of the lomefloxacin–triazole scaffold, combined with strategies aimed at improving tumour targeting, such as the application of selective drug delivery systems, may contribute to enhancing anticancer efficacy while reducing toxicity toward normal cells.

The results discussed above represent an innovative approach to the synthesis of lomefloxacin derivatives, as there are currently no reports describing analogous lomefloxacin-based compounds that incorporate a 1,4-disubstituted triazole motif. However, alternative strategies for the modification of this scaffold with potential anticancer activity have been reported, including metal complexation and classical functional derivatives. One of the first approaches involved copper(I) complexes that exhibited a lomefloxacin-derived motif, which demonstrated significantly enhanced cytotoxicity against CT26, A549, and MCF-7 cell lines compared to the parent compound and cisplatin, with their activity being attributed to DNA damage induction and increased oxidative stress [36]. Another approach involved the lomefloxacin amide derivatives described by Zhou et al. [37], which exhibited micromolar cytotoxic activity and were reported to inhibit topoisomerase II; in particular, compound **3e** showed an IC50 value of 0.98 µM against topoisomerase II, which was correlated with the disruption of DNA replication processes and cell cycle arrest. Further expansion of studies on the anticancer activity of lomefloxacin derivatives was presented in the work of Adly et al. [38], where a broad panel of NCI-60 cancer cell lines was evaluated, including MCF-7, MDA-MB-231, and HT 29; for the active compounds, GI_50_ values were obtained in the micromolar range, including 1.27 µM for MCF-7, 1.82 µM for MDA-MB-231, and 1.83 µM for HT 29, with maintained activity across multiple other tumour cell lines included in the NCI-60 panel. However, it should be noted that these GI_50_ values are not directly comparable with the IC50 values obtained in the present study using the WST-1 assay, as GI_50_ and IC50 represent different pharmacological endpoints and are determined using different experimental approaches.

In contrast, ciprofloxacin hybrids containing a 1,4-disubstituted 1,2,3-triazole scaffold represent one of the most extensively studied strategies for structural modification of fluoroquinolones to compounds with potential anticancer activity. In these systems, the application of “click chemistry” allows the precise conjugation of the quinolone pharmacophore with various aryl or heteroaryl substituents, leading to significant modulation of the physicochemical and biological properties of the parent molecule. In the study by Al-Taweel et al. [15], it was shown that the most active ciprofloxacin–triazole derivatives exhibited pronounced cytotoxic activity against U-87 MG (glioblastoma), MCF-7 and A549 (lung carcinoma) cell lines, with IC50 values of approximately 1.2 µM for U-87 MG and 10.6 µM for MCF-7, while showing substantially higher IC50 values against normal HDF fibroblasts (~170 µM), indicating a strong selectivity toward malignant cells. In the same study, these derivatives also demonstrated superior activity compared to cisplatin, whose IC50 values were significantly higher across all cancer cell lines, highlighting the importance of triazole modification in improving the biological potency of fluoroquinolones. Additional evidence for the anticancer potential of ciprofloxacin–triazole hybrids was provided in previous experimental studies [39], in which a series of triazole-linked ciprofloxacin derivatives were evaluated for cytotoxicity and safety profile. These compounds showed moderate to significant antiproliferative activity in cancer cell models while maintaining relatively low toxicity to normal HEK-293 cells, suggesting a favourable therapeutic window and indicating that incorporation may improve selectivity compared to the parent fluoroquinolone scaffold. Similar trends were reported in other studies on ciprofloxacin–1,2,3-triazole hybrids, where the incorporation of the triazole fragment increased lipophilicity, improved cell uptake, and improved interactions with molecular targets, resulting in micromolar cytotoxic activity against cell lines, such as MCF-7, A549, and HeLa [40]. Mechanistically, the activity of these compounds has been associated with reported effects on topoisomerase II, induction of oxidative stress, and activation of apoptotic pathways, indicating a potentially multifactorial mode of action. Furthermore, reviews of fluoroquinolone hybrids emphasise that 1,4-disubstituted triazole modifications can produce compounds with IC50 values in the low micromolar range (1–5 µM) in selected cancer models while maintaining relatively low toxicity toward normal cells, providing an important reference point for evaluating newly synthesised ciprofloxacin derivatives [41].

In relation to the presented results, it can be observed that some of the lomefloxacin derivatives **1**–**9** tested exhibit cytotoxic activity against MCF-7, MDA-MB-231, and HT 29 cell lines at a level within the micromolar range, which is consistent with previously reported fluoroquinolone-derived anticancer candidates. For the MCF-7 cell line, the values obtained for compounds **3**, **4**, **5**, and **7** to **9** fall within the range of approximately 26–50 µM, indicating moderate biological activity comparable to the data from the literature for lomefloxacin derivatives and ciprofloxacin modified with a 1,2,3-triazole moiety. A similar trend can be observed for the MDA-MB-231 and HT 29 cell lines. The lower potency observed for lomefloxacin-based derivatives compared to selected ciprofloxacin analogues suggests that replacement of the quinolone scaffold may significantly influence the biological performance of the resulting hybrids. Structural differences between ciprofloxacin and lomefloxacin, including the presence of an additional fluorine substituent at the C8 position and changes in the electronic properties of the quinolone core, may affect the physicochemical properties and cellular uptake, as well as interactions with biological macromolecules. Although both compounds share the quinolone pharmacophore, structural modification of the parent scaffold does not necessarily result in enhanced cytotoxic activity.

However, the obtained results demonstrate that lomefloxacin may represent an alternative scaffold for further exploration in the design of fluoroquinolone-based anticancer candidates. Among the investigated series, derivatives 3 and 4 exhibited the most pronounced antiproliferative activity, while the parent lomefloxacin showed only limited cytotoxic effects under experimental conditions. These findings indicate that the nature of the substituent introduced through the 1,2,3-triazole linker appears to influence cell line-dependent activity. Further optimisation of the lomefloxacin-based scaffold, combined with experimental evaluation of molecular targets potentially involved in the observed antiproliferative effects, may contribute to the development of more potent and selective derivatives.

## 4. Conclusions

In this work, a novel series of hybrids incorporating a 1,4-disubstituted 1,2,3-triazole scaffold was designed, synthesised and evaluated using both in silico and in vitro approaches. Molecular docking studies against topoisomerase I, IIα, and IIβ indicated that the investigated compounds may establish favourable interactions within the active sites of target enzymes, including hydrogen bonding, π–π stacking, and electrostatic interactions. These results indicate that the investigated compounds may interact with the analysed topoisomerases; however, molecular docking alone does not establish enzyme inhibition or confirm the involvement of these targets in the observed antiproliferative activity. The conformational stability of the protein–DNA complex and compound **4** was supported by the relatively minor fluctuations in the RMSD and radius of gyration (R_g_) values throughout the MD simulations, suggesting that the liganded (holo) protein–DNA system appeared to remain stable in its compact (folded) form.

The synthesis of compounds **2**–**9** was efficiently achieved via Cu(I)-catalysed azide–alkyne cycloaddition, demonstrating the applicability of click chemistry for the preparation of structurally diverse fluoroquinolone hybrids.

Biological evaluation revealed moderate cytotoxic activity of the synthesised derivatives against the cancer cell lines, with the highest sensitivity observed in models of colorectal cancer (HT 29 and DLD-1). Among the compounds investigated, derivatives **3** and **4** exhibited the most pronounced antiproliferative effects across several cancer cell lines; however, selectivity analysis revealed limited discrimination between cancer cells and normal human dermal fibroblasts (HDFs) for most derivatives. Comparison with previously reported fluoroquinolone–triazole hybrids indicated that the activity profiles are within the range observed for related compounds.

Overall, the results suggest that structural modification of the lomefloxacin scaffold provides a basis for further exploration in the development of fluoroquinolone-based anticancer candidates; however, additional optimisation and mechanistic studies are required to improve potency, selectivity, and elucidate the molecular mechanisms underlying their activity. Experimental studies are needed to determine whether the predicted interactions with topoisomerases translate into measurable effects on enzyme activity and contribute to the observed antiproliferative effects.

## Figures and Tables

**Table 1:** Molecular docking results obtained with GOLD for Topo I, Topo IIα and Topo IIβ modulators and the designed lomefloxacin derivatives **2**–**9**.

Compound	Topoisomerase I		Topoisomerase IIα		Topoisomerase IIβ	
	[a.u.]	[kcal/mol]	[a.u.]	[kcal/mol]	[a.u.]	[kcal/mol]
camptothecin	77.80	−9.94				
etoposide			86.93	−10.40	87.33	−10.42
**2**	67.49	−9.42	79.20	−10.01	79.61	−10.03
**3**	66.72	−9.38	80.80	−10.09	81.69	−10.14
**4**	76.02	−9.85	74.62	−9.78	83.31	−10.22
**5**	86.40	−10.38	74.30	−9.77	71.05	−9.60
**6**	73.17	−9.71	88.72	−10.49	74.16	−9.76
**7**	77.12	−9.91	87.06	−10.41	78.20	−9.96
**8**	80.45	−10.08	83.37	−10.22	82.59	−10.18
**9**	78.41	−9.97	83.19	−10.21	75.72	−9.84

**Table 2:** Concentration corresponding to 50% reduction in cell viability (IC50 [μM]) after 72 h of exposure to compounds **1**–**9**—results of the WST-1 assay obtained for normal human fibroblasts (HDFs) and melanoma cell lines (SK-MEL 28, C32), breast cancer cell lines (MDA-MB-231, MCF-7) and colon cancer cell lines (HT 29, DLD-1) incubated with the new compounds and lomefloxacin in a concentration range of 10–100 μM or with camptothecin at concentration range of 0.05–10 µM. Values of IC50 > 100 indicate that 50% reduction in cell viability was not reached at the highest concentration tested (100 μM).

Compound	HDF	SK-MEL 28	C32	MDA-MB-231	MCF-7	HT 29	DLD-1
camptothecin	0.18	0.84	0.15	7.2	1.9	0.03	3.13
lomefloxacin	>100	>100	>100	27.3	>100	>100	>100
**1**	76.7	97.2	>100	>100	>100	>100	>100
**2**	14.6	99.5	99.0	>100	23.0	29.2	46.7
**3**	5.0	>100	44.2	58.5	26.7	13.4	20.2
**4**	14.1	>100	90.0	72.1	41.9	13.6	24.9
**5**	22.9	87.3	>100	86.7	50.9	44.3	24.7
**6**	38.2	>100	>100	>100	98.0	>100	89.7
**7**	0.5	>100	63.4	74.5	40.1	43.6	36.3
**8**	0.1	94.0	47.9	77.8	35.8	47.0	27.4
**9**	3.8	>100	70.4	58.3	50.1	44.4	29.1

**Table 3:** Selectivity index (SI) values calculated for the investigated lomefloxacin derivatives **1**–**9**. Values preceded by <SI represent limiting selectivity index values calculated when the IC50 value exceeded the highest concentration tested (100 μM).

Compound	SK-MEL 28	C32	MDA-MB-231	MCF-7	HT 29	DLD-1
camptothecin	0.214	1.2	0.025	0.095	6.0	0.058
lomefloxacin	ND *	ND *	>3.663	ND *	ND *	ND *
**1**	0.789	<0.767	<0.767	<0.767	<0.767	<0.767
**2**	0.147	0.147	<0.146	0.635	0.500	0.313
**3**	<0.050	0.113	0.085	0.187	0.373	0.248
**4**	<0.141	0.157	0.196	0.337	1.037	0.566
**5**	0.262	<0.229	0.264	0.450	0.517	0.927
**6**	<0.382	<0.382	<0.382	0.390	<0.382	0.426
**7**	<0.005	0.008	0.007	0.012	0.011	0.014
**8**	0.001	0.002	0.001	0.003	0.002	0.004
**9**	<0.038	0.054	0.065	0.076	0.086	0.131

* ND means not determined; SI could not be reliably determined when both IC50 values were >100 μM.

## Data Availability

The original contributions presented in this study are included in the article and supplementary material. Further inquiries can be directed to the corresponding authors.

## Supplementary Materials

The following supporting information can be downloaded at https://www.mdpi.com/article/10.3390/biom16091323/s1, File S1: Figure S1. The compound numbering system tested; Figure S2. ^1^H NMR spectra of compound **1**; Figure S3. ^13^C NMR spectra of compound **1**; Figure S4. ^19^F NMR spectra of compound **1**; Figure S5. HR MS spectra of compound **1**; Figure S6. HPLC analysis of compound **1**; Figure S7. ^1^H NMR spectra of compound **2**; Figure S8. ^13^C NMR spectra of compound **2**; Figure S9. ^19^F NMR spectra of compound **2**; Figure S10. HR MS spectra of compound **2**; Figure S11. HPLC analysis of compound **2**; Figure S12. ^1^H NMR spectra of compound **3**; Figure S13. ^13^C NMR spectra of compound **3**; Figure S14. ^19^F NMR spectra of compound **3**; Figure S15. HR MS spectra of compound **3**; Figure S16. HPLC analysis of compound **3**; Figure S17. ^1^H NMR spectra of compound **4**; Figure S18. ^13^C NMR spectra of compound **4**; Figure S19. ^19^F NMR spectra of compound **4**; Figure S20. HR MS spectra of compound **4**; Figure S21. HPLC analysis of compound **4**; Figure S22. ^1^H NMR spectra of compound **5**; Figure S23. ^13^C NMR spectra of compound **5**; Figure S24. ^19^F NMR spectra of compound **5**; Figure S25. HR MS spectra of compound **5**; Figure S26. HPLC analysis of compound **5**; Figure S27. ^1^H NMR spectra of compound **6**; Figure S28. ^13^C NMR spectra of compound **6**; Figure S29. ^19^F NMR spectra of compound **6**; Figure S30. HR MS spectra of compound **6**; Figure S31. HPLC analysis of compound **6**; Figure S32. ^1^H NMR spectra of compound **7**; Figure S33. ^13^C NMR spectra of compound **7**; Figure S34. ^19^F NMR spectra of compound **7**; Figure S35. HR MS spectra of compound **7**; Figure S36. HPLC analysis of compound **7**; Figure S37. ^1^H NMR spectra of compound **8**; Figure S38. ^13^C NMR spectra of compound **8**; Figure S39. ^19^F NMR spectra of compound **8**; Figure S40. HR MS spectra of compound **8**; Figure S41. HPLC analysis of compound **8**; Figure S42. ^1^H NMR spectra of compound **9**; Figure S43. ^13^C NMR spectra of compound **9**; Figure S44. ^19^F NMR spectra of compound **9**; Figure S45. HR MS spectra of compound **9**; Figure S46. HPLC analysis of compound **9**; File S2: Figure S47. Effect of camptothecin on the viability of SK-MEL 28 cells after 72 h of treatment. The results are expressed as a percentage of the control. The bars represent the mean ± SD (standard deviation) of three independent experiments.; * *p* < 0.05 and ** *p* < 0.005; Figure S48. The effect of camptothecin on the viability of C32 cells after 72 h of treatment. The results are expressed as a percentage of control. Bars represent the mean ± SD (standard deviation) of three independent experiments.; * *p* < 0.05 and ** *p* < 0.005; Figure S49. The effect of the camptothecin on the viability of MDA MB 231 cells after 72 h of treatment. The results are expressed as a percentage of control. Bars represent the mean ± SD (standard deviation) of three independent experiments.; * *p* < 0.05 and ** *p* < 0.005; Figure S50. The effect of the camptothecin on the viability of MCF-7 cells after 72 h of treatment. The results are expressed as a percentage of control. Bars represent the mean ± SD (standard deviation) of three independent experiments.; * *p* < 0.05 and ** *p* < 0.005; Figure S51. The effect of the camptothecin on the viability of HT 29 cells after 72 h of treatment. The results are expressed as a percentage of control. Bars represent the mean ± SD (standard deviation) of three independent experiments.; * *p* < 0.05 and ** *p* < 0.005; Figure S52. The effect of the camptothecin on the viability of DLD-1 cells after 72 h of treatment. The results are expressed as a percentage of control. Bars represent the mean ± SD (standard deviation) of three independent experiments.; * *p* < 0.05 and ** *p* < 0.005; Figure S53. The effect of HDF cell camptothecin on the viability of HDF cells after 72 h of treatment. The results are expressed as a percentage of control. Bars represent the mean ± SD (standard deviation) of three independent experiments.; * *p* < 0.05 and ** *p* < 0.005; File S3: Figure S54. Effect of lomefloxacin on the viability of SK-MEL 28 cells after 72 h of treatment. The results are expressed as a percentage of control. Bars represent the mean ± SD (standard deviation) of three independent experiments.; ** *p* < 0.005; Figure S55. The effect of lomefloxacin on the viability of C32 cells after 72 h of treatment. The results are expressed as a percentage of control. Bars represent the mean ± SD (standard deviation) of three independent experiments. ** *p* < 0.005; Figure S56. The effect of lomefloxacin on the viability of MDA MB 231 cells after 72 h of treatment. The results are expressed as a percentage of control. Bars represent the mean ± SD (standard deviation) of three independent experiments.; ** *p* < 0.005; Figure S57. The effect of the lomefloxacin on the viability of MCF-7 cells after 72 h of treatment. The results are expressed as a percentage of control. Bars represent the mean ± SD (standard deviation) of three independent experiments. ** *p* < 0.005; Figure S58. The effect of the lomefloxacin on HT 29 cells after 72 h of treatment. The results are expressed as a percentage of control. Bars represent the mean ± SD (standard deviation) of three independent experiments.** *p* < 0.005; Figure S59. The effect of the lomefloxacin on the viability of DLD-1 cells after 72 h of treatment. The results are expressed as a percentage of control. Bars represent the mean ± SD (standard deviation) of three independent experiments.; Figure S60. The effect of HDF cell lomefloxacin on the viability of HDF cells after 72 h of treatment. The results are expressed as a percentage of control. Bars represent the mean ± SD (standard deviation) of three independent experiments.

## Abbreviations

The following abbreviations are used in this manuscript:

MD	Molecular dynamics
FBS	Foetal Bovine Serum
DFT	Density Functional Theory
