# Supplementary material for: Novel Lomefloxacin–Triazole Hybrids: Design, Synthesis and In Vitro Antiproliferative Activity in Breast, Melanoma and Colon Cancer Cell Lines
Source: Biomolecules. 2026 Sep 11;16(9):1323. doi: 10.3390/biom16091323 (PMC13604923; doi:10.3390/biom16091323)
Supplement: Supplementary file 1 [file biomolecules-16-01323-s001.zip › Supp_Mat_F2-WST-1 camptothecin.pdf]

Supplementary Materials, File 2

Journal: Biomolecules (MDPI)

Manuscript Title: **Novel lomefloxacin-triazole hybrids: design, synthesis and *in vitro* antiproliferative activity on breast, melanoma and colon cancer cell lines**

Authors: Justyna Nowakowska, Zuzanna Rzepka, Andrzej Bak, Dorota Wrześniok, Krzysztof Marciniec

Supplementary Methods

Methods S2. WST-1 assay for camptothecin

Cell viability of the tested cell lines was assessed using the WST-1 reagent (Roche, Mannheim, Germany), which allows for the evaluation of cellular proliferation. Cells were seeded into 96-well plates at a density of 3×10^3 cells per well and incubated for 24 hours. Thereafter, the culture medium was replaced with solutions of camptothecin and incubated for an additional 72 hours. WST-1 reagent was added 2 hours before the end of incubation, and absorbance was measured using the microplate reader Infinite 200 Pro (TECAN, Mannedorf, Switzerland). The mean of the control wells was set as 100%, and dose-response curves along with the half-maximal inhibitory concentration (IC50), were determined using GraphPad Prism 10 (GraphPad Software, San Diego, CA, USA).

Supplementary Figures

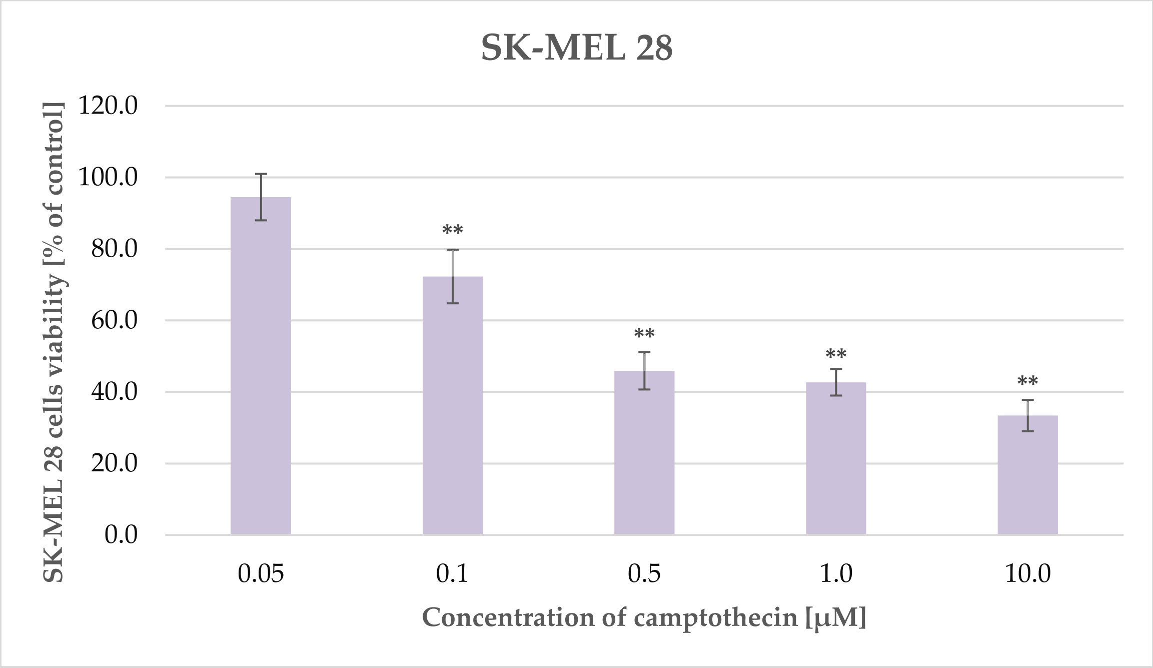

Figure S47. The effect of the camptothecin on the viability of SK-MEL 28 cells after 72 hours of treatment. The results are expressed as a percentage of control. Bars represent the mean ± SD (standard deviation) of three independent experiments.; * $p < 0.05$ and ** $p < 0.005$

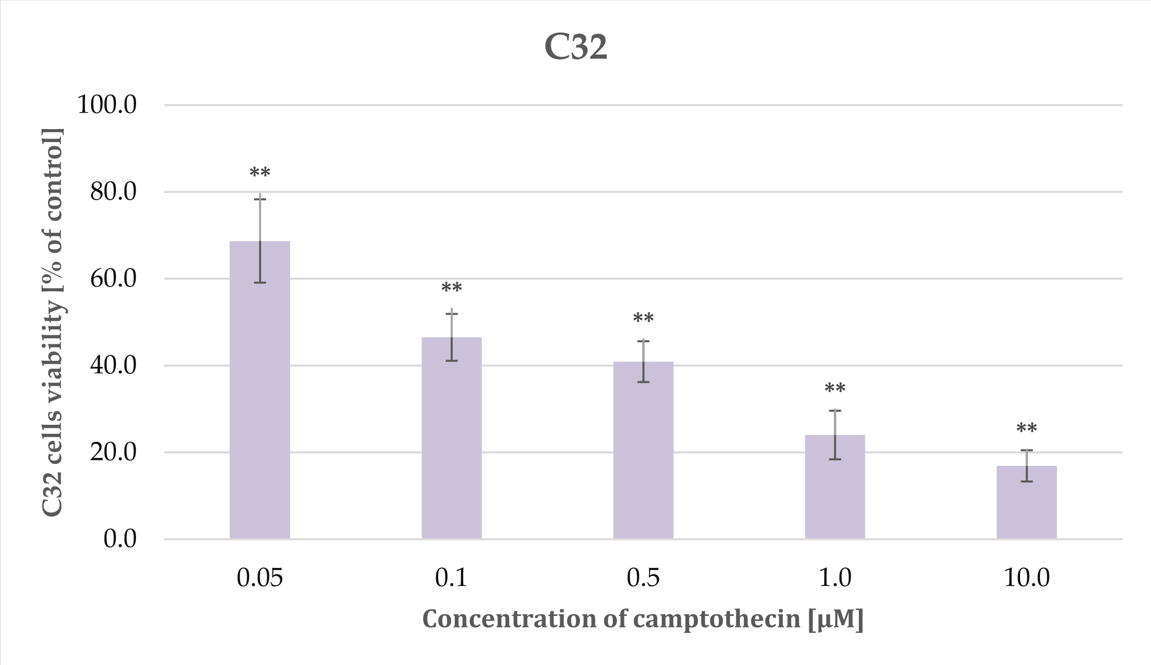

Figure S48. The effect of the camptothecin on the viability of C32 cells after 72 hours of treatment. The results are expressed as a percentage of control. Bars represent the mean ± SD (standard deviation) of three independent experiments.; * $p < 0.05$ and ** $p < 0.005$

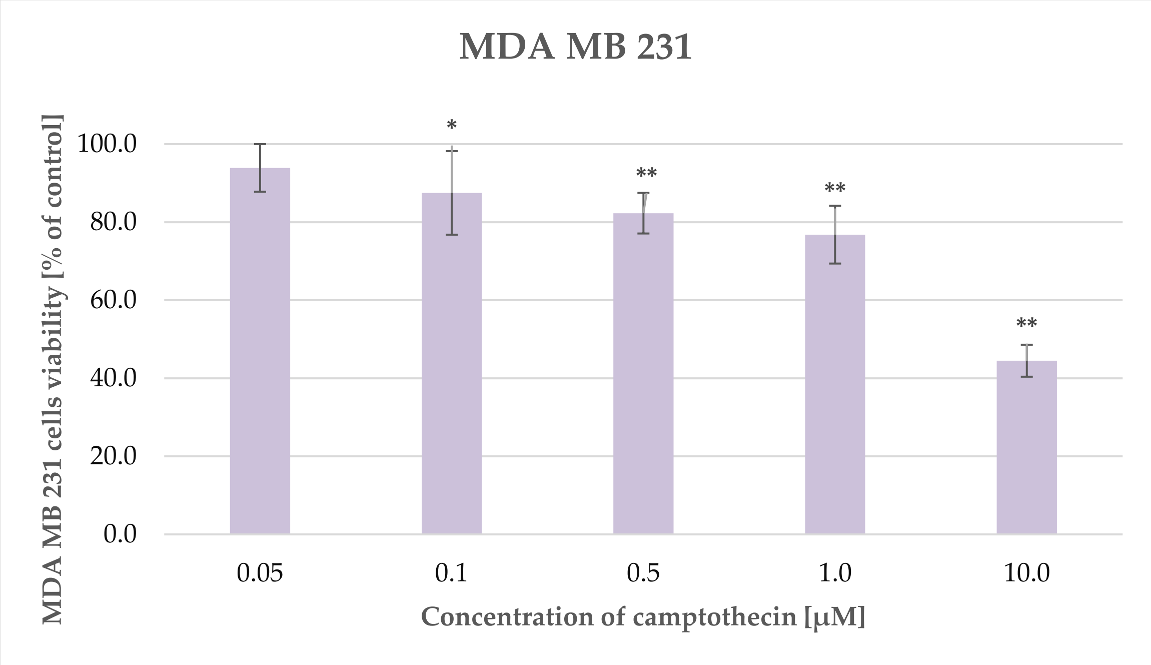

Figure S49. The effect of the camptothecin on the viability of MDA MB 231 cells after 72 hours of treatment. The results are expressed as a percentage of control. Bars represent the mean ± SD (standard deviation) of three independent experiments.; * $p < 0.05$ and ** $p < 0.005$

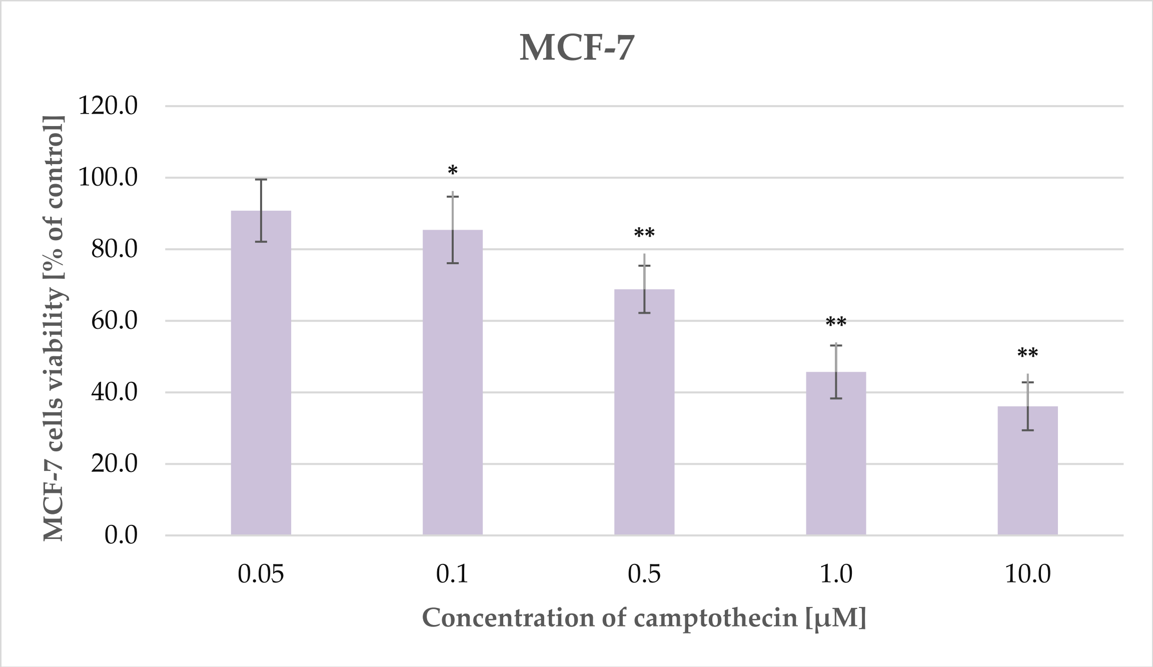

Figure S50. The effect of the camptothecin on the viability of MCF-7 cells after 72 hours of treatment. The results are expressed as a percentage of control. Bars represent the mean ± SD (standard deviation) of three independent experiments.; * $p < 0.05$ and ** $p < 0.005$

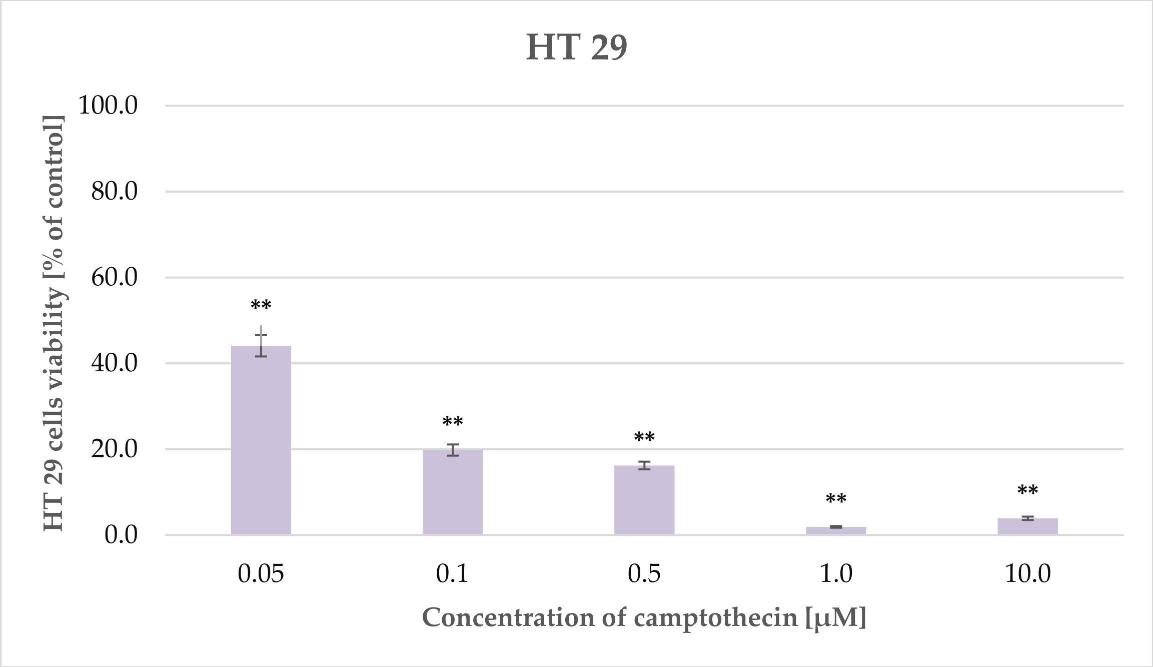

Figure S51. The effect of the camptothecin on the viability of HT 29 cells after 72 hours of treatment. The results are expressed as a percentage of control. Bars represent the mean ± SD (standard deviation) of three independent experiments.; * $p < 0.05$ and ** $p < 0.005$

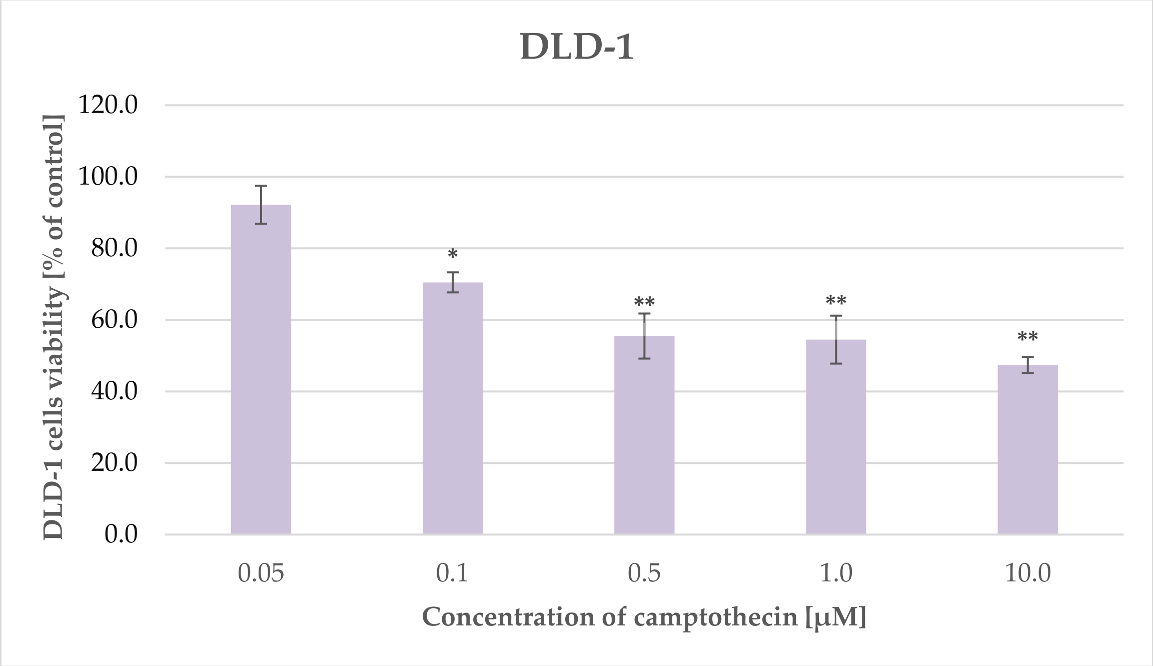

Figure S52. The effect of the camptothecin on the viability of DLD-1 cells after 72 hours of treatment. The results are expressed as a percentage of control. Bars represent the mean ± SD (standard deviation) of three independent experiments.; * $p < 0.05$ and ** $p < 0.005$

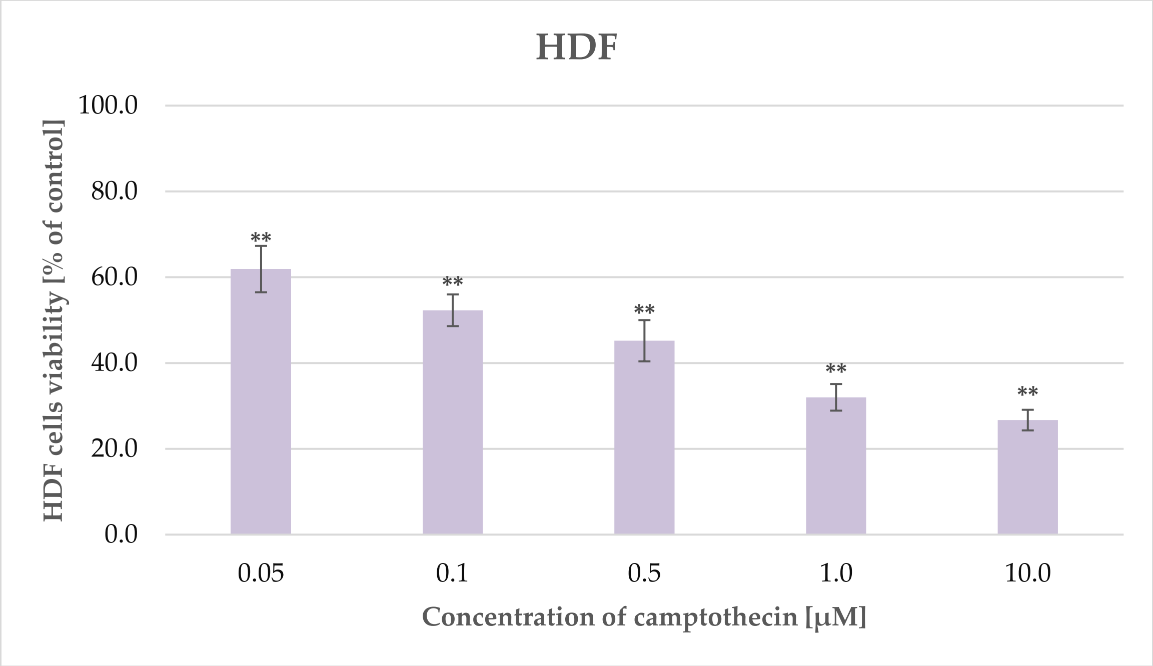

Figure S53. The effect of the camptothecin on the viability of HDF cells after 72 hours of treatment. The results are expressed as a percentage of control. Bars represent the mean ± SD (standard deviation) of three independent experiments.; * $p < 0.05$ and ** $p < 0.005$
