# Supplementary material for: Novel Lomefloxacin–Triazole Hybrids: Design, Synthesis and In Vitro Antiproliferative Activity in Breast, Melanoma and Colon Cancer Cell Lines
Source: Biomolecules. 2026 Sep 11;16(9):1323. doi: 10.3390/biom16091323 (PMC13604923; doi:10.3390/biom16091323)
Supplement: Supplementary file 1 [file biomolecules-16-01323-s001.zip › Supp_Mat_F1-NMR_MS_HPLC.pdf]

Supplementary Materials, File 1

Journal: Biomolecules (MDPI)

Manuscript Title: Novel lomefloxacin-triazole hybrids: design, synthesis and *in vitro* antiproliferative activity on breast, melanoma and colon cancer cell lines

Authors: Justyna Nowakowska, Zuzanna Rzepka, Andrzej Bąk, Dorota Wrześniok, Krzysztof Marciniec

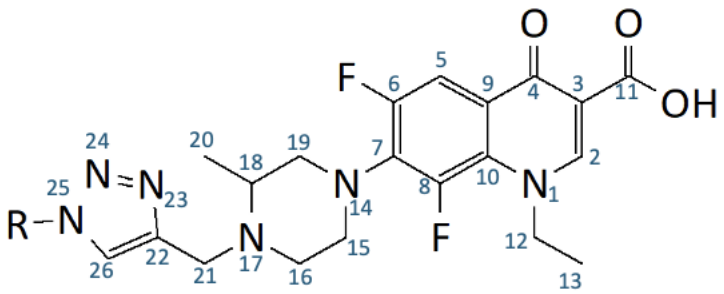

2-9

R:

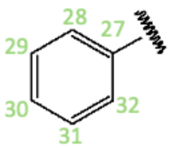

2

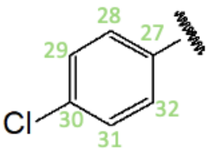

3

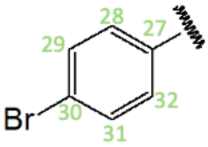

4

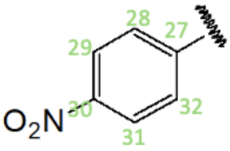

5

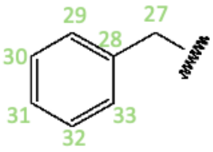

6

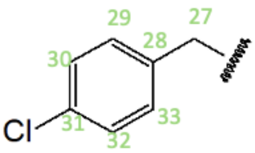

7

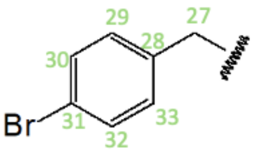

8

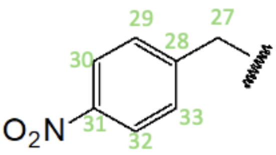

9

Figure S1. The numbering system of compounds tested

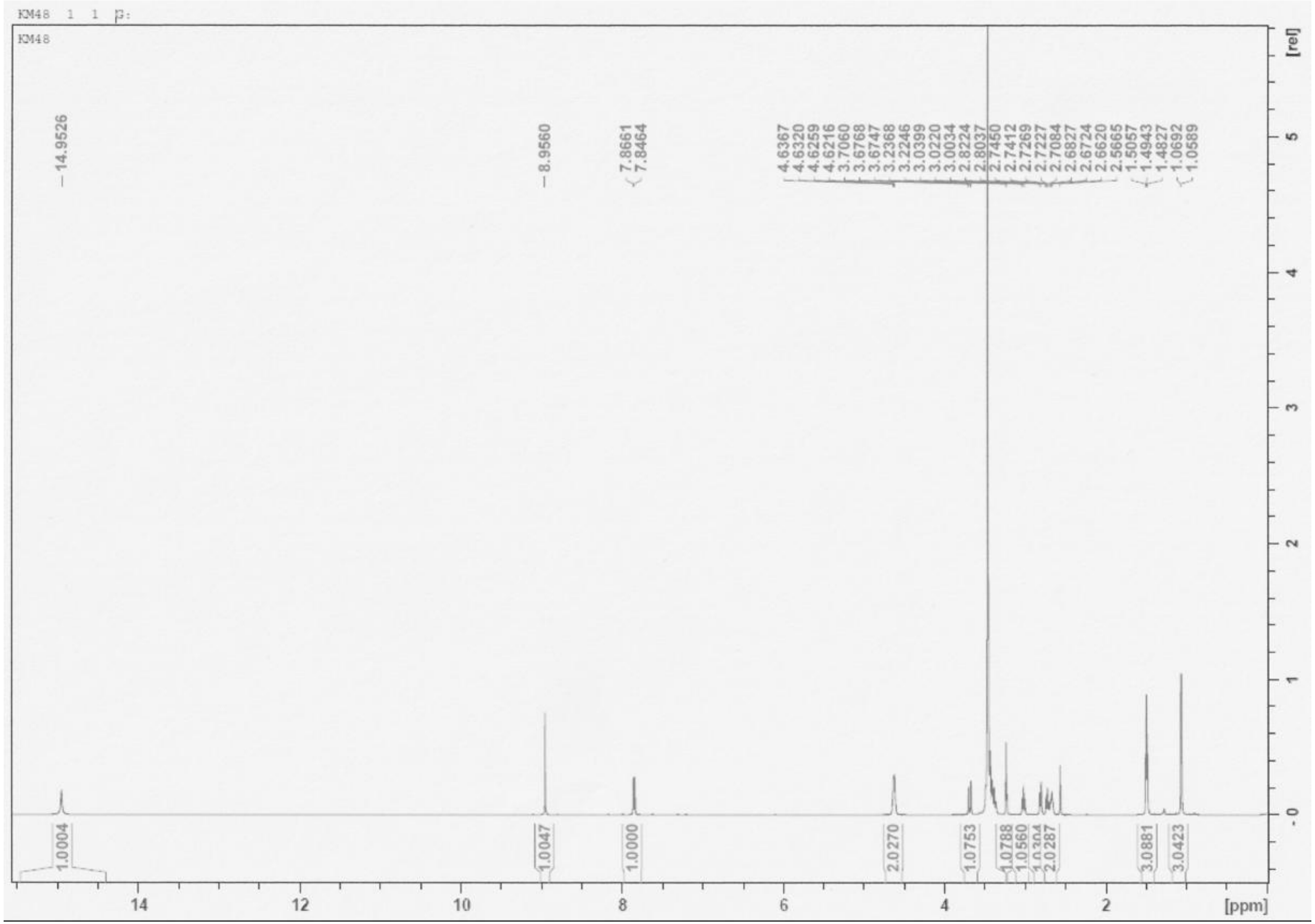

Figure S2. ^{1}H NMR spectra of compound **1**

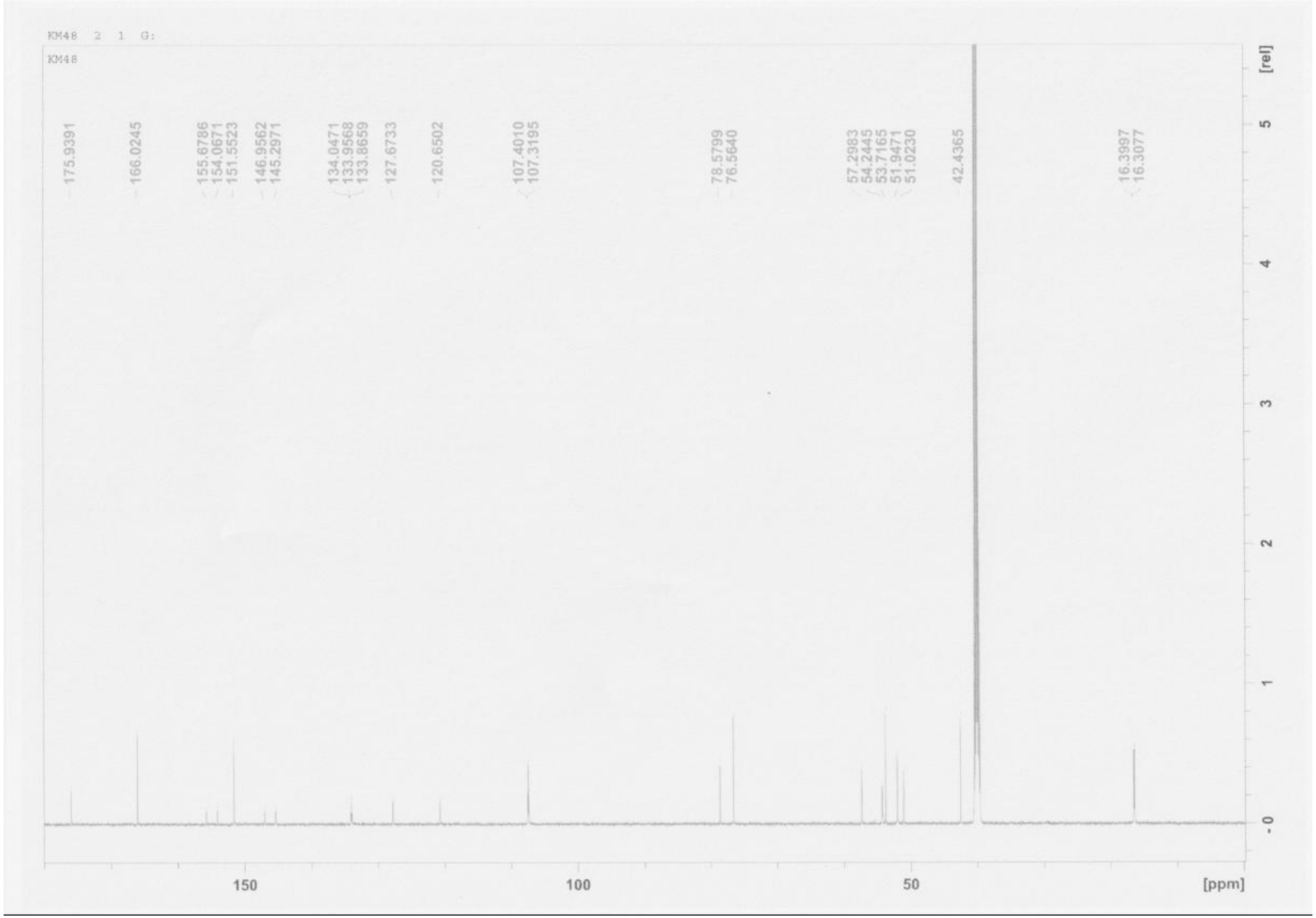

Figure S3. ^{13}C NMR spectra of compound **1**

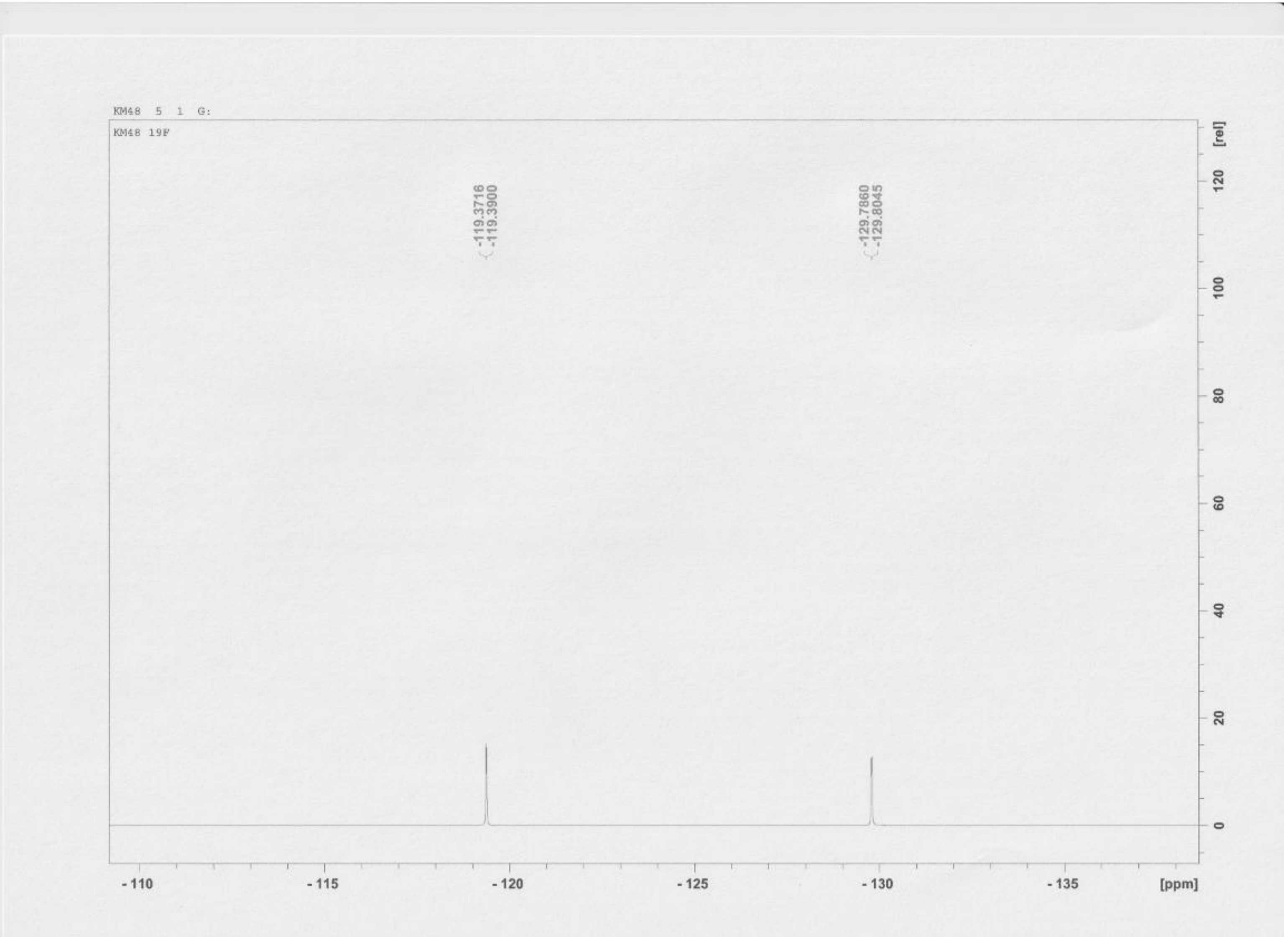

Figure S4. ^{19}F NMR spectra of compound **1**

Compound Spectrum List Report

Analysis Info

Analysis Name D:\Data\Iome_N_prop.d
Method low_mass_positive.m
Sample Name Tune_neg_high_post
Comment

Acquisition Date 4/9/2025 12:31:32 PM

Operator KM
Instrument impact II 1825265.10082

Acquisition Parameter

Source Type	ESI	Ion Polarity	Positive	Set Nebulizer	0.3 Bar
Focus	Active	Set Capillary	4000 V	Set Dry Heater	200 °C
Scan Begin	100 m/z	Set End Plate Offset	-500 V	Set Dry Gas	3.0 l/min
Scan End	1000 m/z	Set Charging Voltage	2000 V	Set Divert Valve	Source
		Set Corona	0 nA	Set APCI Heater	0 °C

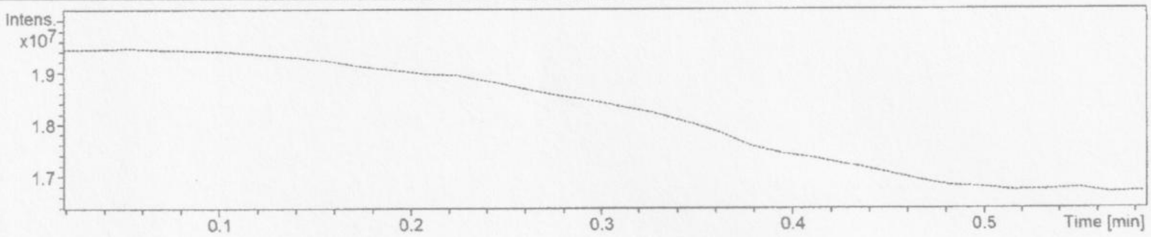

+MS, 0.0-0.6min #1-34

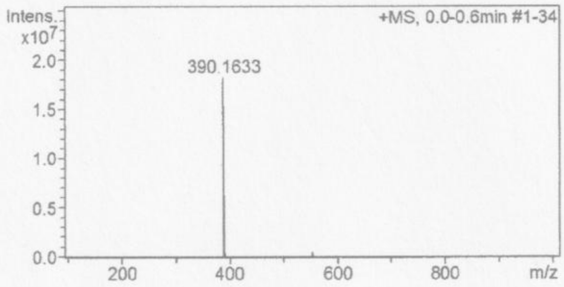

#	m/z	Res.	S/N	I	I %	FWHM
1	390.1633	31110	56140.9	18196762	100.0	0.0125
2	391.1659	34074	11983.3	3896564	21.4	0.0115
3	392.1682	21719	1482.2	482634	2.7	0.0181

Iome_N_prop.d
Bruker Compass DataAnalysis 4.3 printed: 4/9/2025 12:33:04 PM by: KM Page 1 of 1

Figure S5. HR MS spectra of compound **1**

Chromatogram and Results

Injection Details

Injection Name:	GD1	*Run Time (min):*	10.00
Vial Number:	RA1	*Injection Volume:*	5.00
Injection Type:	Unknown	*Channel:*	UV_VIS_2
Instrument Method:	HPLC 1 seria 0.1	*Bandwidth:*	2
Processing Method:	LC MS	*Dilution Factor:*	1.0000
Injection Date/Time:	07/Jul/26 11:21	*Sample Weight:*	1.0000

Chromatogram

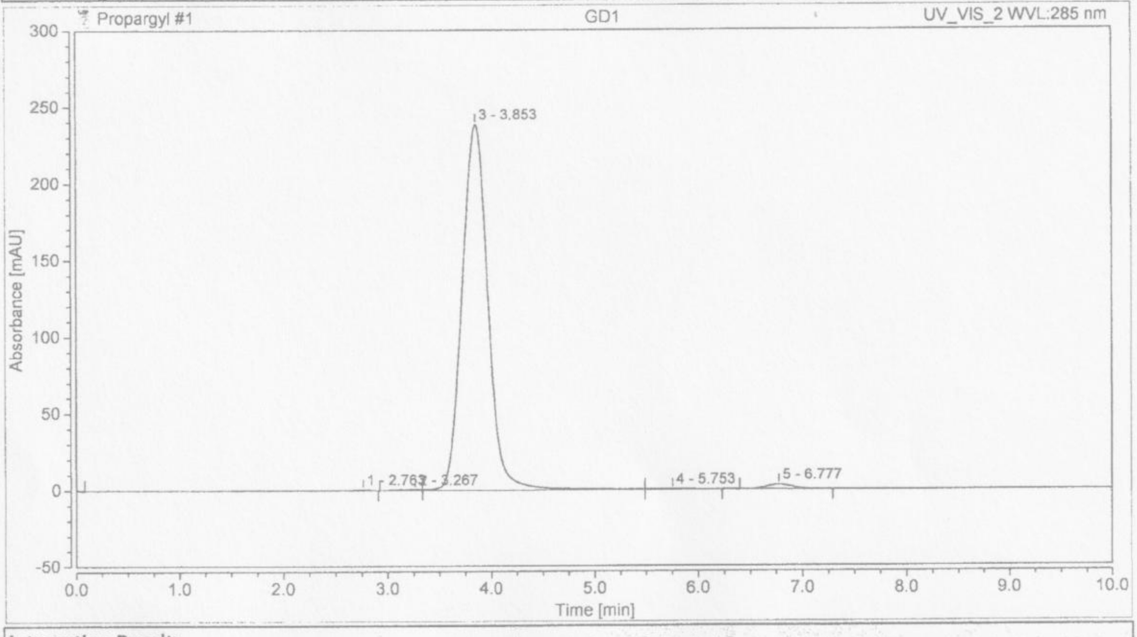

Integration Results

No.	Peak Name	Retention Time min	Area mAU*min	Height mAU	Relative Area %	Relative Height %	Amount n.a.
1		2.763	0.306	0.568	0.44	0.23	n.a.
2		3.267	0.143	0.457	0.20	0.19	n.a.
3		3.853	68.679	238.633	97.97	98.21	n.a.
4		5.753	0.099	0.233	0.14	0.10	n.a.
5		6.777	0.871	3.085	1.24	1.27	n.a.
**Total:**			**70.098**	**242.977**	**100.00**	**100.00**	

Figure S6. HPLC analysis of compound **1**

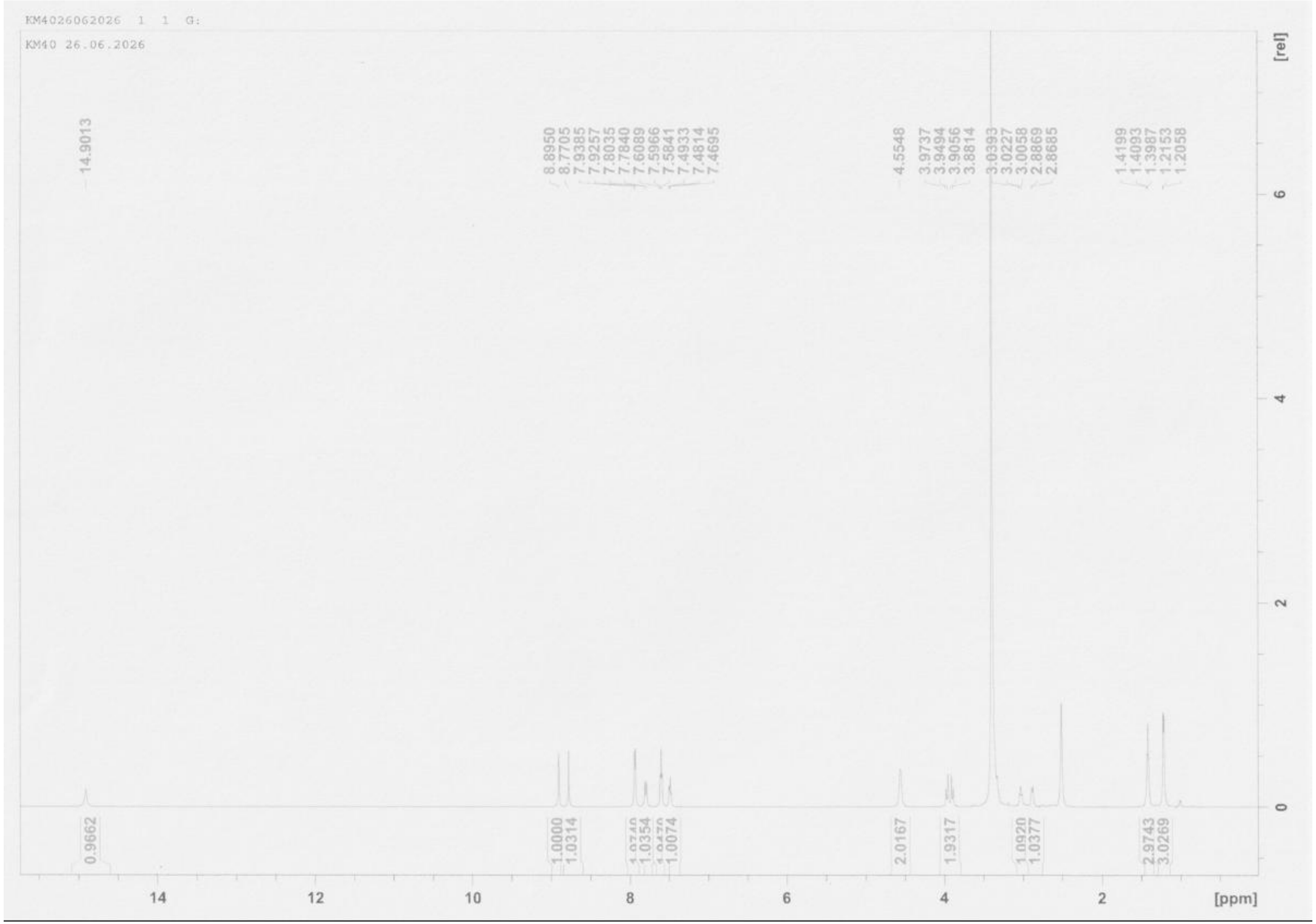

Figure S7. ^{1}H NMR spectra of compound **2**

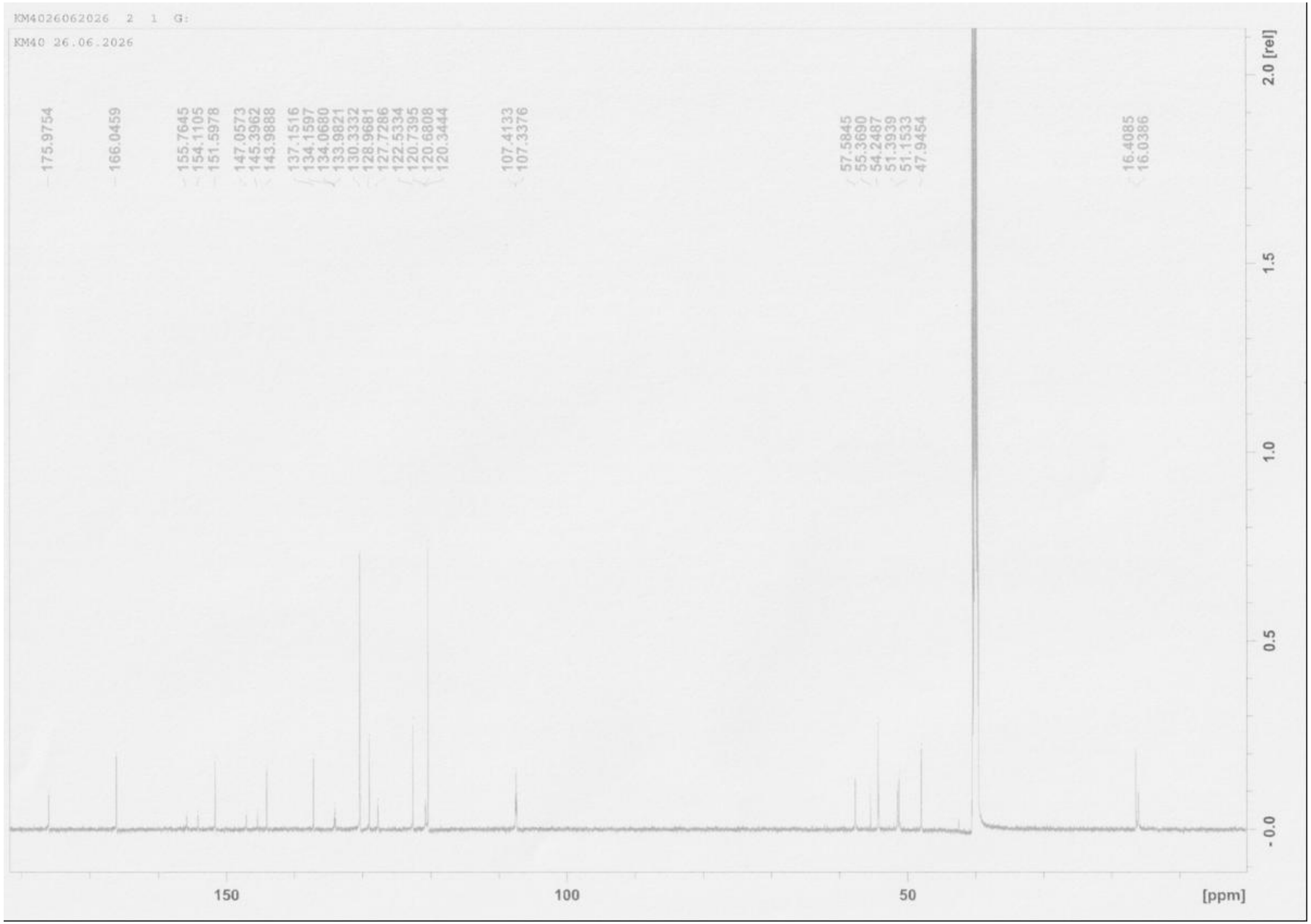

Figure S8. ^{13}C NMR spectra of compound **2**

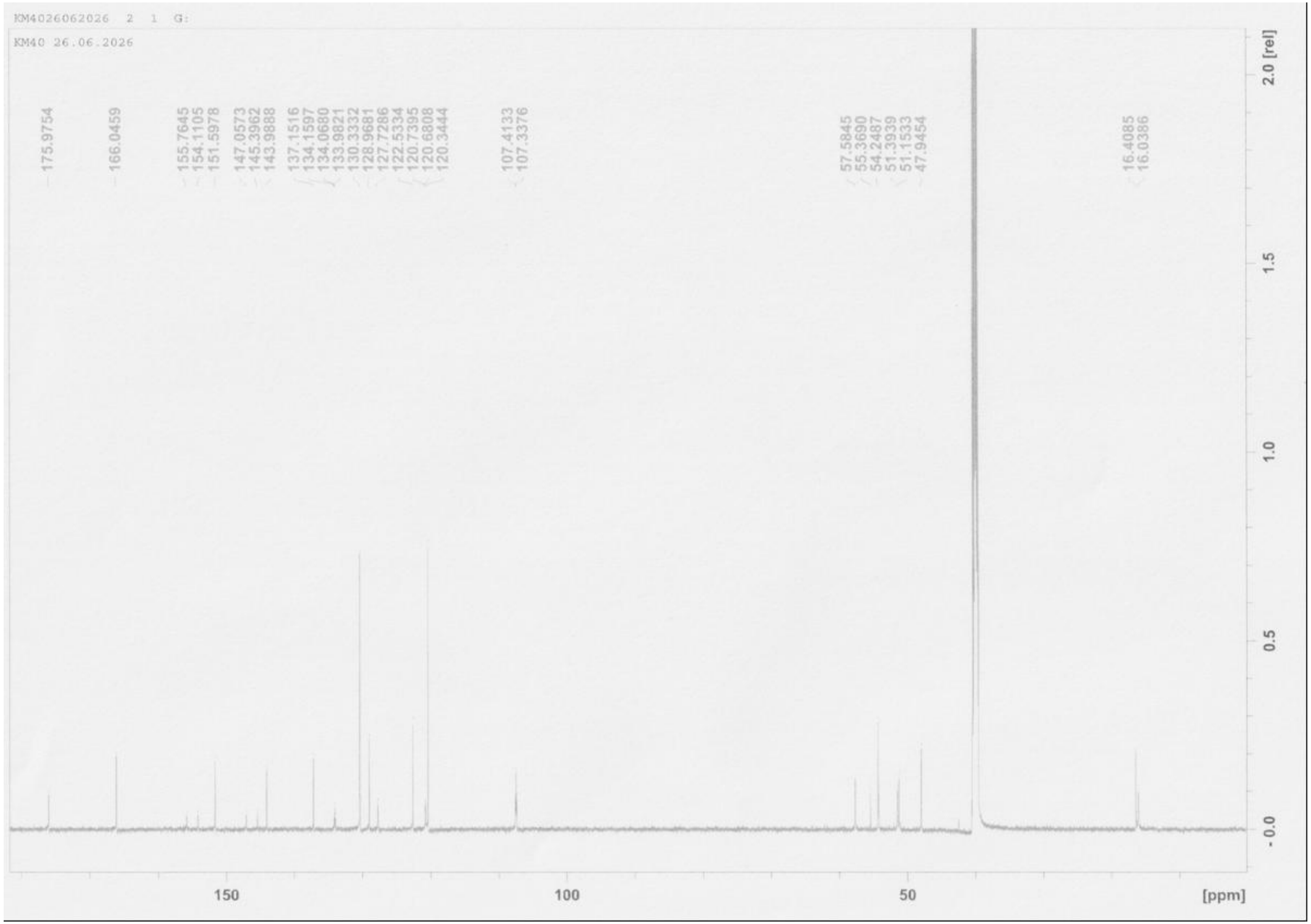

KM4026062026 2 1 G:
KM40 26.06.2026
175.9754
166.0459
155.7645
154.1105
151.5978
147.0573
145.3962
143.9888
137.1516
134.1597
134.0680
133.9821
130.3332
128.9681
127.7286
122.5334
120.7395
120.6808
120.3444
107.4133
107.3376
57.5845
55.3690
54.2487
51.3939
51.1533
47.9454
16.4085
16.0386
2.0 [rel]
1.5
1.0
0.5
0.0
150
100
50
[ppm]

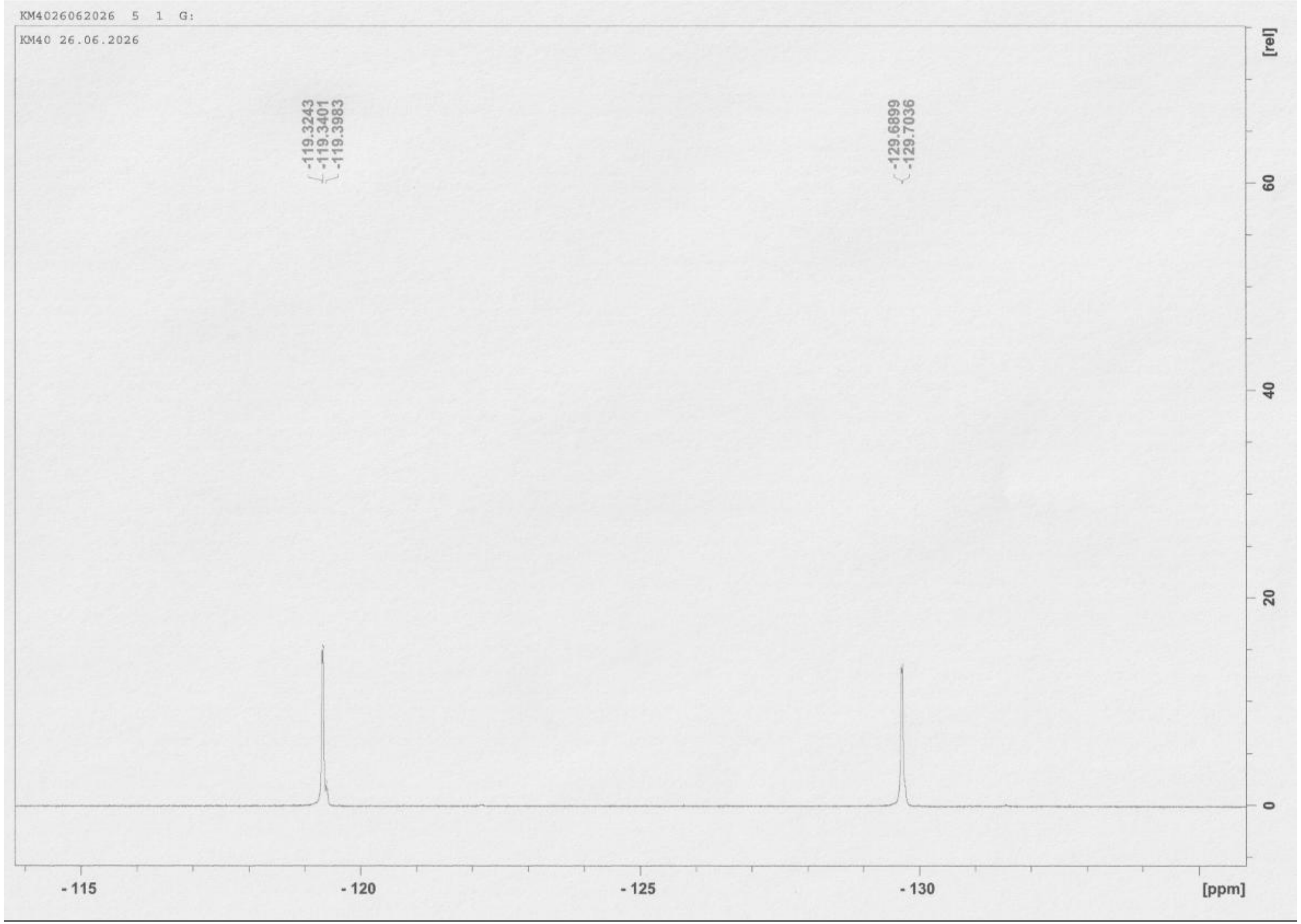

Figure S9. ^{19}F NMR spectra of compound **2**

Compound Spectrum List Report

Analysis Info

Analysis Name	D:\Data\Iome_N_fenyl_a.d	Acquisition Date	4/8/2025 1:53:10 PM
Method	low_mass_positive.m	Operator	KM
Sample Name	Tune_neg_high_post	Instrument	impact II 1825265.10082
Comment			

Acquisition Parameter

Source Type	ESI	Ion Polarity	Positive	Set Nebulizer	0.3 Bar
Focus	Active	Set Capillary	4000 V	Set Dry Heater	200 °C
Scan Begin	100 m/z	Set End Plate Offset	-500 V	Set Dry Gas	3.0 l/min
Scan End	1000 m/z	Set Charging Voltage	2000 V	Set Divert Valve	Source
		Set Corona	0 nA	Set APCI Heater	0 °C

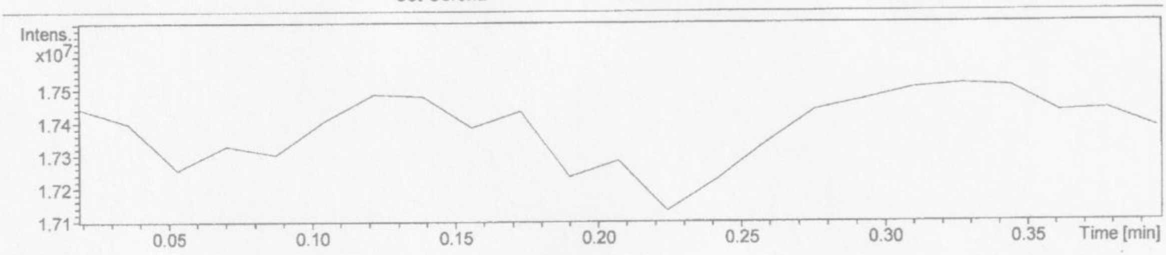

+MS, 0.0-0.4min #1-23

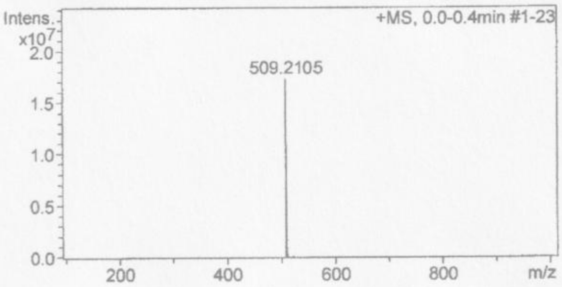

#	m/z	Res.	S/N	I	I %	FWHM
1	509.2105	39815	46533.4	17357274	100.0	0.0128
2	510.2138	38475	12446.6	4655957	26.8	0.0133
3	511.2172	23734	1833.6	688353	4.0	0.0215

Figure S10. HR MS spectra of compound **2**

Chromatogram and Results

Injection Details

Injection Name:	GD1	*Run Time (min):*	10.00
Vial Number:	RA1	*Injection Volume:*	5.00
Injection Type:	Unknown	*Channel:*	UV_VIS_2
Instrument Method:	HPLC 1 seria 0.1	*Bandwidth:*	2
Processing Method:	LC MS	*Dilution Factor:*	1.0000
Injection Date/Time:	03/Jul/26 14:26	*Sample Weight:*	1.0000

Chromatogram

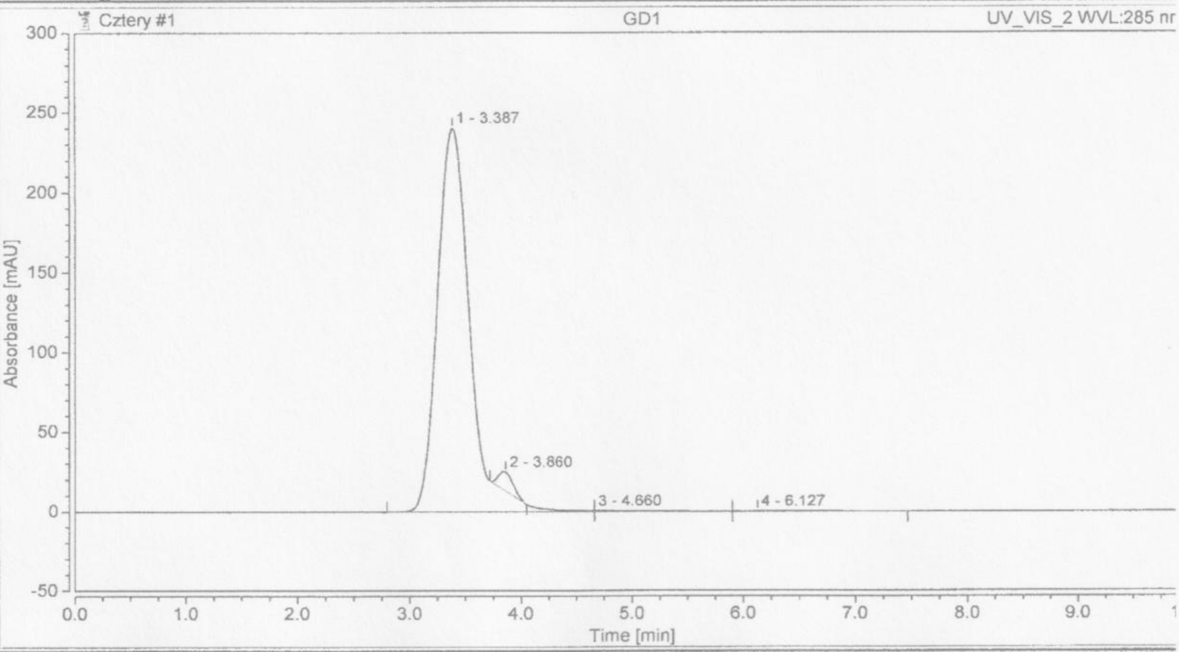

Integration Results

No.	Peak Name	Retention Time min	Area mAU*min	Height mAU	Relative Area %	Relative Height %	Amour n.a.
1		3.387	80.700	240.246	97.26	95.01	n.a.
2		3.860	1.818	11.846	2.19	4.68	n.a.
3		4.660	0.341	0.567	0.41	0.22	n.a.
4		6.127	0.111	0.193	0.13	0.08	n.a.
**Total:**			**82.970**	**252.852**	**100.00**	**100.00**	

Figure S11. HPLC analysis of compound **2**

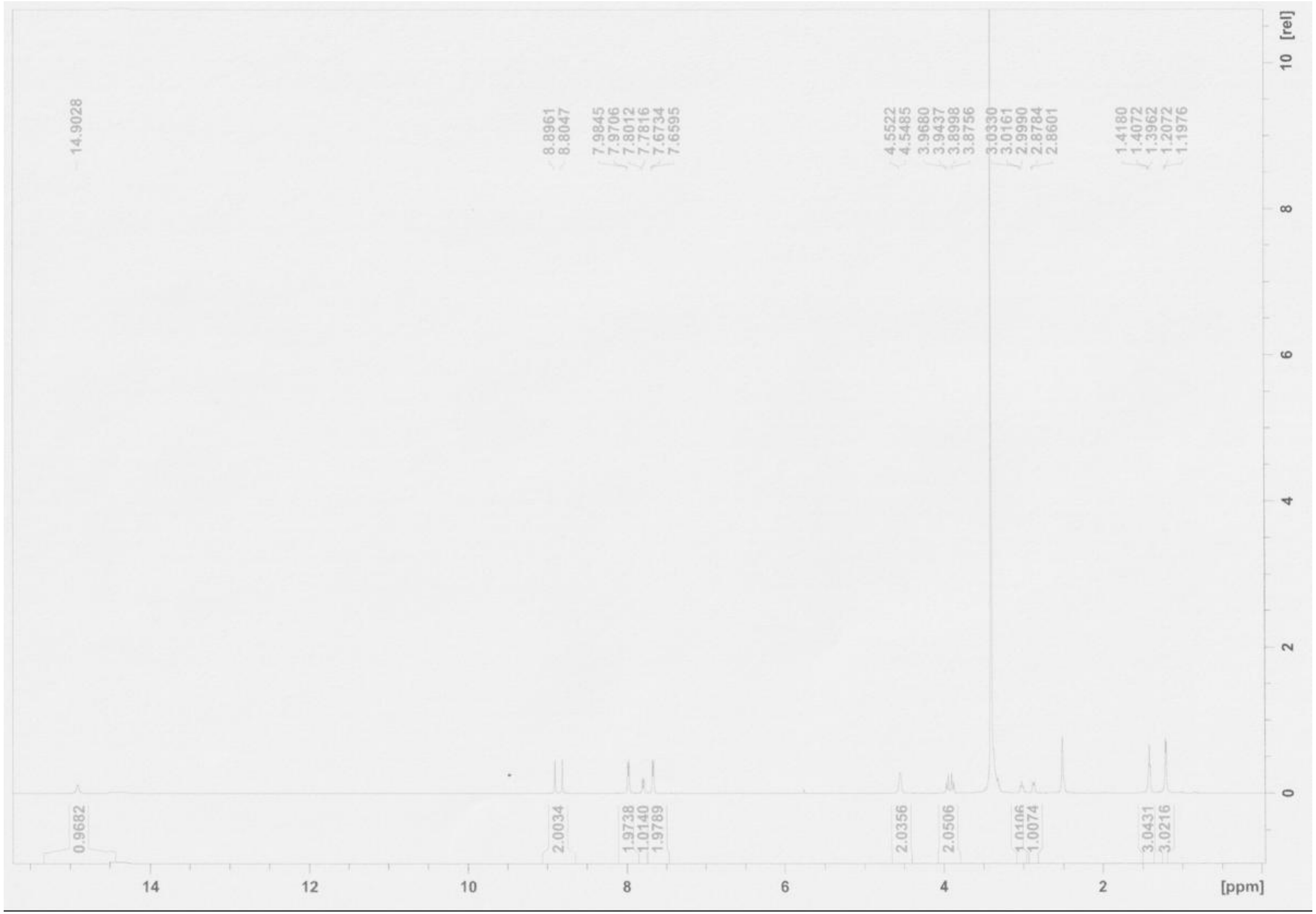

Figure S12. ^{1}H NMR spectra of compound **3**

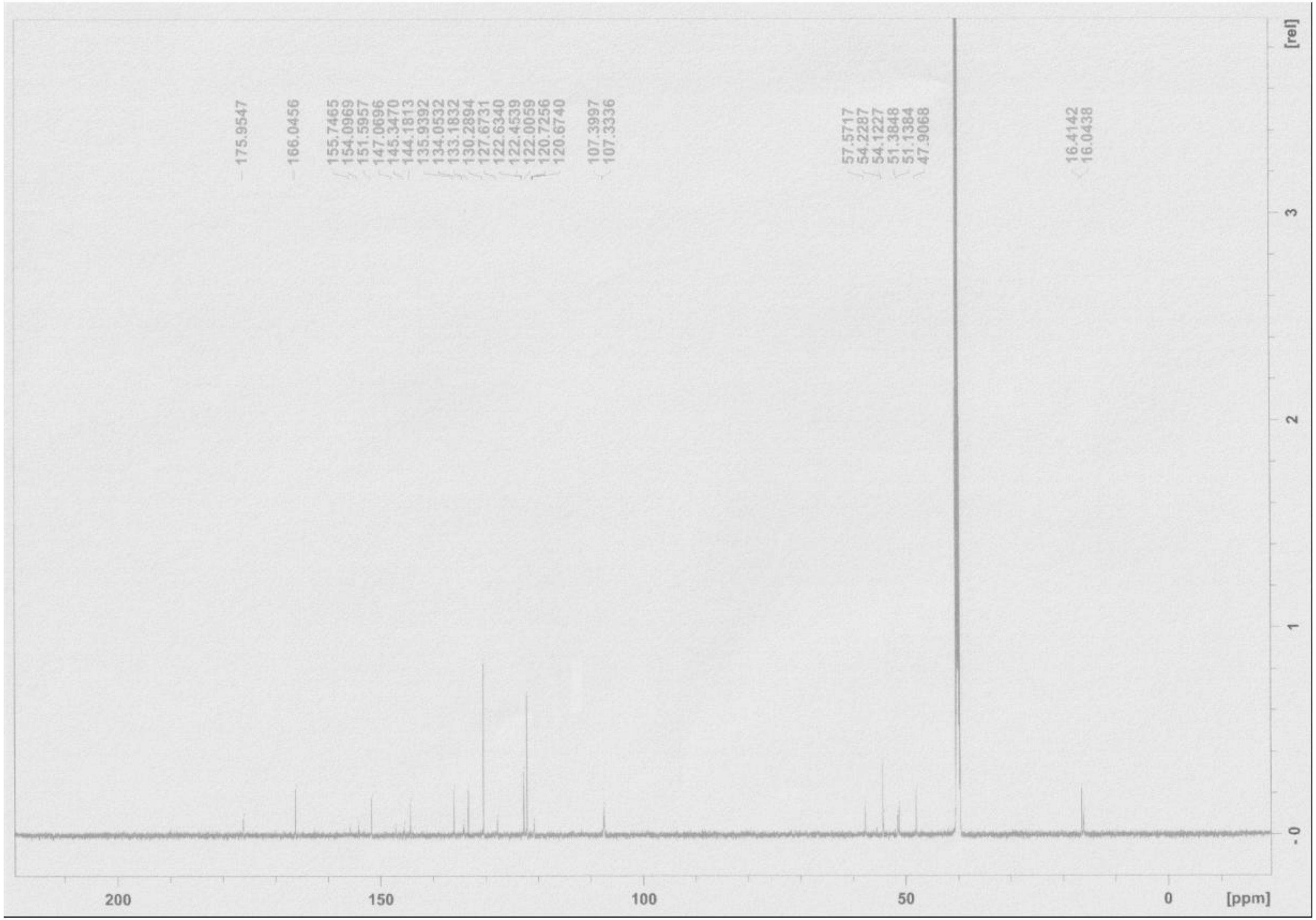

Figure S13. ^{13}C NMR spectra of compound **3**

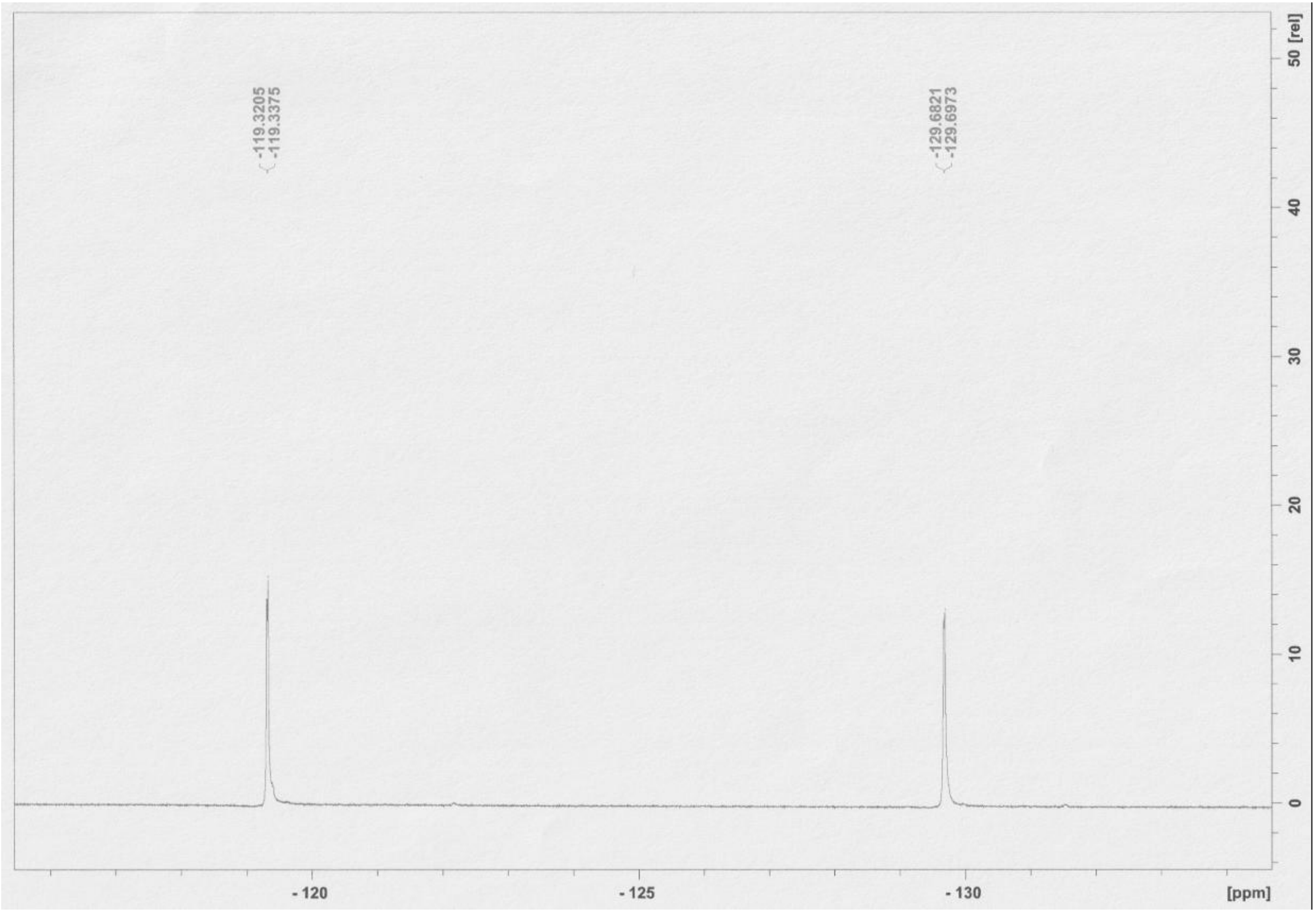

Figure S14. ^{19}F NMR spectra of compound **3**

Compound Spectrum List Report

Analysis Info

Analysis Name	D:\Data\Iome_N_fenyl_Cl.d	Acquisition Date	2/12/2025 3:37:30 PM
Method	low_mass_positive.m	Operator	KM
Sample Name	Tune_neg_high_post	Instrument	impact II 1825265.10082
Comment			

Acquisition Parameter

Source Type	ESI	Ion Polarity	Positive	Set Nebulizer	0.3 Bar
Focus	Active	Set Capillary	4000 V	Set Dry Heater	200 °C
Scan Begin	100 m/z	Set End Plate Offset	-500 V	Set Dry Gas	3.0 l/min
Scan End	1000 m/z	Set Charging Voltage	2000 V	Set Divert Valve	Source
		Set Corona	0 nA	Set APCI Heater	0 °C

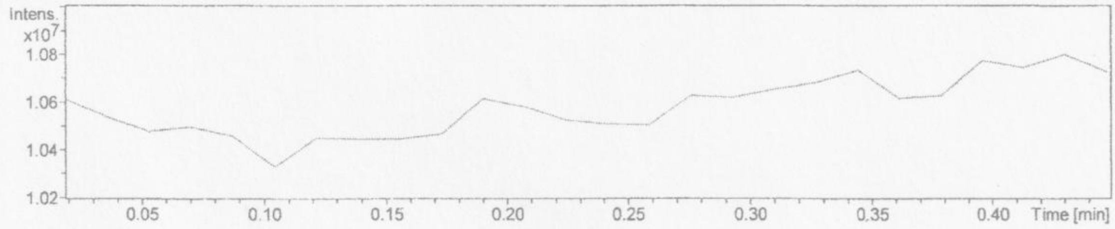

+MS, 0.0-0.4min #1-26

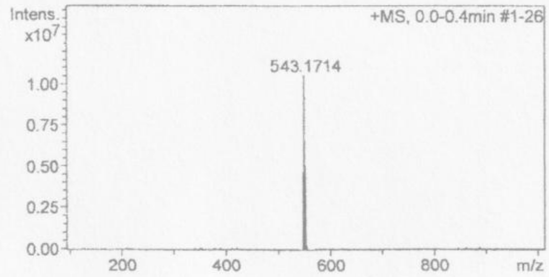

#	m/z	Res.	S/N	I	I %	FWHM
1	543.1714	53417	20914.4	10578486	100.0	0.0154
2	545.1682	38358	7840.5	3789543	32.0	0.0163

Iome_N_fenyl_Cl.d

Bruker Compass DataAnalysis 4.3 printed: 2/12/2025 4:44:22 PM by: KM Page 1 of 1

Figure S15. HR MS spectra of compound **3**

Chromatogram and Results

Injection Details

Injection Name:	GD1	*Run Time (min):*	10.00
Vial Number:	RA1	*Injection Volume:*	5.00
Injection Type:	Unknown	*Channel:*	UV_VIS_2
Instrument Method:	HPLC 1 seria 0.1	*Bandwidth:*	2
Processing Method:	LC MS	*Dilution Factor:*	1.0000
Injection Date/Time:	03/Jul/26 14:15	*Sample Weight:*	1.0000

Chromatogram

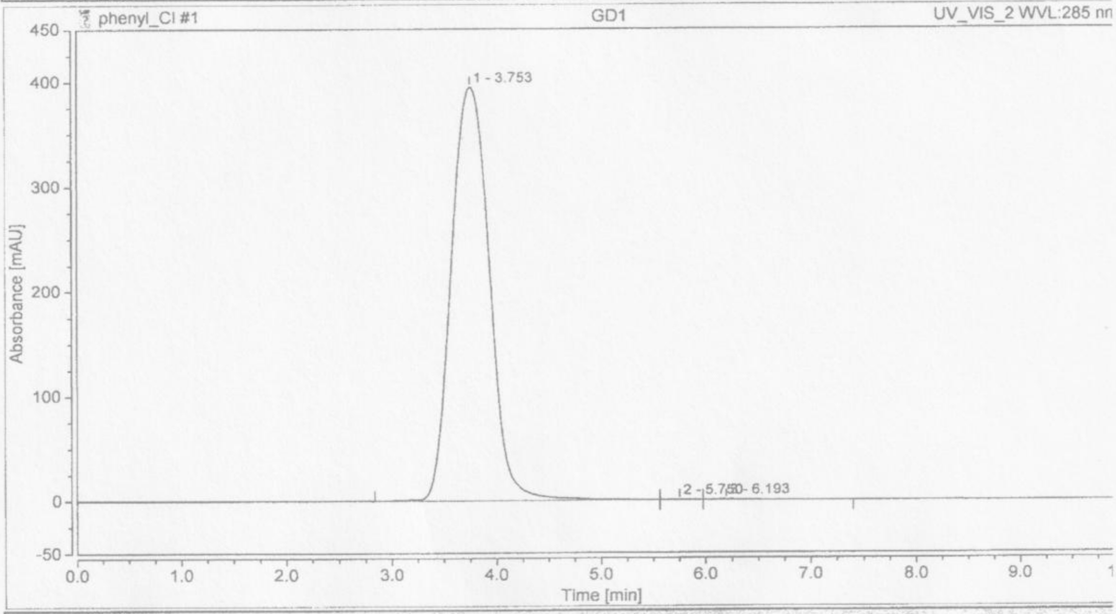

Integration Results

No.	Peak Name	Retention Time min	Area mAU*min	Height mAU	Relative Area %	Relative Height %	Amour n.a.
1		3.753	166.218	395.062	99.67	99.63	n.a.
2		5.750	0.217	0.651	0.13	0.16	n.a.
3		6.193	0.328	0.807	0.20	0.20	n.a.
Total:			166.763	396.521	100.00	100.00	

Figure S16. HPLC analysis of compound **3**

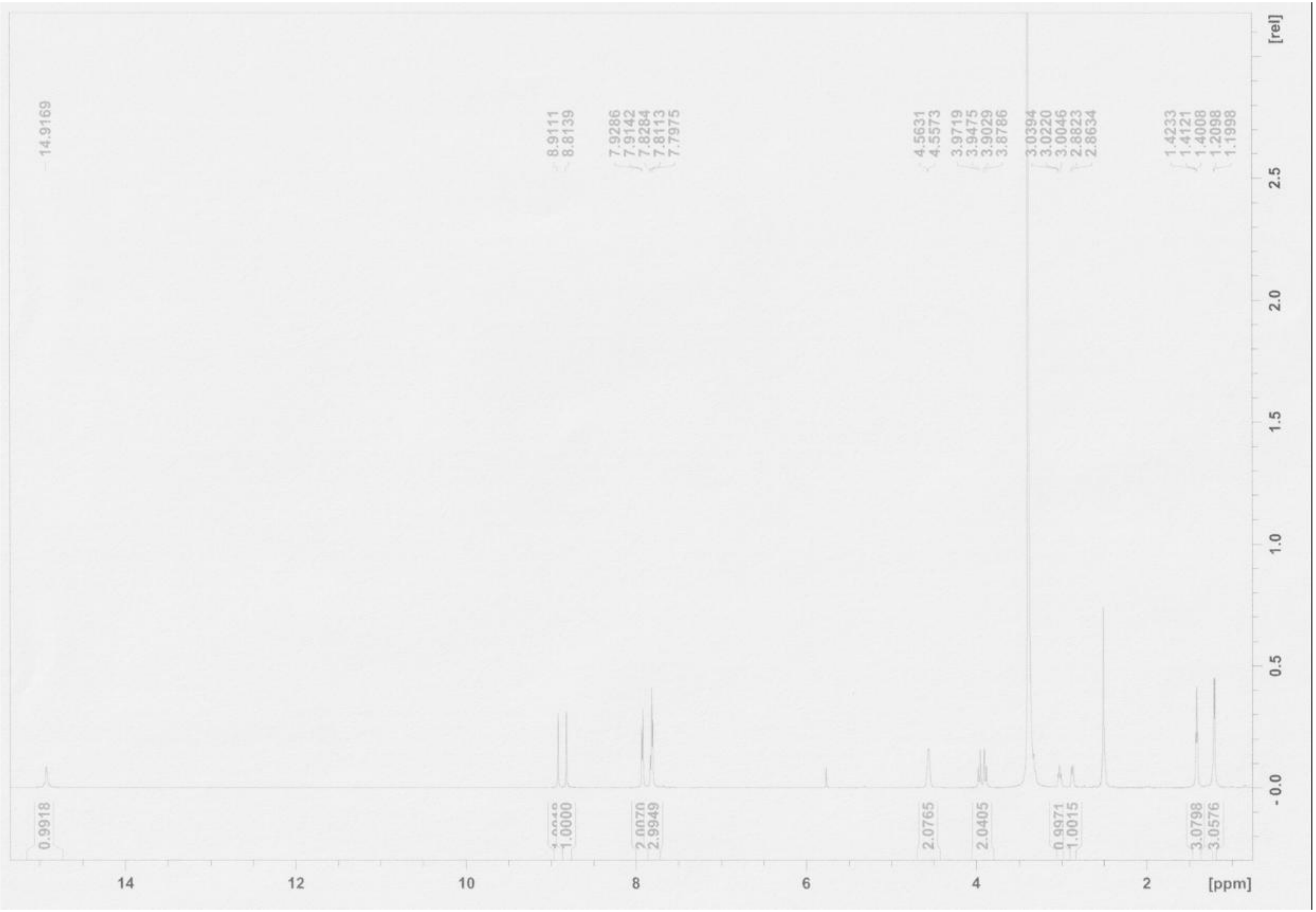

Figure S17. ^{1}H NMR spectra of compound **4**

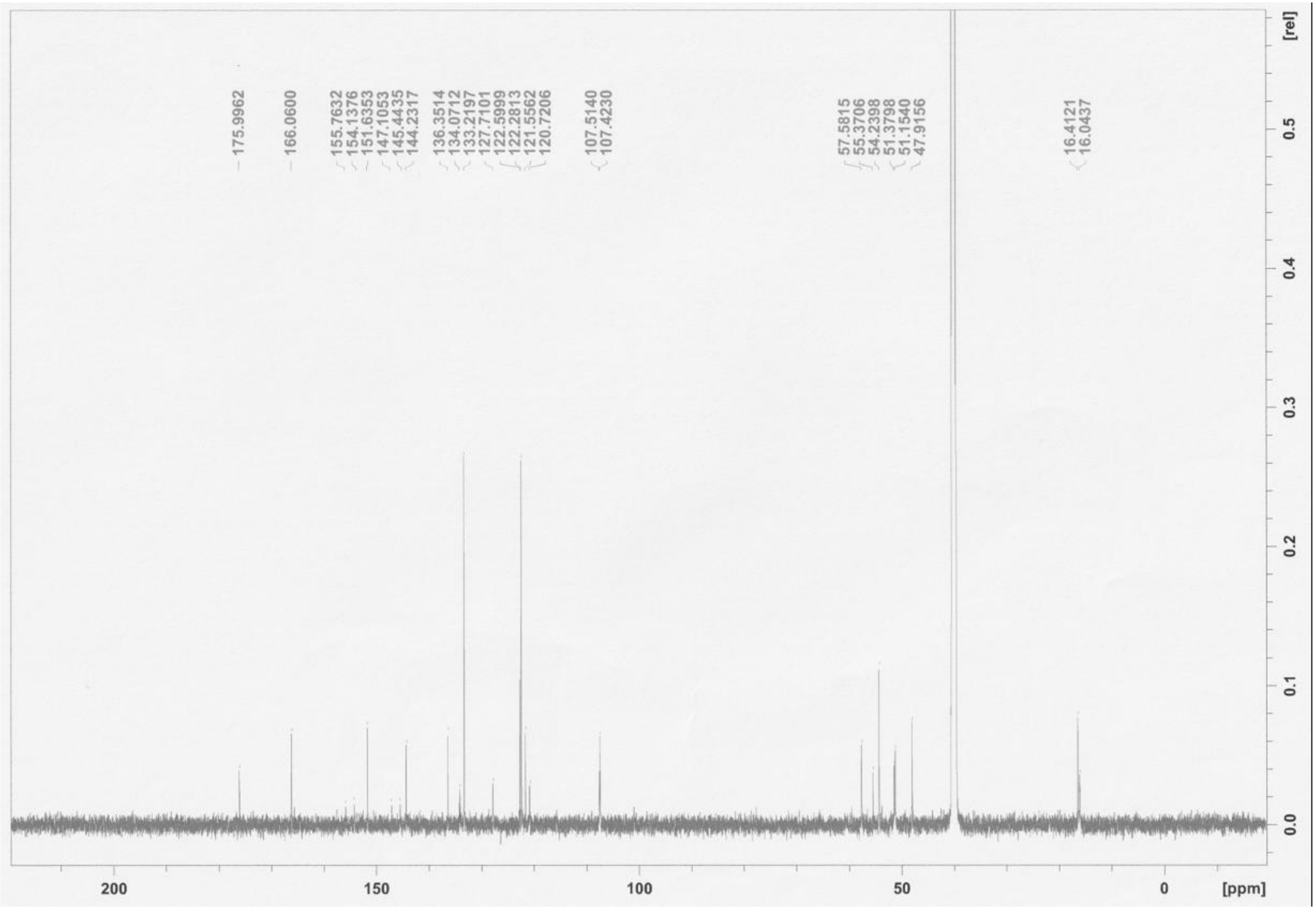

Figure S18. ^{13}C NMR spectra of compound **4**

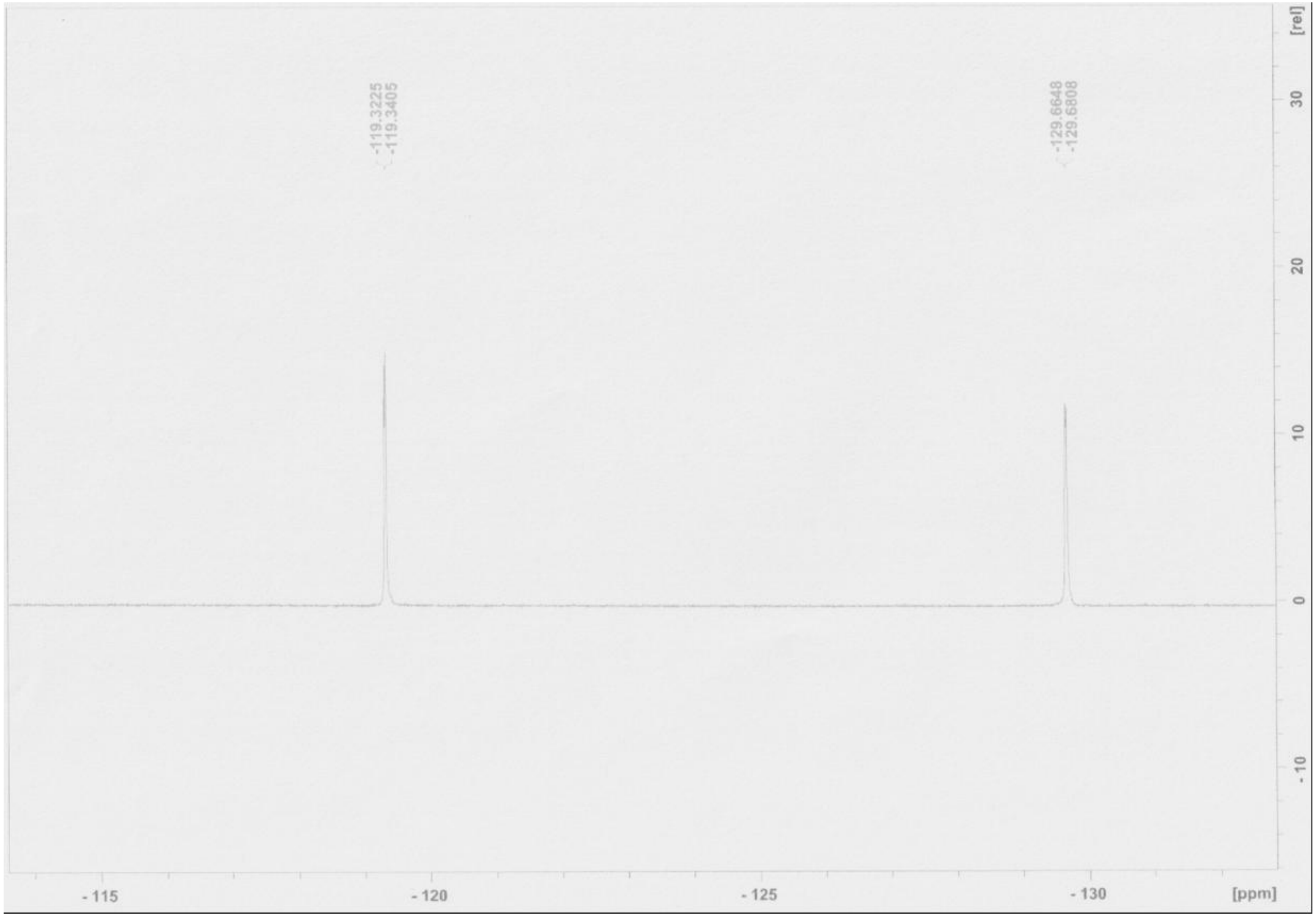

Figure S19. ^{19}F NMR spectra of compound **4**

Compound Spectrum List Report

Analysis Info

Analysis Name	D:\Data\Iome_fenyl_Br.d	Acquisition Date	7/17/2025 11:47:11 AM
Method	low_mass_positive.m	Operator	KM
Sample Name	Tune_neg_high_post	Instrument	impact II 1825265.10082
Comment			

Acquisition Parameter

Source Type	ESI	Ion Polarity	Positive	Set Nebulizer	0.3 Bar
Focus	Active	Set Capillary	4000 V	Set Dry Heater	200 °C
Scan Begin	100 m/z	Set End Plate Offset	-500 V	Set Dry Gas	3.0 l/min
Scan End	1000 m/z	Set Charging Voltage	2000 V	Set Divert Valve	Source
		Set Corona	0 nA	Set APCI Heater	0 °C

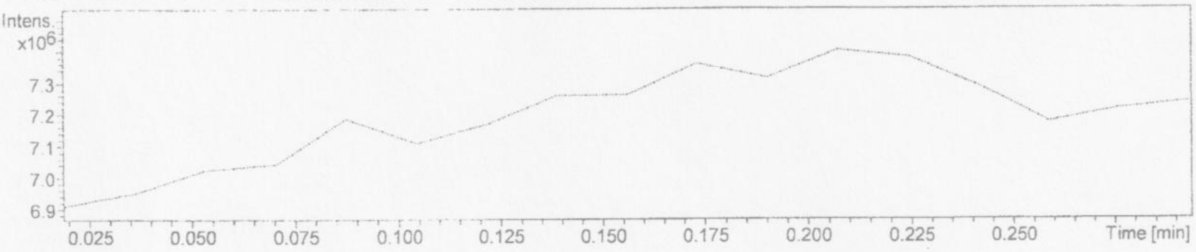

+MS, 0.0-0.3min #1-17

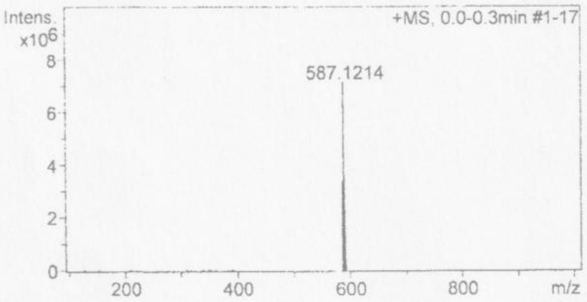

#	m/z	Res.	S/N	I	I %	FWHM
1	587.1214	47856	14158.0	7195616	100.0	0.0172
2	589.1197	46420	13690.0	6979747	96.7	0.0182

Figure S20. HR MS spectra of compound **4**

Chromatogram and Results

Injection Details

Injection Name:	**GD1**	*Run Time (min):*	**10.00**
Vial Number:	**RA2**	*Injection Volume:*	**5.00**
Injection Type:	**Unknown**	*Channel:*	**UV_VIS_2**
Instrument Method:	**HPLC 1 seria 0.1**	*Bandwidth:*	**2**
Processing Method:	**LC MS**	*Dilution Factor:*	**1.0000**
Injection Date/Time:	**03/Jul/26 14:38**	*Sample Weight:*	**1.0000**

Chromatogram

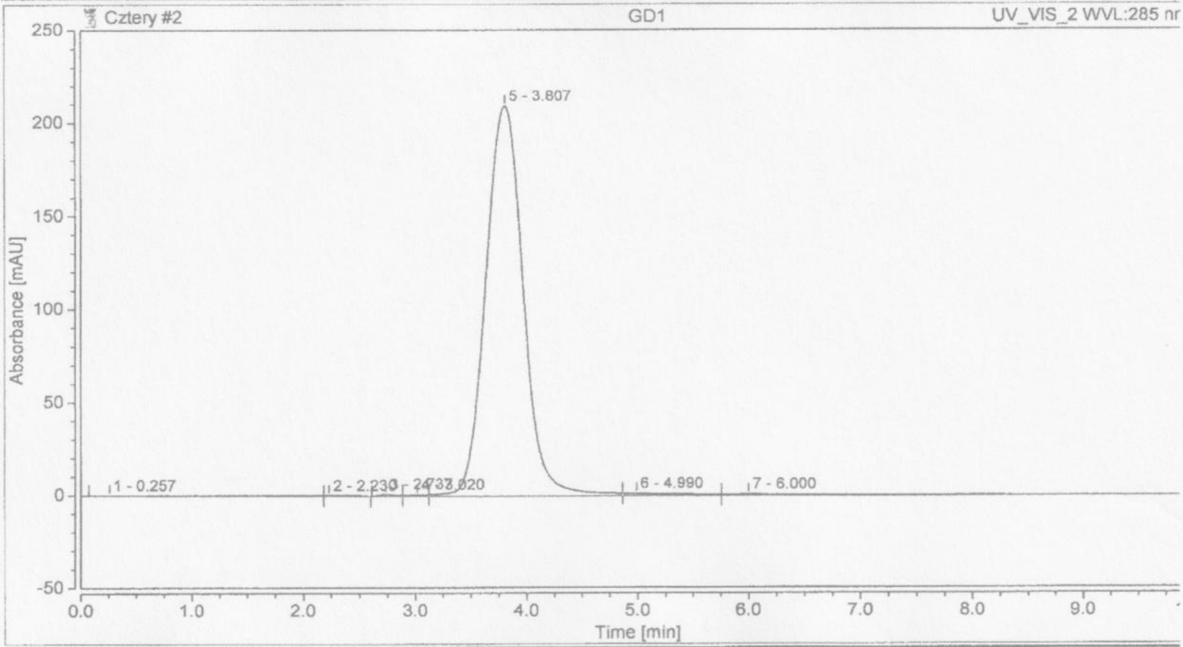

Integration Results

No.	Peak Name	Retention Time min	Area mAU*min	Height mAU	Relative Area %	Relative Height %	Amour n.a.
1		0.257	0.387	0.068	0.46	0.03	n.a.
2		2.230	0.133	0.349	0.16	0.16	n.a.
3		2.737	0.169	1.077	0.20	0.50	n.a.
4		3.020	0.164	0.735	0.20	0.34	n.a.
5		3.807	81.444	209.539	97.01	98.07	n.a.
6		4.990	0.760	1.227	0.91	0.57	n.a.
7		6.000	0.897	0.674	1.07	0.32	n.a.
**Total:**			**83.954**	**213.668**	**100.00**	**100.00**	

Figure S21. HPLC analysis of compound **4**

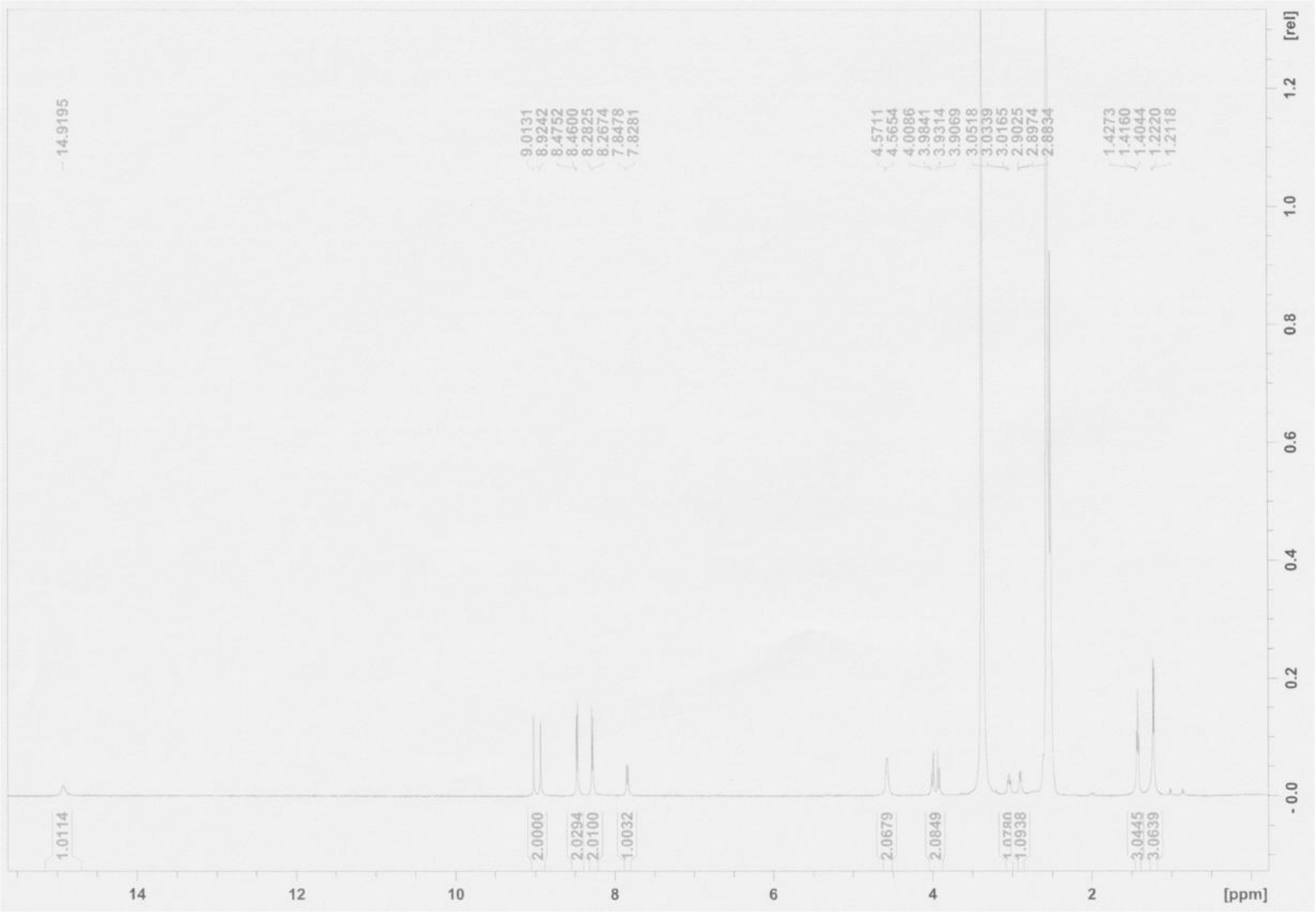

Figure S22. ^1H NMR spectra of compound **5**

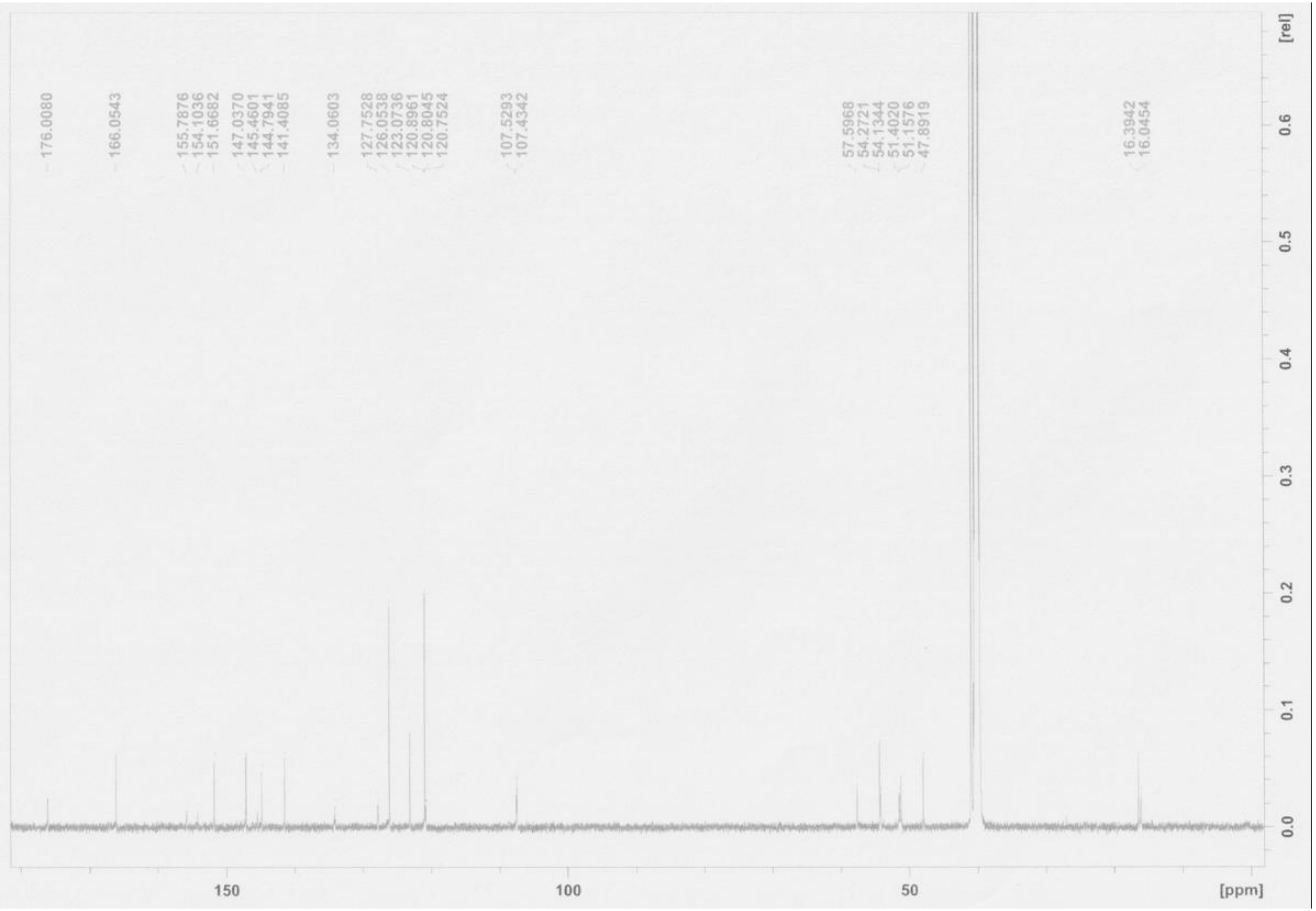

Figure S23. ^{13}C NMR spectra of compound **5**

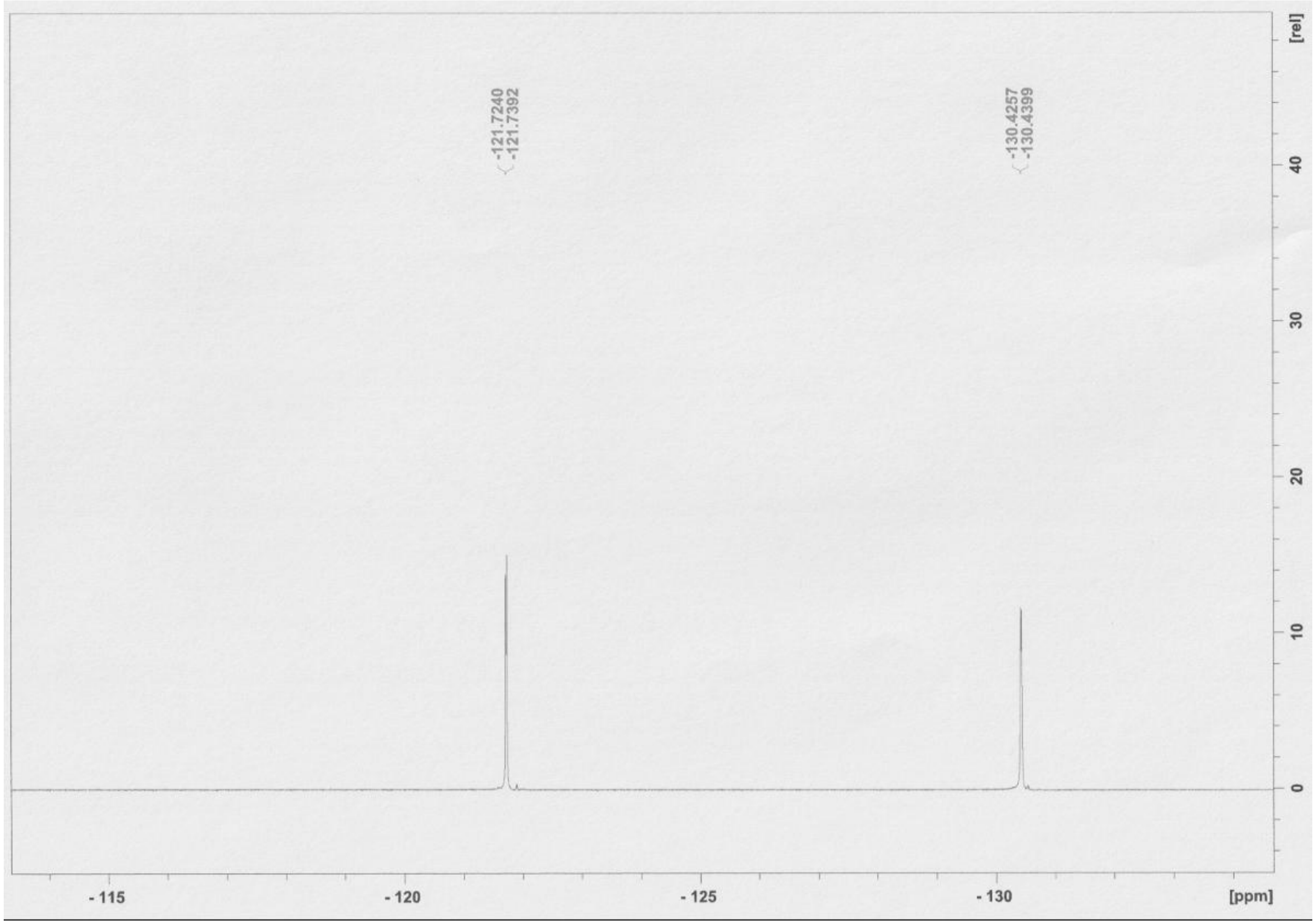

Figure S24. ^{19}F NMR spectra of compound **5**

Compound Spectrum List Report

Analysis Info

Analysis Name	D:\Data\lome_fenyl_pNO2.d	Acquisition Date	4/9/2025 12:13:53 PM
Method	low_mass_positive.m	Operator	KM
Sample Name	Tune_neg_high_post	Instrument	impact II 1825265.10082
Comment			

Acquisition Parameter

Source Type	ESI	Ion Polarity	Positive	Set Nebulizer	0.3 Bar
Focus	Active	Set Capillary	4000 V	Set Dry Heater	200 °C
Scan Begin	100 m/z	Set End Plate Offset	-500 V	Set Dry Gas	3.0 l/min
Scan End	1000 m/z	Set Charging Voltage	2000 V	Set Divert Valve	Source
		Set Corona	0 nA	Set APCI Heater	0 °C

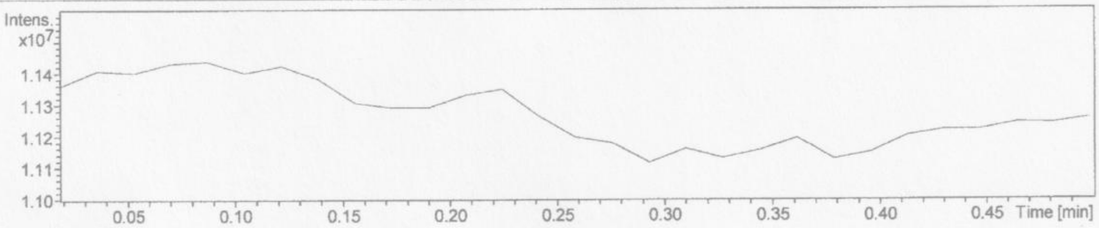

+MS, 0.0-0.5min #1-29

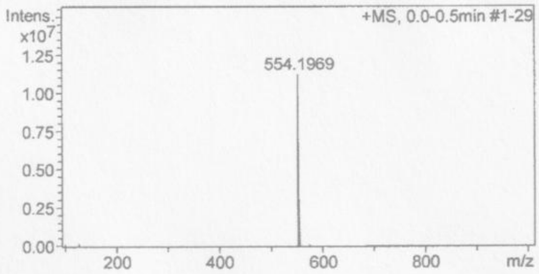

#	m/z	Res.	S/N	I	I %	FWHM
1	554.1969	43802	30754.7	11242395	100.0	0.0127
2	555.1991	33402	8280.7	3029091	26.9	0.0166
3	556.2013	21999	1431.7	525054	4.7	0.0253

lome_fenyl_pNO2.d
Bruker Compass DataAnalysis 4.3 printed: 4/9/2025 12:15:11 PM by: KM Page 1 of 1

Figure S25. HR MS spectra of compound **5**

Chromatogram and Results

Injection Details

Injection Name:	**GD1**	*Run Time (min):*	**10.00**
Vial Number:	**RA3**	*Injection Volume:*	**5.00**
Injection Type:	**Unknown**	*Channel:*	**UV_VIS_2**
Instrument Method:	**HPLC 1 seria 0.1**	*Bandwidth:*	**2**
Processing Method:	**LC MS**	*Dilution Factor:*	**1.0000**
Injection Date/Time:	**03/Jul/26 14:50**	*Sample Weight:*	**1.0000**

Chromatogram

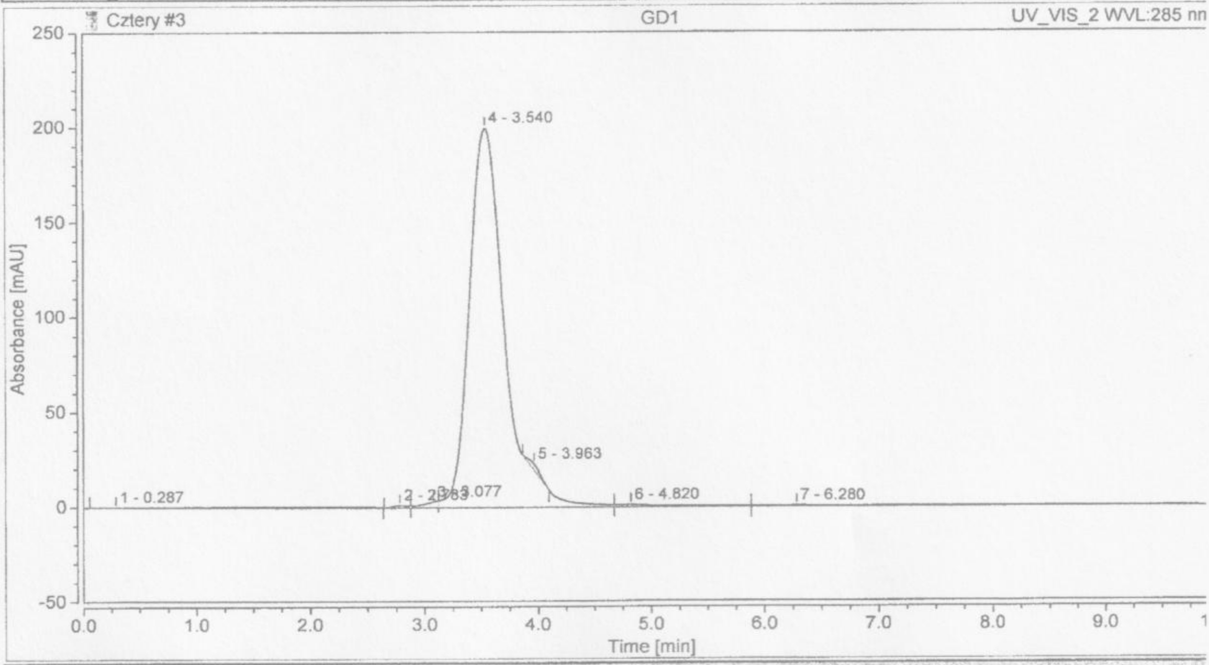

Integration Results

No.	Peak Name	Retention Time min	Area mAU*min	Height mAU	Relative Area %	Relative Height %	Amoun n.a.
1		0.287	0.637	0.080	0.85	0.04	n.a.
2		2.783	0.198	1.341	0.26	0.64	n.a.
3		3.077	0.511	3.052	0.68	1.46	n.a.
4		3.540	71.555	199.965	95.36	95.41	n.a.
5		3.963	0.400	3.390	0.53	1.62	n.a.
6		4.820	0.867	1.281	1.16	0.61	n.a.
7		6.280	0.872	0.469	1.16	0.22	n.a.
**Total:**			**75.040**	**209.579**	**100.00**	**100.00**	

Figure S26. HPLC analysis of compound **5**

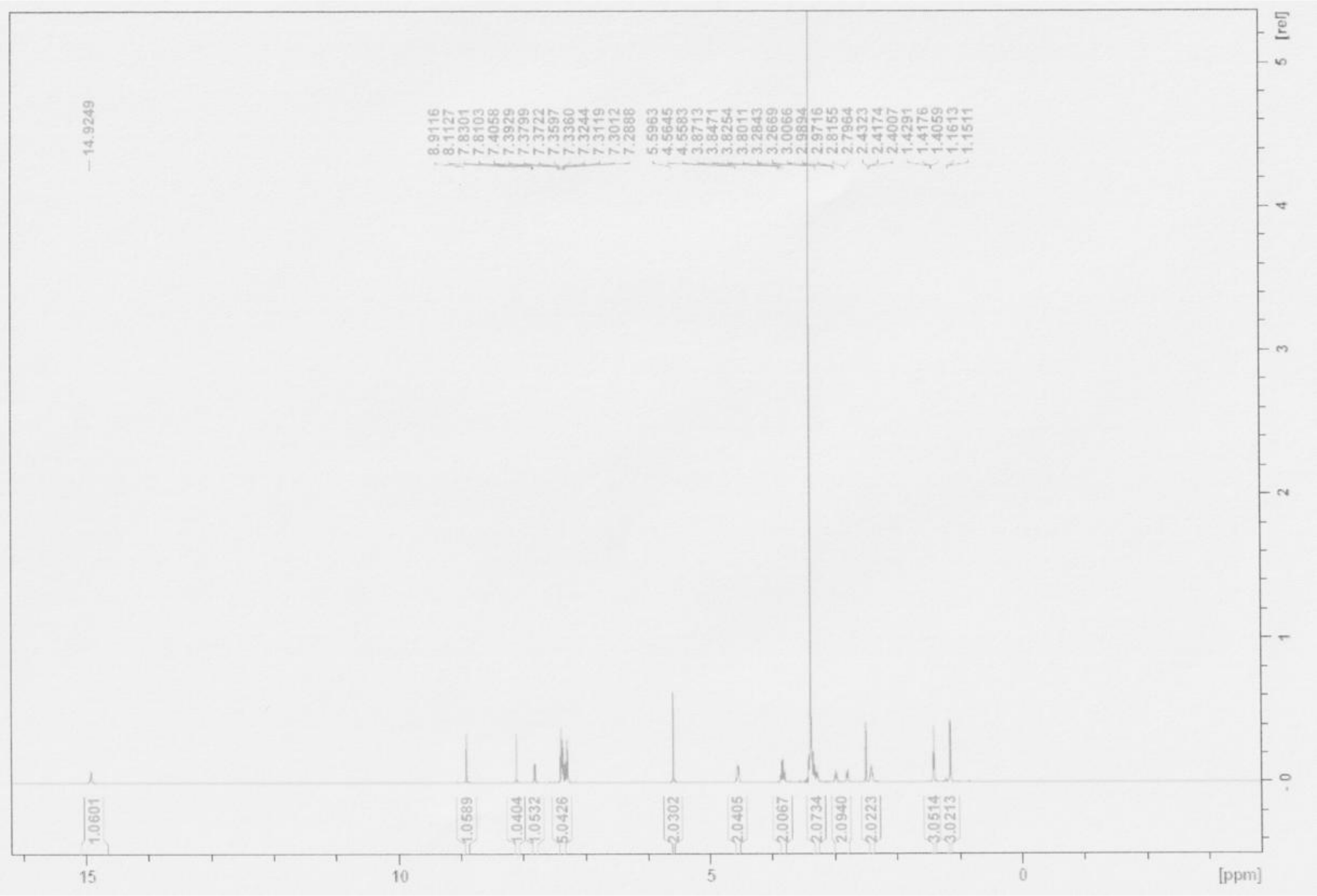

Figure S27. ^{1}H NMR spectra of compound **6**

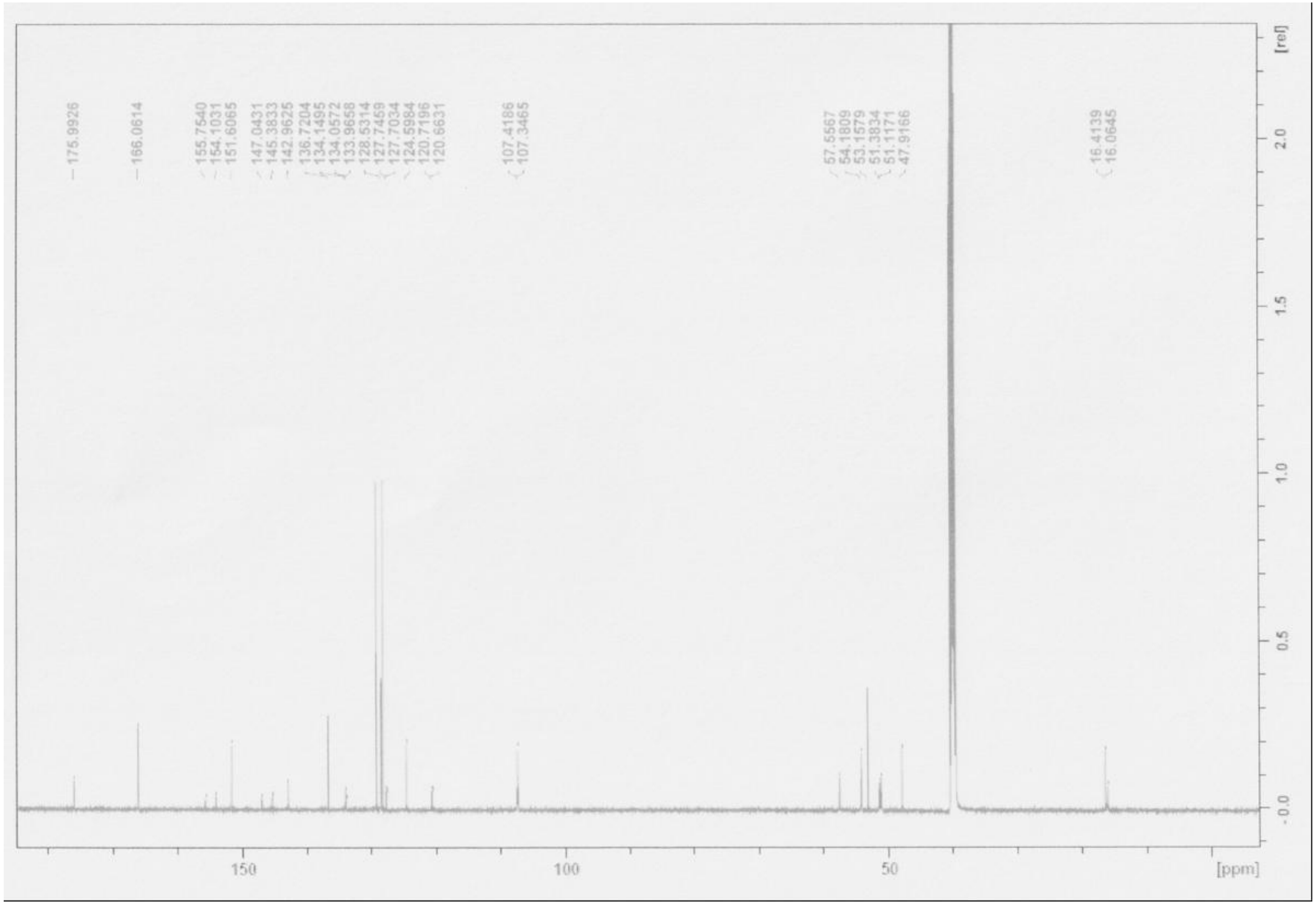

Figure S28. ^{13}C NMR spectra of compound **6**

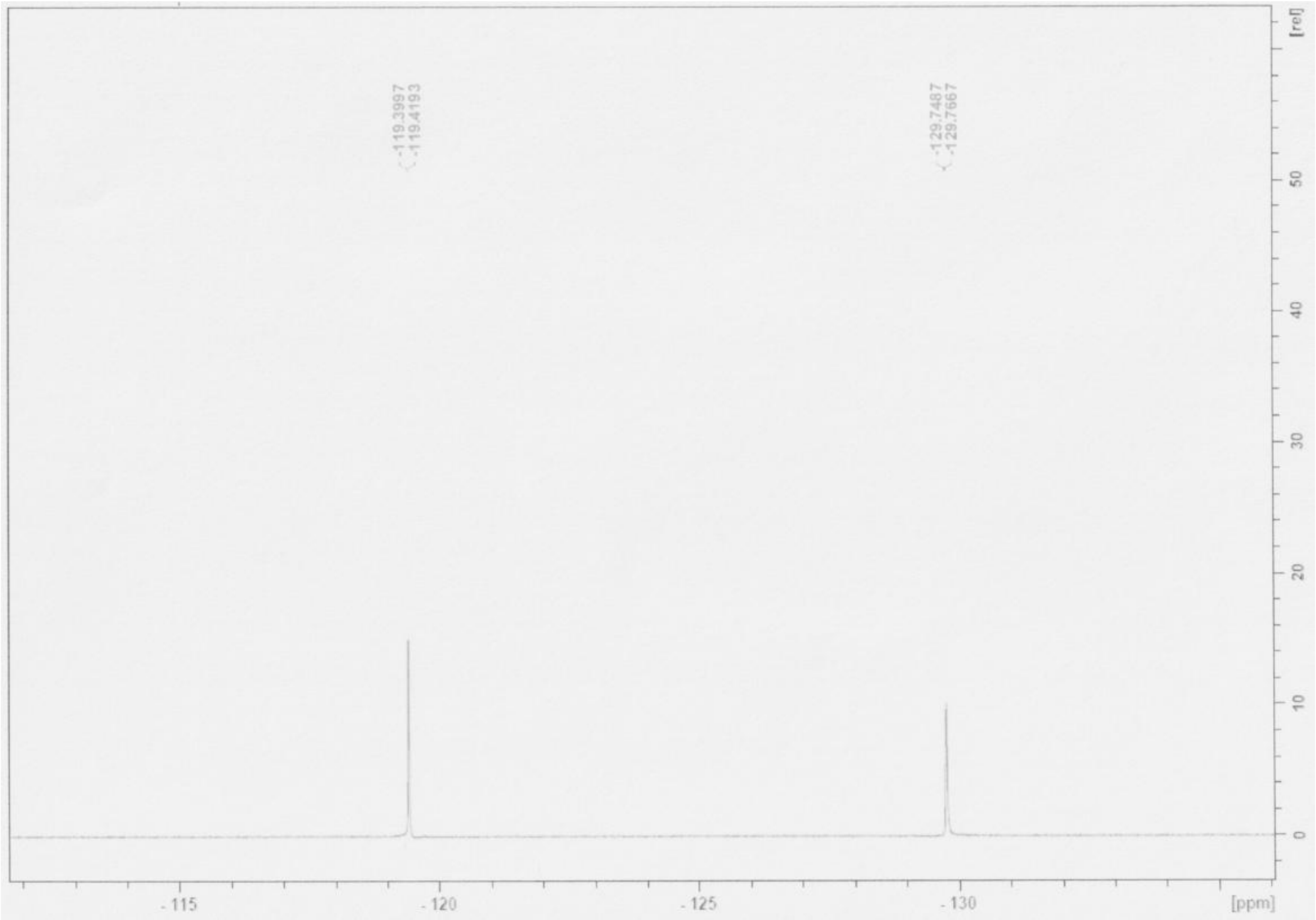

Figure S29. ^{19}F NMR spectra of compound **6**

Compound Spectrum List Report

Analysis Info

Analysis Name	D:\Data\lome_N_benzyl_2.d	Acquisition Date	4/8/2025 12:50:56 PM
Method	low_mass_positive.m	Operator	KM
Sample Name	Tune_neg_high_post	Instrument	impact II 1825265.10082
Comment			

Acquisition Parameter

Source Type	ESI	Ion Polarity	Positive	Set Nebulizer	0.3 Bar
Focus	Active	Set Capillary	4000 V	Set Dry Heater	200 °C
Scan Begin	100 m/z	Set End Plate Offset	-500 V	Set Dry Gas	3.0 l/min
Scan End	1000 m/z	Set Charging Voltage	2000 V	Set Divert Valve	Source
		Set Corona	0 nA	Set APCI Heater	0 °C

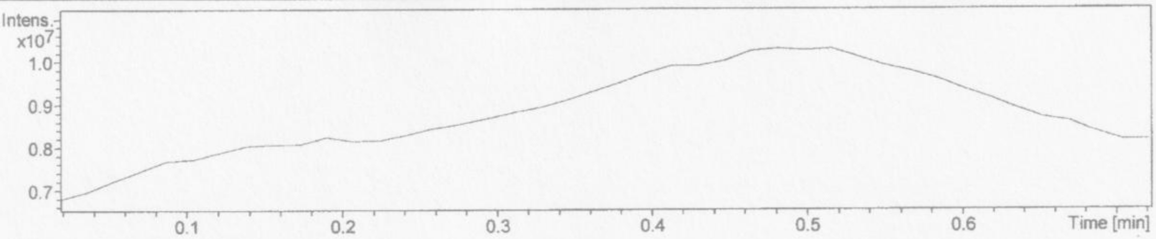

+MS, 0.0-0.7min #1-42

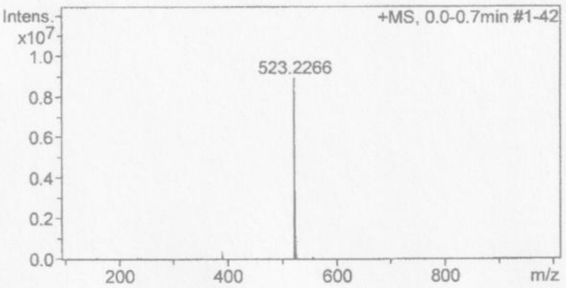

#	m/z	Res.	S/N	I	I %	FWHM
1	523.2266	40666	28665.1	8887948	100.0	0.0129
2	524.2289	28692	7800.6	2425937	27.3	0.0183
3	525.2313	19973	1318.6	410658	4.6	0.0263

Figure S30. HR MS spectra of compound **6**

Instrument:HPLC Sequence:dwa_1 Pa

Chromatogram and Results

Injection Details

Injection Name:	GD1	*Run Time (min):*	10.00
Vial Number:	RA1	*Injection Volume:*	5.00
Injection Type:	Unknown	*Channel:*	UV_VIS_2
Calibration Level:		*Wavelength:*	265.0
Instrument Method:	HPLC 1 seria 0.1	*Bandwidth:*	2
Processing Method:	LC MS	*Dilution Factor:*	1.0000
Injection Date/Time:	10/Jul/26 13:26	*Sample Weight:*	1.0000

Chromatogram

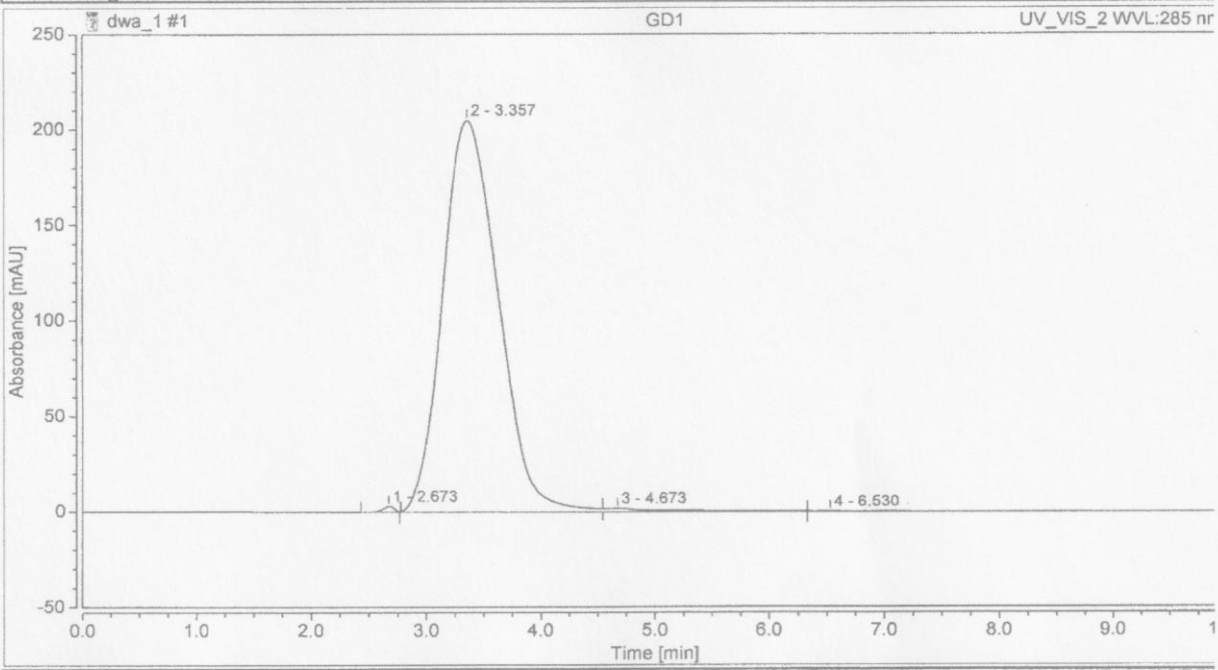

Integration Results

No.	Peak Name	Retention Time min	Area mAU*min	Height mAU	Relative Area %	Relative Height %	Amoun n.a.
1		2.673	0.346	3.035	0.30	1.44	n.a.
2		3.357	115.235	204.924	98.41	97.56	n.a.
3		4.673	1.170	1.810	1.00	0.86	n.a.
4		6.530	0.350	0.275	0.30	0.13	n.a.
**Total:**			**117.101**	**210.045**	**100.00**	**100.00**	

Default DAD/Integration

Chromeleon (
Version 7.

Figure S31. HPLC analysis of compound **6**

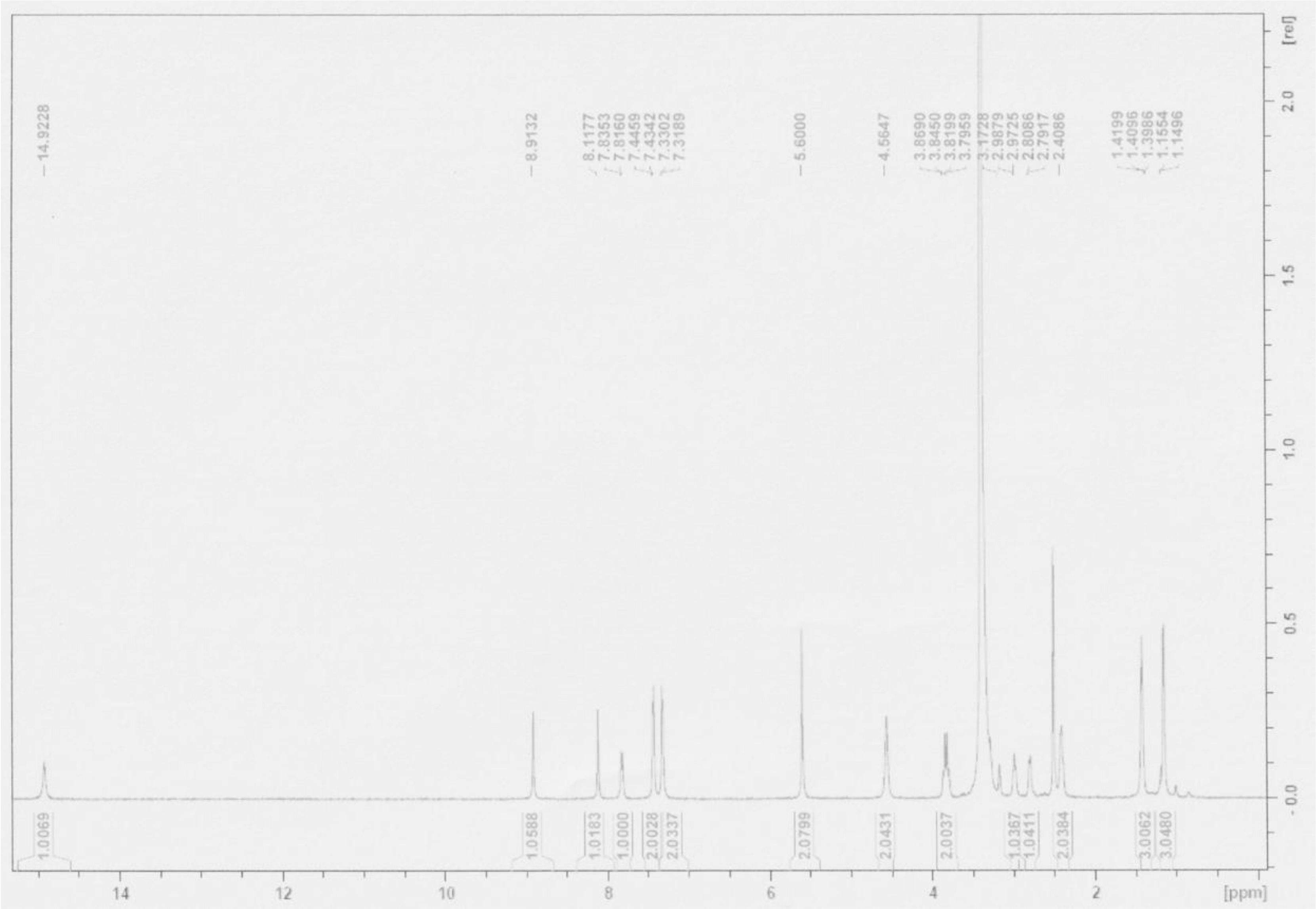

Figure S32. ^{1}H NMR spectra of compound **7**

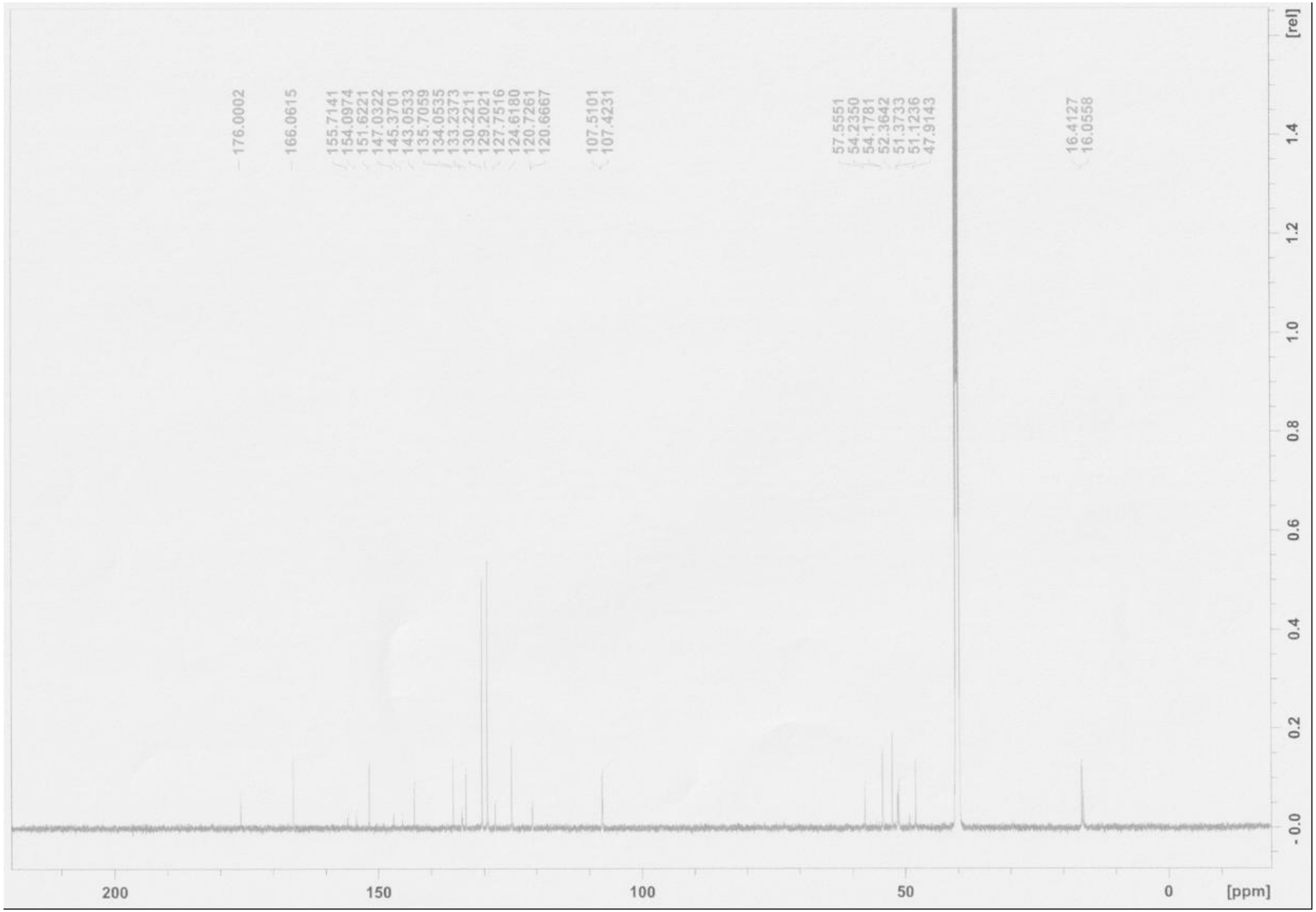

Figure S33. ^{13}C NMR spectra of compound **7**

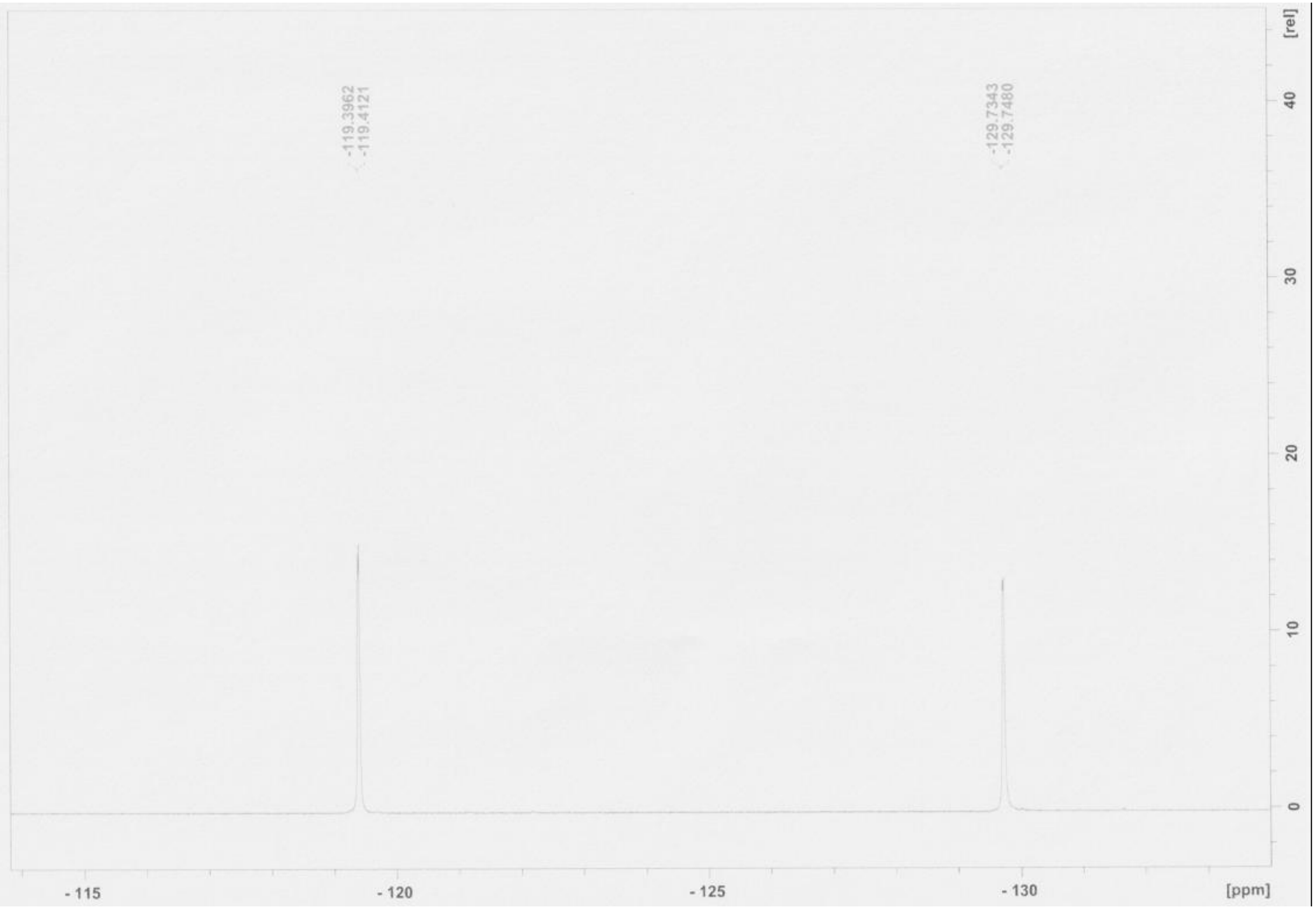

Figure S34. ^{19}F NMR spectra of compound **7**

Compound Spectrum List Report

Analysis Info

Analysis Name D:\Data\test6.d
Method low_mass_positive.m
Sample Name Tune_neg_high_post
Comment

Acquisition Date 4/16/2025 1:36:06 PM

Operator KM
Instrument impact II 1825265.10082

Acquisition Parameter

Source Type	ESI	Ion Polarity	Positive	Set Nebulizer	0.3 Bar
Focus	Active	Set Capillary	4000 V	Set Dry Heater	200 °C
Scan Begin	100 m/z	Set End Plate Offset	-500 V	Set Dry Gas	3.0 l/min
Scan End	1000 m/z	Set Charging Voltage	2000 V	Set Divert Valve	Source
		Set Corona	0 nA	Set APCI Heater	0 °C

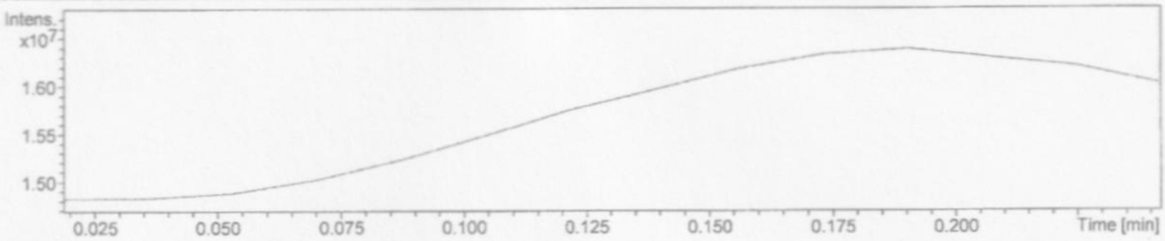

+MS, 0.0-0.2min #1-14

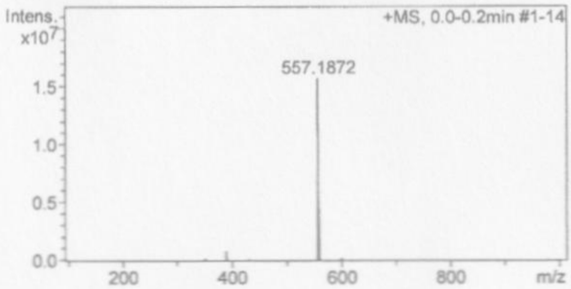

#	m/z	Res.	S/N	I	I %	FWHM
1	557.1872	43002	41377.9	15692606	100.0	0.0130
2	559.1845	39409	13491.3	5124424	32.7	0.0142

P

Instrument:HPLC Sequence:benzyl_Cl

Page 1 of 1

Chromatogram and Results

Injection Details

Injection Name:	**GD1**	*Run Time (min):*	**10.00**
Vial Number:	**RA1**	*Injection Volume:*	**5.00**
Injection Type:	**Unknown**	*Channel:*	**UV_VIS_2**
Instrument Method:	**HPLC 1 seria 0.1**	*Bandwidth:*	**2**
Processing Method:	**LC MS**	*Dilution Factor:*	**1.0000**
Injection Date/Time:	**13/Jul/26 13:25**	*Sample Weight:*	**1.0000**

Chromatogram

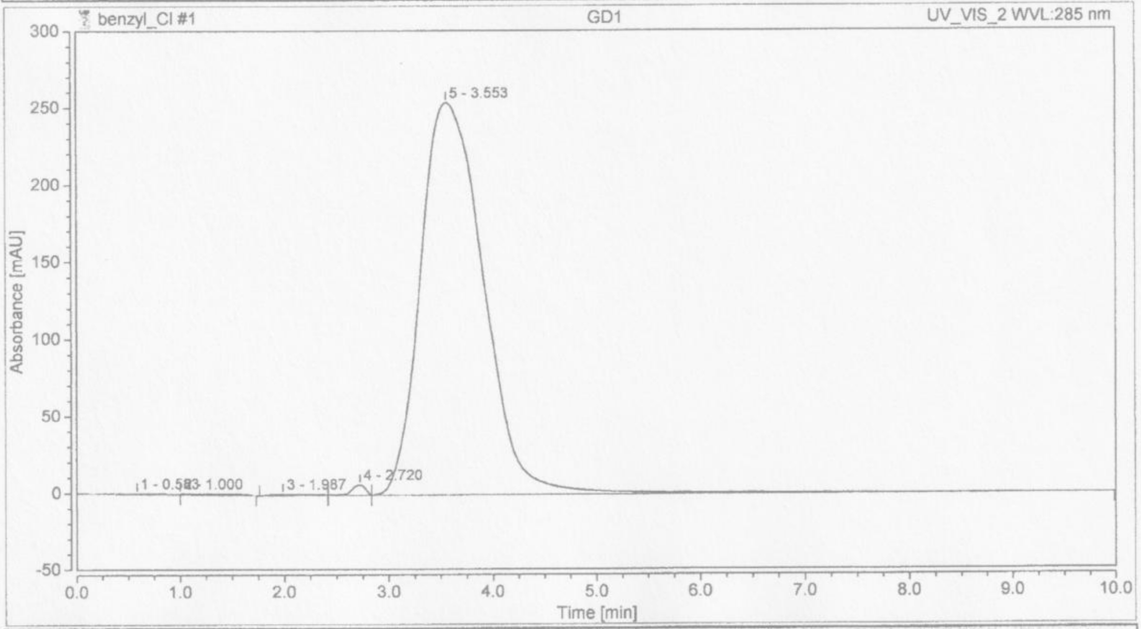

Integration Results

No.	Peak Name	Retention Time min	Area mAU*min	Height mAU	Relative Area %	Relative Height %	Amount n.a.
1		0.583	0.053	0.106	0.03	0.04	n.a.
2		1.000	0.773	0.587	0.42	0.22	n.a.
3		1.987	0.380	0.856	0.21	0.33	n.a.
4		2.720	1.049	6.615	0.58	2.52	n.a.
5		3.553	179.648	254.449	98.76	96.89	n.a.
**Total:**			**181.902**	**262.614**	**100.00**	**100.00**	

Default DAD/Integration

Chromeleon (c) Dionex
Version 7.2.2.6686

Figure S36. HPLC analysis of compound **7**

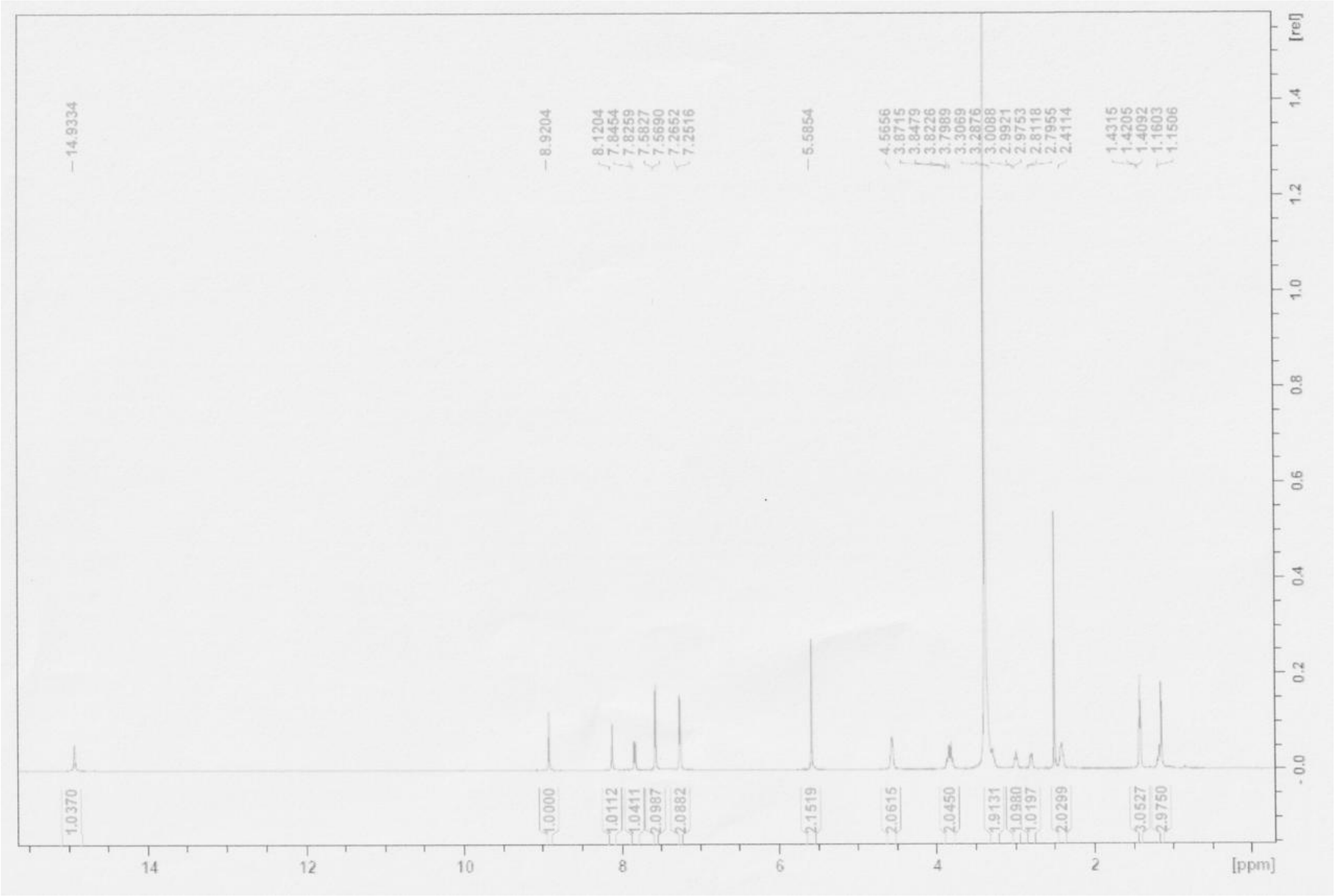

Figure S37. ^{1}H NMR spectra of compound **8**

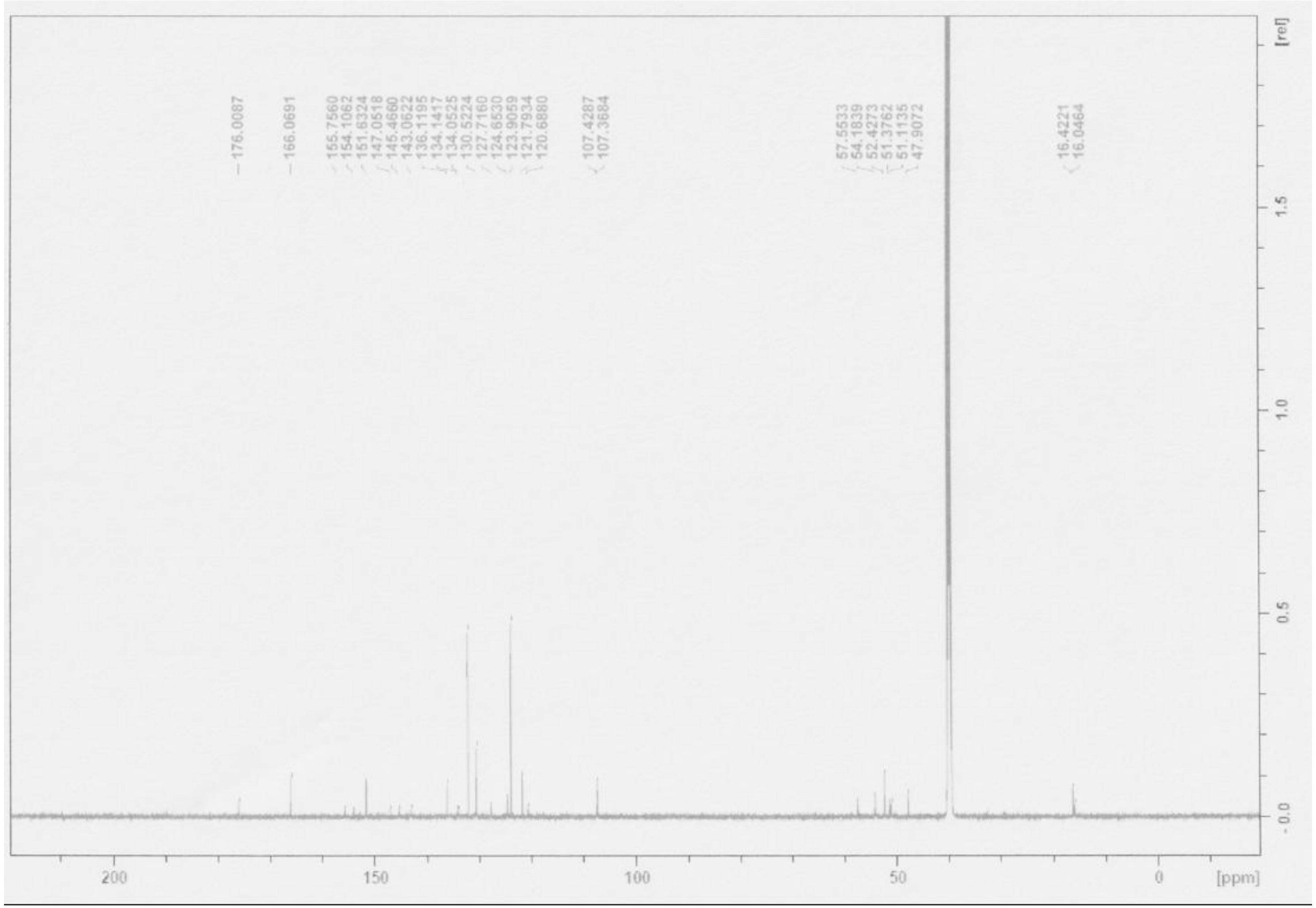

Figure S38. ^{13}C NMR spectra of compound **8**

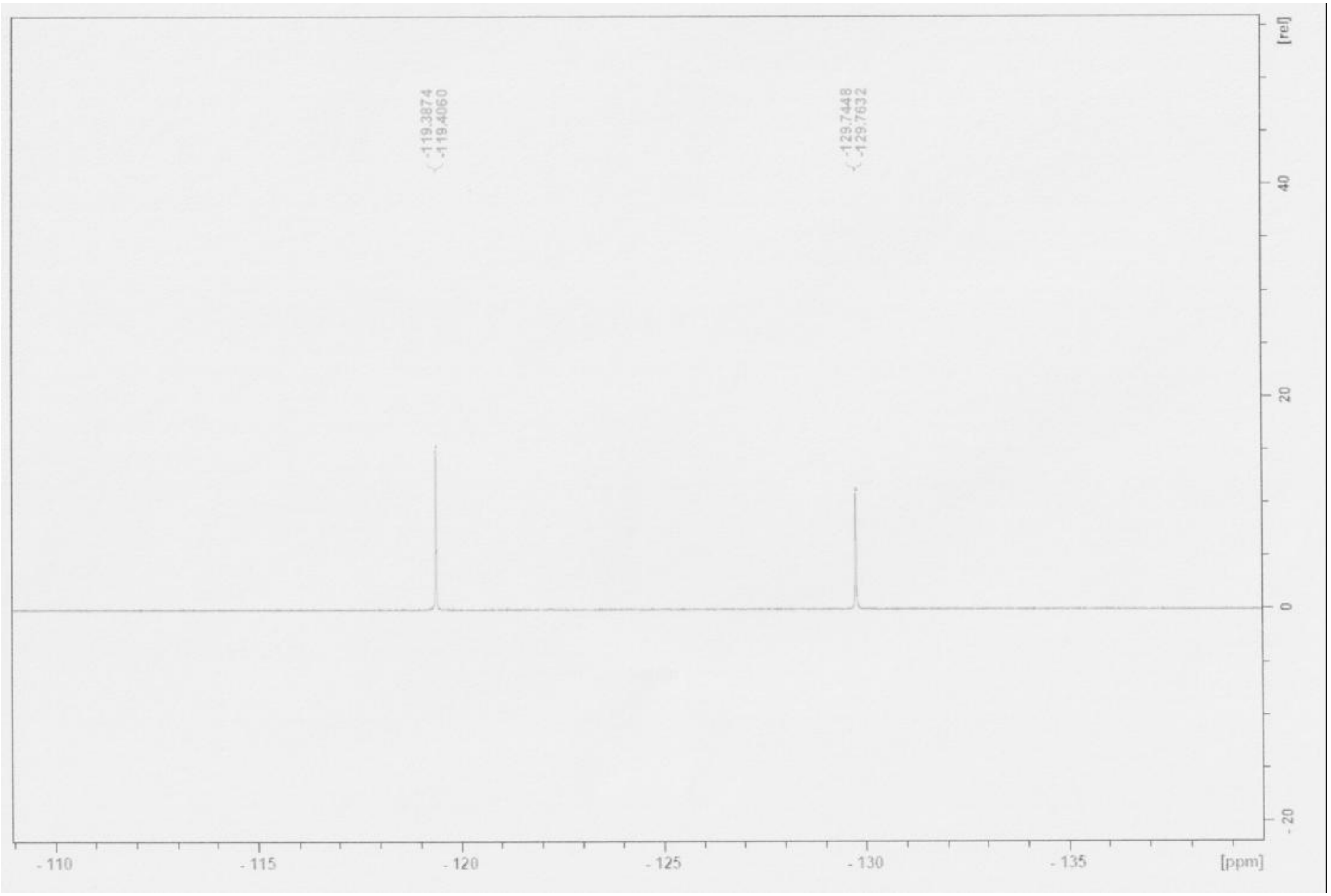

Figure S39. ^{19}F NMR spectra of compound **8**

Compound Spectrum List Report

Analysis Info | Acquisition Date 4/8/2025 1:02:37

Analysis Name	D:\Data\Iome_N_benzyl_Br.d		
Method	low_mass_positive.m	Operator	KM
Sample Name	Tune_neg_high_post	Instrument	impact II 18252
Comment			

Acquisition Parameter

Source Type	ESI	Ion Polarity	Positive	Set Nebulizer	0.3
Focus	Active	Set Capillary	4000 V	Set Dry Heater	200
Scan Begin	100 m/z	Set End Plate Offset	-500 V	Set Dry Gas	3.0
Scan End	1000 m/z	Set Charging Voltage	2000 V	Set Divert Valve	Sou
		Set Corona	0 nA	Set APCI Heater	0 °C

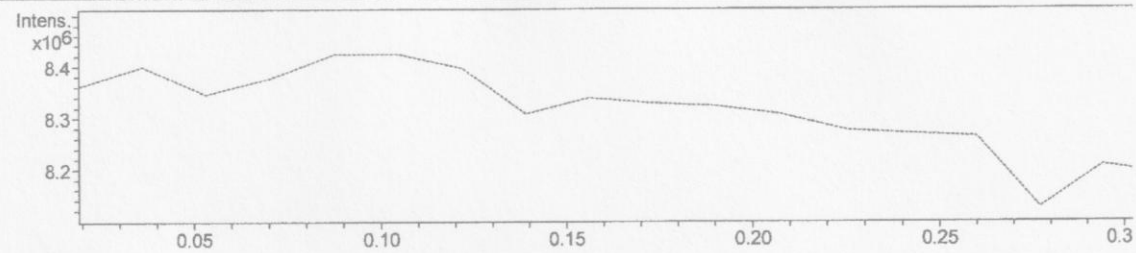

+MS, 0.0-0.3min #2-19

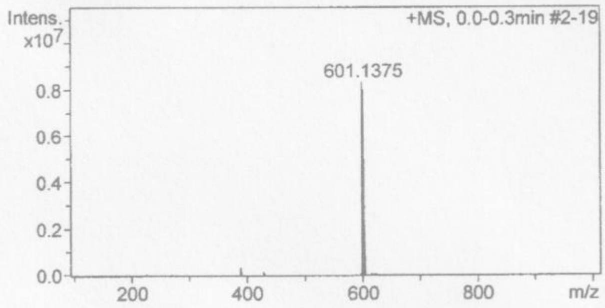

#	m/z	Res.	S/N	I	I %	FWHM
1	601.1375	44391	15364.1	8299865	100.0	0.0135
2	602.1398	30509	4127.1	2231703	26.9	0.0197
3	603.1359	41871	14749.4	7989902	96.3	0.0144
4	604.1380	30037	3908.8	2120384	25.5	0.0201
5	605.1403	20432	658.1	357665	4.3	0.0296

Iome_N_benzyl_Br.d
Bruker Compass DataAnalysis 4.3 printed: 4/8/2025 1:05:58 PM by: KM Pag

Figure S40. HR MS spectra of compound **8**

Chromatogram and Results

Injection Details

Injection Name:	GD1	*Run Time (min):*	10.00
Vial Number:	RA2	*Injection Volume:*	5.00
Injection Type:	Unknown	*Channel:*	UV_VIS_2
Instrument Method:	HPLC 1 seria 0.1	*Bandwidth:*	2
Processing Method:	LC MS	*Dilution Factor:*	1.0000
Injection Date/Time:	16/Jul/26 11:10	*Sample Weight:*	1.0000

Chromatogram

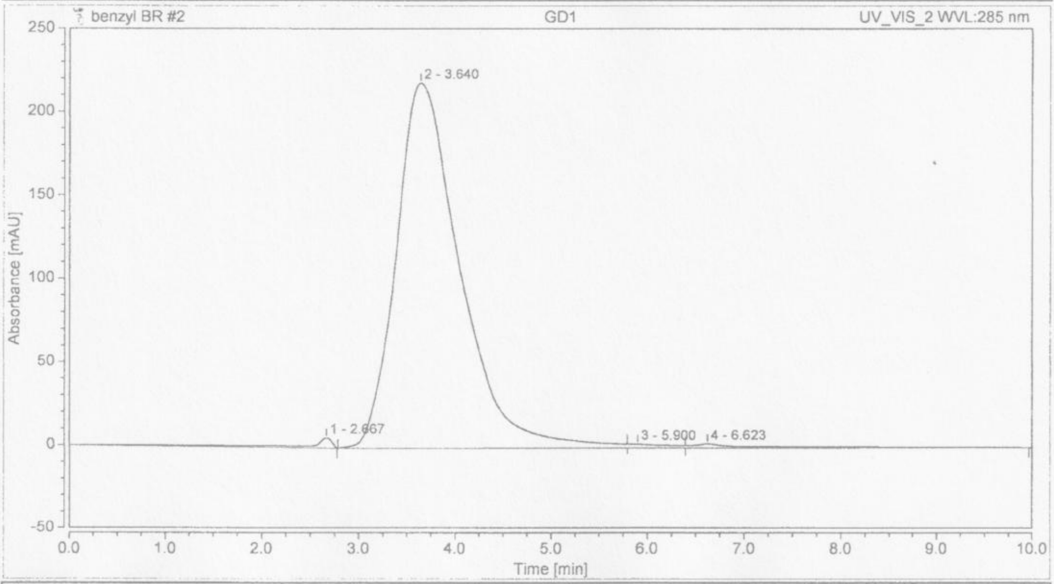

Integration Results

No.	Peak Name	Retention Time min	Area mAU*min	Height mAU	Relative Area %	Relative Height %	Amount n.a.
1		2.667	2.606	6.236	1.49	2.71	n.a.
2		3.640	168.543	219.305	96.52	95.15	n.a.
3		5.900	1.160	2.370	0.66	1.03	n.a.
4		6.623	2.302	2.569	1.32	1.11	n.a.
Total:			**174.611**	**230.480**	**100.00**	**100.00**	

Figure S41. HPLC analysis of compound **8**

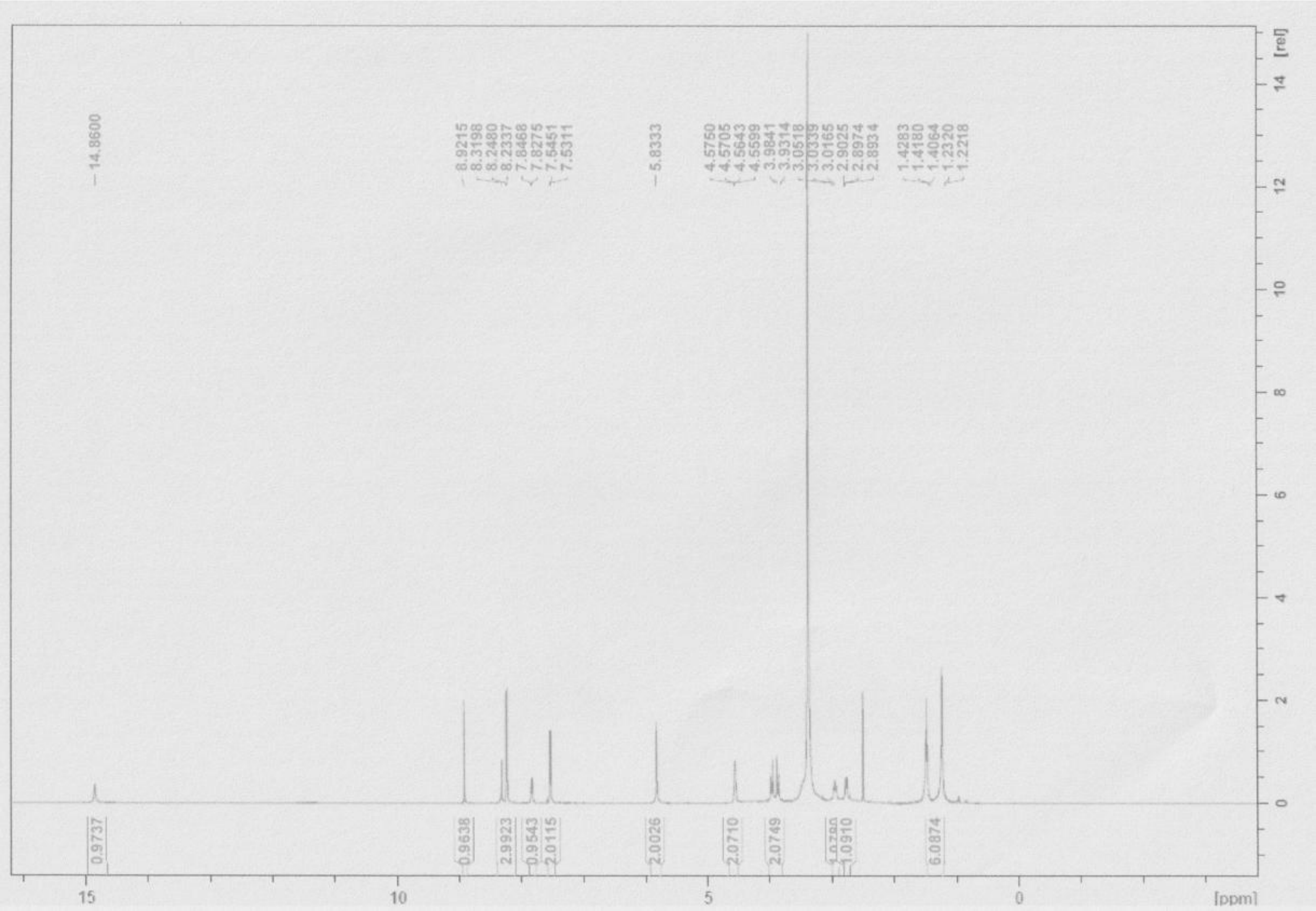

Figure S42. ^{1}H NMR spectra of compound **9**

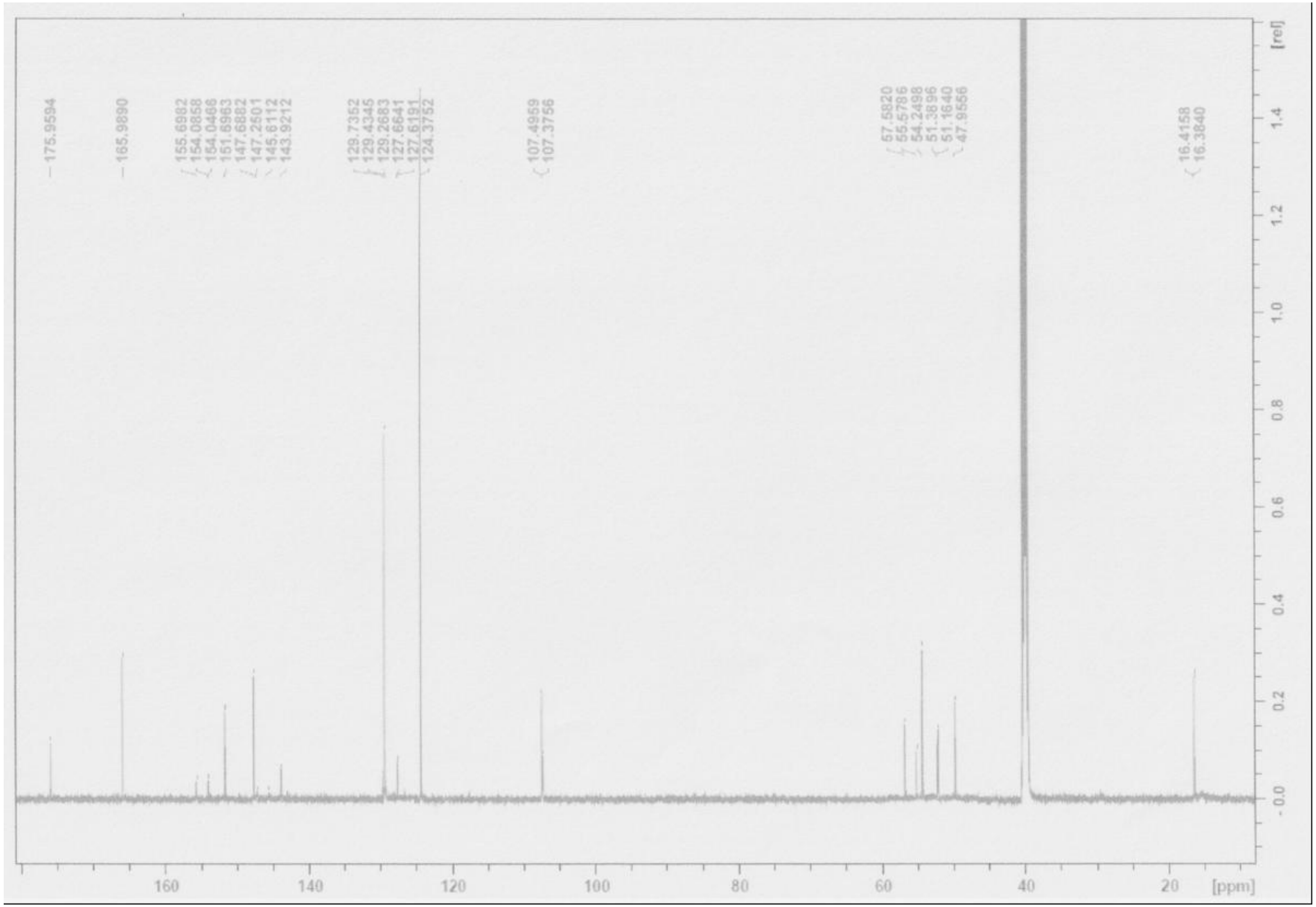

Figure S43. ^{13}C NMR spectra of compound **9**

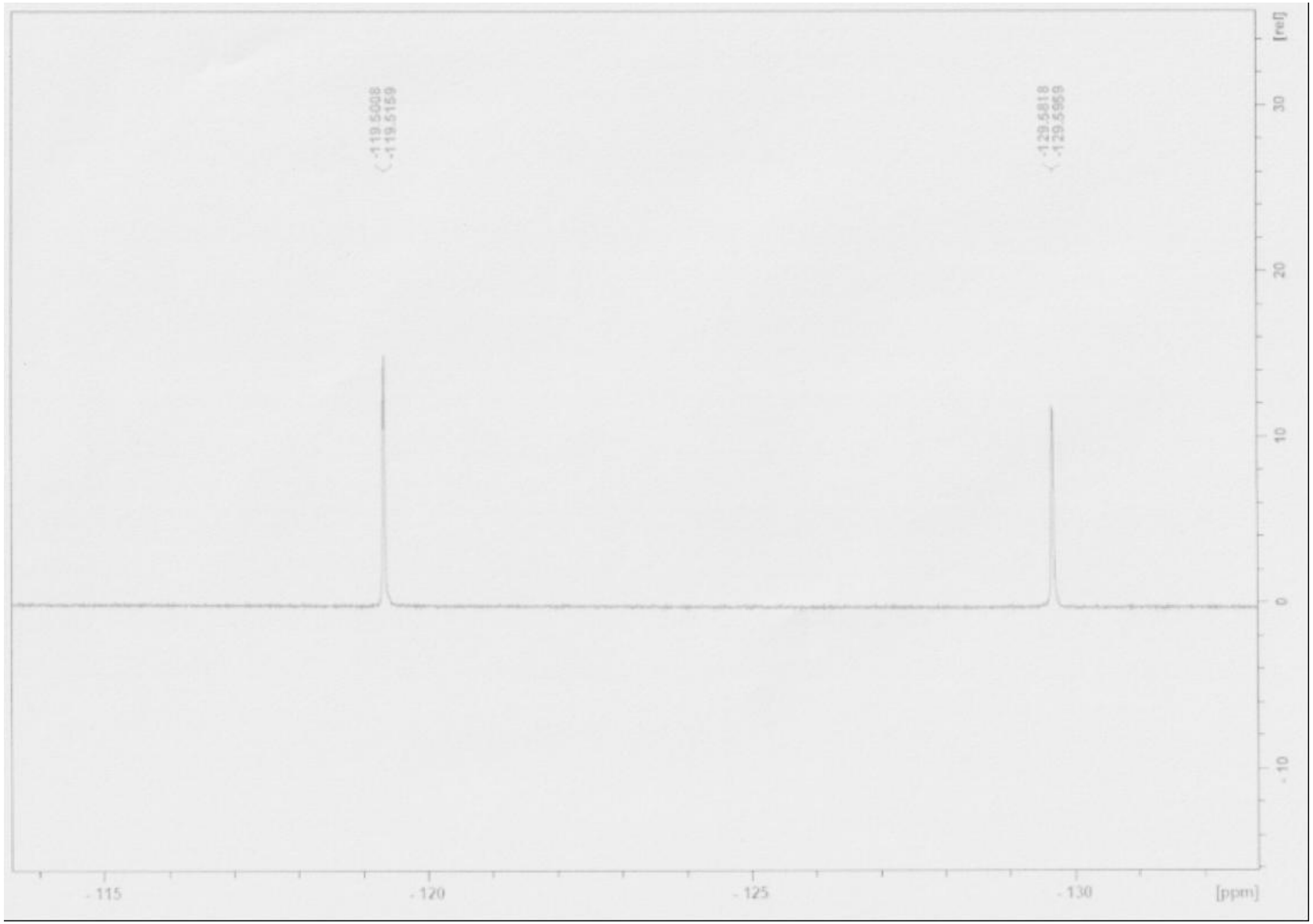

Figure S44. ^{19}F NMR spectra of compound **9**

Compound Spectrum List Report

Analysis Info

Analysis Name	D:\Data\Iome_N_benzyl_NO2.d	Acquisition Date	4/8/2025 1:17:53 PM
Method	low_mass_positive.m	Operator	KM
Sample Name	Tune_neg_high_post	Instrument	impact II 1825265.100
Comment			

Acquisition Parameter

Source Type	ESI	Ion Polarity	Positive	Set Nebulizer	0.3 Bar
Focus	Active	Set Capillary	4000 V	Set Dry Heater	200 °C
Scan Begin	100 m/z	Set End Plate Offset	-500 V	Set Dry Gas	3.0 l/min
Scan End	1000 m/z	Set Charging Voltage	2000 V	Set Divert Valve	Source
		Set Corona	0 nA	Set APCI Heater	0 °C

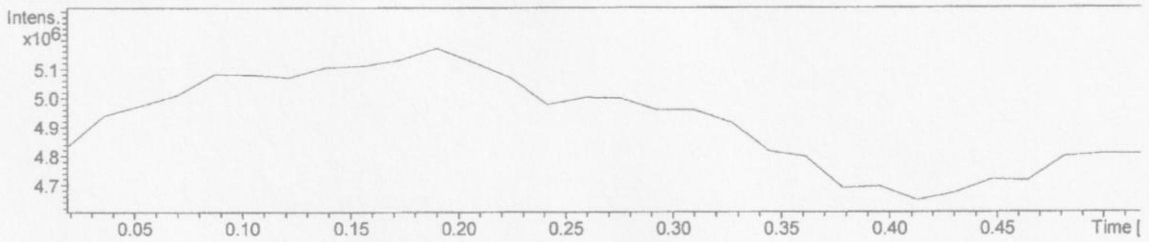

+MS, 0.0-0.5min #1-31

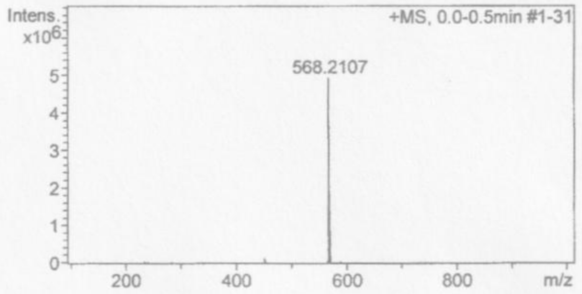

#	m/z	Res.	S/N	I	I %	FWHM
1	568.2107	38707	14107.4	4921312	100.0	0.0147
2	569.2132	26479	3983.6	1392918	28.3	0.0215
3	570.2155	20390	753.6	263533	5.4	0.0280

Figure S45. HR MS spectra of compound **9**

Chromatogram and Results

Injection Details

Injection Name:	GD1	*Run Time (min):*	10.00
Vial Number:	RA1	*Injection Volume:*	5.00
Injection Type:	Unknown	*Channel:*	UV_VIS_2
Instrument Method:	HPLC 1 seria 0.1	*Bandwidth:*	2
Processing Method:	LC MS	*Dilution Factor:*	1.0000
Injection Date/Time:	03/Jul/26 14:03	*Sample Weight:*	1.0000

Chromatogram

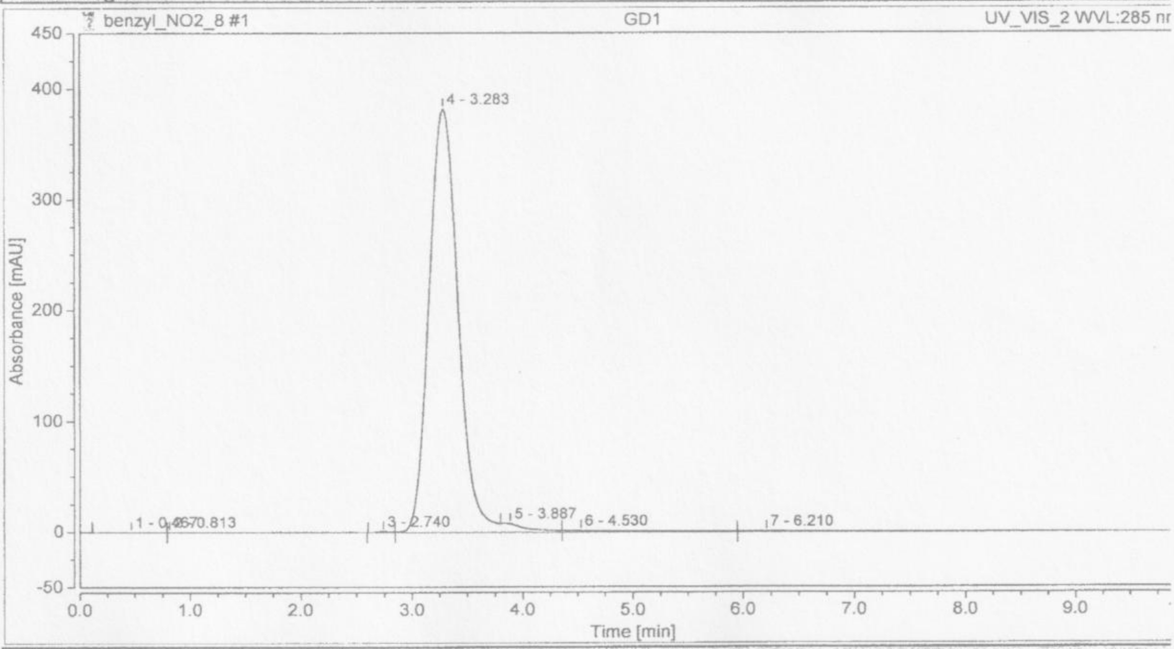

Integration Results

No.	Peak Name	Retention Time min	Area mAU*min	Height mAU	Relative Area %	Relative Height %	Amour n.a.
1		0.467	0.047	0.086	0.04	0.02	n.a.
2		0.813	0.380	0.129	0.31	0.03	n.a.
3		2.740	0.131	0.857	0.11	0.22	n.a.
4		3.283	116.080	381.922	95.81	97.16	n.a.
5		3.887	2.303	7.875	1.90	2.00	n.a.
6		4.530	1.371	1.491	1.13	0.38	n.a.
7		6.210	0.841	0.716	0.69	0.18	n.a.
**Total:**			**121.152**	**393.077**	**100.00**	**100.00**	

Figure S46. HPLC analysis of compound **9**
